# Recent Advances and Perspectives of Lewis Acidic Etching Route: An Emerging Preparation Strategy for MXenes

**DOI:** 10.1007/s40820-023-01039-z

**Published:** 2023-03-15

**Authors:** Pengfei Huang, Wei-Qiang Han

**Affiliations:** School of Materials Science and Engineering, Zhejiang University, Hangzhou, 310027 People’s Republic of China

**Keywords:** Lewis acidic etching, MXenes, Etching mechanism, Termination regulation, In-situ formed metals, Delamination, Application

## Abstract

Since the discovery in 2011, MXenes have become the rising star in the field of two-dimensional materials. Benefiting from the metallic-level conductivity, large and adjustable gallery spacing, low ion diffusion barrier, rich surface chemistry, superior mechanical strength, MXenes exhibit great application prospects in energy storage and conversion, sensors, optoelectronics, electromagnetic interference shielding and biomedicine. Nevertheless, two issues seriously deteriorate the further development of MXenes. One is the high experimental risk of common preparation methods such as HF etching, and the other is the difficulty in obtaining MXenes with controllable surface groups. Recently, Lewis acidic etching, as a brand-new preparation strategy for MXenes, has attracted intensive attention due to its high safety and the ability to endow MXenes with uniform terminations. However, a comprehensive review of Lewis acidic etching method has not been reported yet. Herein, we first introduce the Lewis acidic etching from the following four aspects: etching mechanism, terminations regulation, in-situ formed metals and delamination of multi-layered MXenes. Further, the applications of MXenes and MXene-based hybrids obtained by Lewis acidic etching route in energy storage and conversion, sensors and microwave absorption are carefully summarized. Finally, some challenges and opportunities of Lewis acidic etching strategy are also presented.

## Highlights

- As an emerging preparation strategy for MXenes, Lewis acidic etching has attracted increasing attention in the past few years benefiting from a series of merits.
- Lewis acidic etching method is mainly presented from etching mechanism, terminations regulation, in-situ formed metals and delamination of multi-layered MXenes.
- The applications of MXenes and MXene-based composites obtained by Lewis acidic etching route in energy storage and conversion, sensors and microwave absorption are carefully summarized.

## Graphical abstract

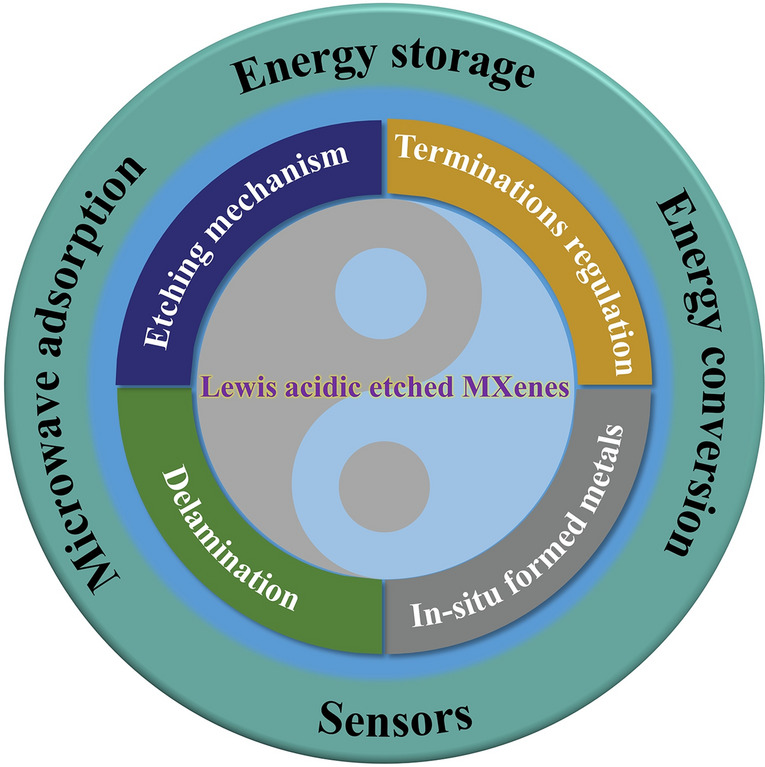

## Introduction

**Fig. 1:**
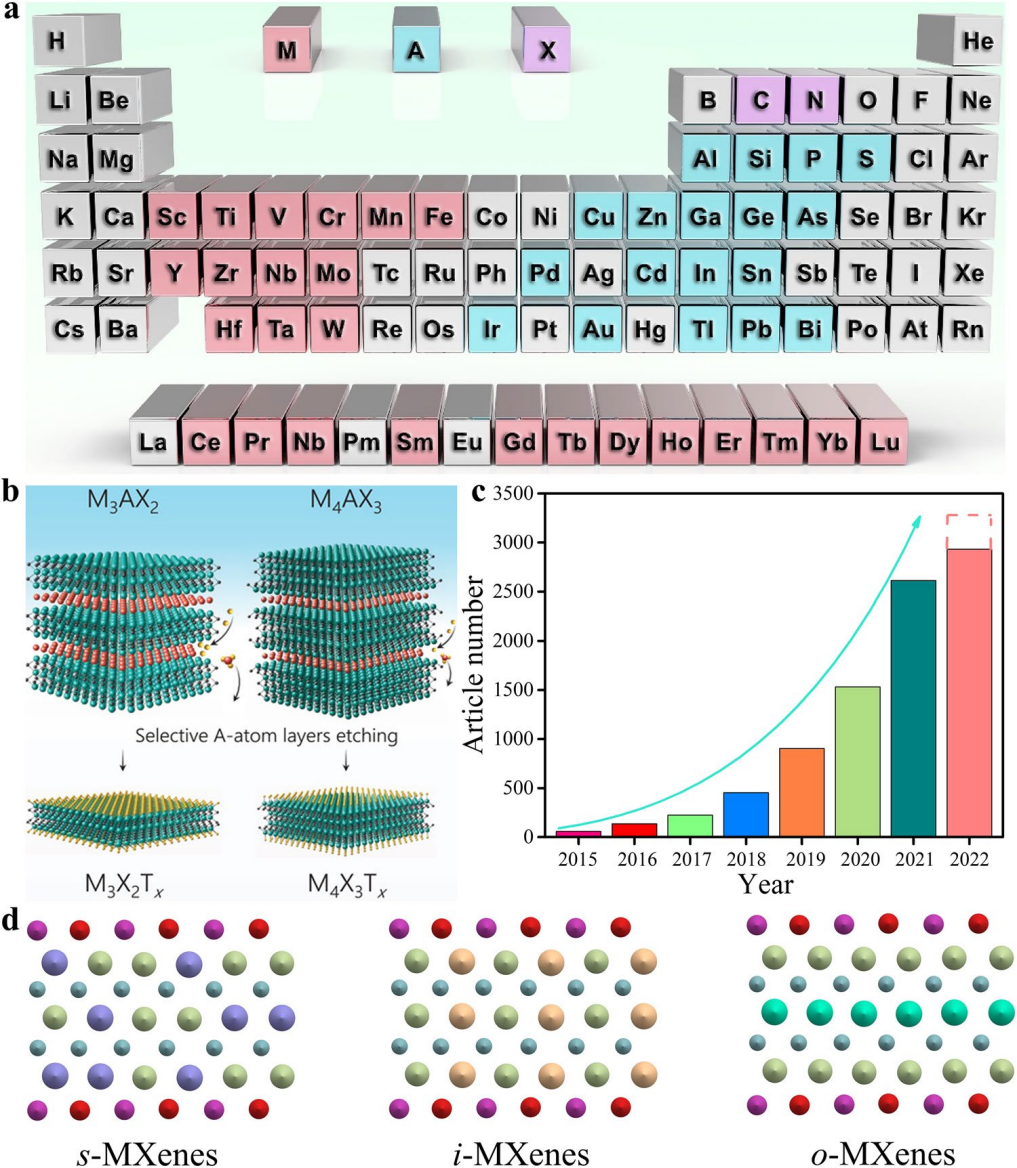
**a** Element composition of MAX phase. Reproduced with permission from [16] Copyright 2022, Elsevier. **b** Schematic illustration of the preparation of various MXenes from corresponding MAX phases. Reproduced with permission from [26] Copyright 2021, Wiley–VCH. **c** The annual published article numbers of MXenes. **d** Schematic of *s*-MXenes, *i*-MXenes and *o*-MXenes

The great success of graphene triggers enormous exploration in other two-dimensional (2D) materials, such as transition metal dichalcogenides, hexagonal boron nitride, layered double hydroxides (LDHs), graphitic carbon nitride, germanene, and black phosphorus [1–10]. In 2011, multi-layered Ti_3_C_2_T_*x*_ was first reported by Gogotsi and Barsoum’s group, which announces the birth of a brand-new and large family of 2D materials, namely transition metal carbides, nitrides and carbonitrides termed as MXenes [11–13]. They are generally derived from MAX precursors which possess a formula of M_*n*+1_AX_*n*_ (*n* = 1–4), where M stands for early transition metals (e.g., Ti, Ta, V, Mo, Nb, and Cr), A is mainly group 13 or 14 elements, such as Al, Si and Ga, and X refers to carbon or nitrogen (Fig. 1a) [14–16]. So far, 155 MAX phases have been successfully synthesized [17]. It is worth mentioning that some non-MAX ternary layered compounds such as Mo_2_Ga_2_C, Zr_3_Al_3_C_5_ and Hf_3_Al_3_C_5_ can also be used as precursors to prepare MXenes [18–21]. Structurally, MAX precursors possess typical hexagonal closely packed crystal structure with a P6_3_/*mmc* space group, and they are established by stacking of M_*n*+1_X_*n*_ building blocks that are interleaved with A-site atom layer along the *c* direction, leading to strong M-X covalent/metallic/ionic mixed bond and weak M-A metallic bond [21–23]. The difference in bond strength allows the A-site atoms to be selectively removed from MAX phase by a top-down method, and the remaining loosely stacked M_*n*+1_X_*n*_ architectures terminated with mixed surface groups T (e.g., –O, –OH, and –F) are named as MXenes, leading to a general chemical formula of M_*n*+1_X_*n*_T_*x*_ (*n* = 1–4, *x* ≤ 2) (Fig. 1b) [24–26].

**Fig. 2:**
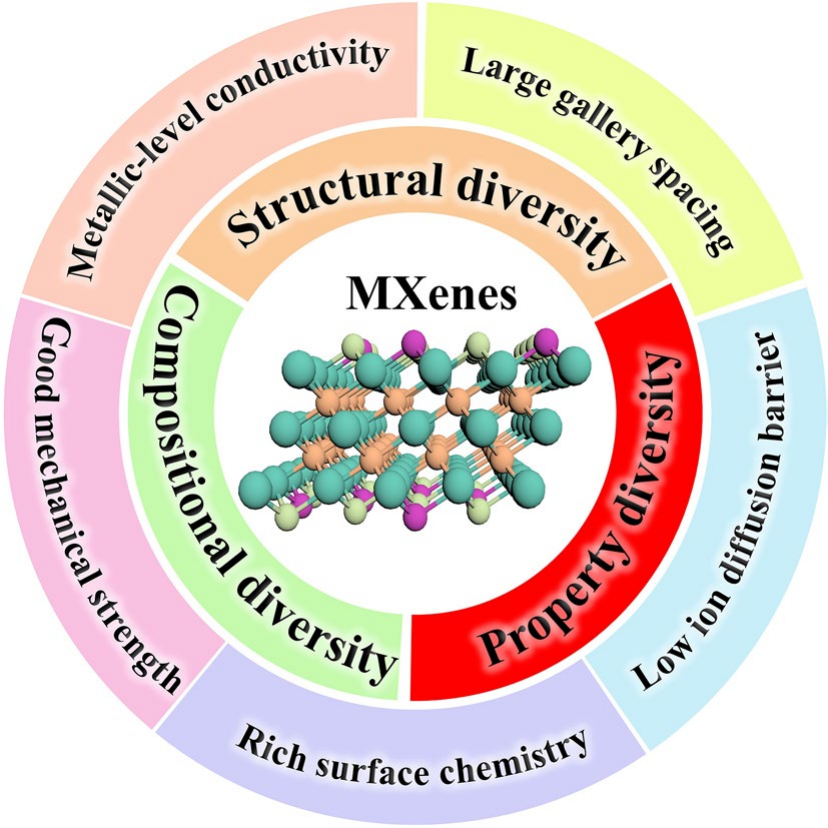
Schematic illustration of the structural, compositional and property diversity of MXenes

The publications of MXenes have increased significantly in the past eight years and reach around 2600 in 2021 (Fig. 1c). It is noteworthy that the majority of reported articles are focused on Ti_3_C_2_T_*x*_ MXene owing to its superior electronic conductivity (24,000 S cm^−1^) and large interlayer spacing [27–30]. Correspondingly, great progress has been achieved in the development of MXenes, especially in compositional and structural diversity. First, there are four kinds of MXenes with different stoichiometries including M_2_XT_*x*_, M_3_X_2_T_*x*_, M_4_X_3_T_*x*_ and M_5_X_4_T_*x*_ [27, 31–33]. Then, when the number of elements at the M-site exceeds two, MXenes can be categorized into three types according to the distribution of M-site atoms, namely *s*-MXenes with disordered arrangement, *i*-MXenes with in-plane ordering and *o*-MXenes with out-of-plane ordering (Fig. 1d) [15, 34–36]. Additionally, X-site atoms can be occupied by carbon, nitrogen or both [37–39]. Furthermore, the surface groups of MXenes mainly comprise of –O, –OH, –F, –Cl, –Br, –I, –S, –Se, –Te, –NH, etc., and can also be adjusted by etchants, termination substitution reaction and storage environment [40–43]. To date, nearly 50 various MXenes have been experimentally fabricated [44]. Considering the great tunability of M-site atoms, X-site atoms and surface terminations as well as the structural diversity of MXenes (Fig. 2), the number of MXenes will increase dramatically in the near future, eventually developing into a huge family [45].

MXenes generally exhibit a series of unique properties, including superior electronic conductivity, large and tunable gallery spacing, low ion diffusion barrier, rich surface chemistry, outstanding redox activity, hydrophilicity and wonderful mechanical strength (Fig. 2), making MXenes promising candidates for various fields [34, 45–48]. Specifically, large gallery spacing and rapid ion migration kinetics allow their use in high-performance metal-ion batteries and supercapacitors (SCs) [28, 39, 49, 50]. Flexibility, rich surface chemistry, adjustable interlayer spacing and high mechanical strength of MXenes enable them to be utilized as advanced separation membranes [51]. Excellent electronic conductivity and light weight of MXenes permit their apply in electromagnetic interference shielding, and ample redox centers endow them with superior catalysis capability [52–54]. Additionally, MXenes colloidal solution can be readily processed into freestanding papers and additive-free inks owing to the superior hydrophilicity [55, 56]. More importantly, the large compositional space, structural diversity and rich interlayer environment of MXenes offer great possibilities for unique combination and adjustability of properties (Fig. 2). For example, nitride MXenes display stronger polarity, higher electronic conductivity and more active sites compared with carbide counterparts, which renders them promising materials for secondary batteries [57]. Besides, high entropy MXenes such as (Ti_1/5_V_1/5_Zr_1/5_Nb_1/5_Ta_1/5_)_2_CT_*x*_ offers high mechanical strain owing to the distinct lattice distortions, which can effectively induce the uniform nucleation and deposition of Li metal [58]. Last but not least, the terminations play a key role in tuning the physicochemical characteristics of MXenes, especially in electronic properties. For example, MXenes without surface group possess high electron density near the Fermi level, while surface groups can generally alter the density of states and shift the Fermi level, finally resulting in semiconductor-like characteristics [59–61]. Furthermore, Nb_2_C MXene with –Cl, –S or –Se surface groups demonstrated superconductivity, whereas the O or F-terminated Nb_2_C fail to enter the superconducting state in the low-temperature region [42, 62]. In summary, MXenes have been widely explored in various fields up to now and the great tunability of electronic, optical, magnetic, mechanical and thermal properties would gradually expand their applications in the future [45, 63].

The effective synthesis strategy is able to adjust the properties of MXenes and make large-scale manufacturing possible [43, 64]. Until now, various preparation approaches for MXenes have been proposed. HF aqueous solution was first exploited to etch MAX precursors in 2011, which endows MXenes with distinct accordion-like morphology and mixed terminations of –OH, –O and –F [11]. However, the strong corrosiveness of HF prevents some researchers from participating in the research of MXenes. In 2014, Ghidiu et al. reported the in-situ formation of HF etchant by simply mixing LiF and HCl, which is able to achieve the etching of MAX precursor and improve the safety of experimental operation simultaneously [65]. Nevertheless, it is extremely challenging to prepare MXenes with uniform surface terminations by HF or LiF/HCl etching. Very recently, ZnCl_2_ Lewis acidic salt was employed to etch Ti_3_AlC_2_ MAX, which was based on the replacement reaction between Zn^2+^ cations and zero-valence Al of Ti_3_AlC_2_ MAX, finally leading to Zn metal-decorated multi-layered Ti_3_C_2_Cl_2_ MXene [66]. This strategy was further developed into a general route by using various Lewis acidic molten salts etchants to synthesize MXenes [67]. Compared with HF or LiF/HCl etching, Lewis acidic molten salts etching strategy demonstrates a series of advantages, such as greatly enhanced safety because of avoiding the direct or indirect use of hazardous HF, great universality to etch non-Al MAX precursors, the ability to endow MXenes with controllable surface terminations, easy accessibility to in-situ formed metals-modified MXenes and the potential for large-scale application.

**Fig. 3:**
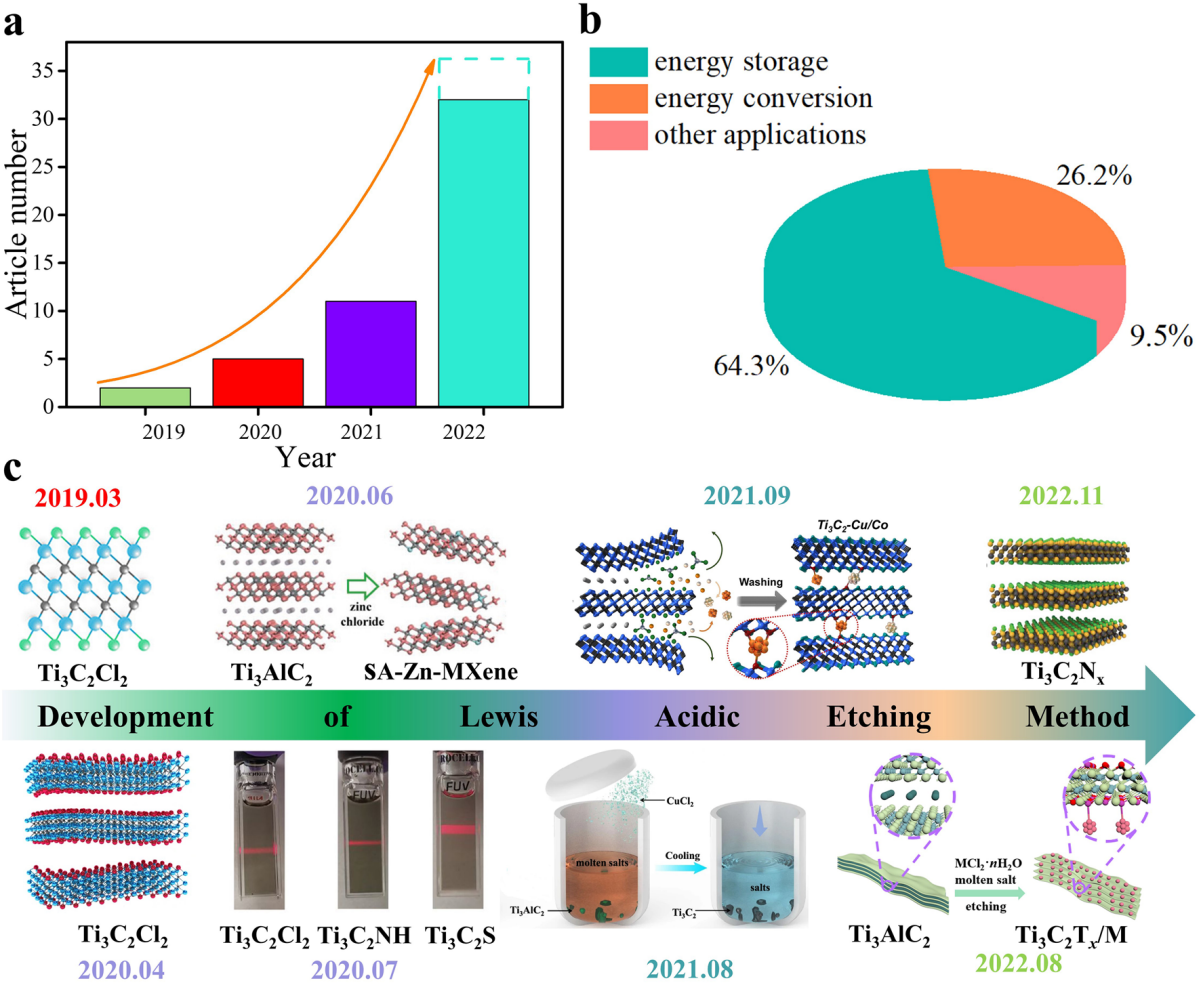
**a** Annual publications of Lewis acidic etching method. **b** Application ratio of MXenes and MXene-based composites prepared by Lewis acidic etching route in different fields. **c** A timeline showing the development progress of Lewis acidic etching route. Image published in 2019.03: Reproduced with permission from [66] Copyright 2019, American Chemical Society. Image published in 2020.04: Reproduced with permission from [67] Copyright 2020, The Authors, published by Springer Nature. Image published in 2020.06: Reproduced with permission from [157] Copyright 2020, Wiley–VCH. Image published in 2020.07: Reproduced with permission from [42] Copyright 2020, The American Association for the Advancement of Science. Image published in 2021.08: Reproduced with permission from [112] Copyright 2021, The Authors, published by Springer Nature. Image published in 2021.09: Reproduced with permission from [122] Copyright 2021, Wiley–VCH. Image published in 2022.08: Reproduced with permission from [147] Copyright 2022, Wiley–VCH. Image published in 2022.11: Reproduced with permission from [126] Copyright 2022, The Authors, published by Wiley–VCH

**Fig. 4:**
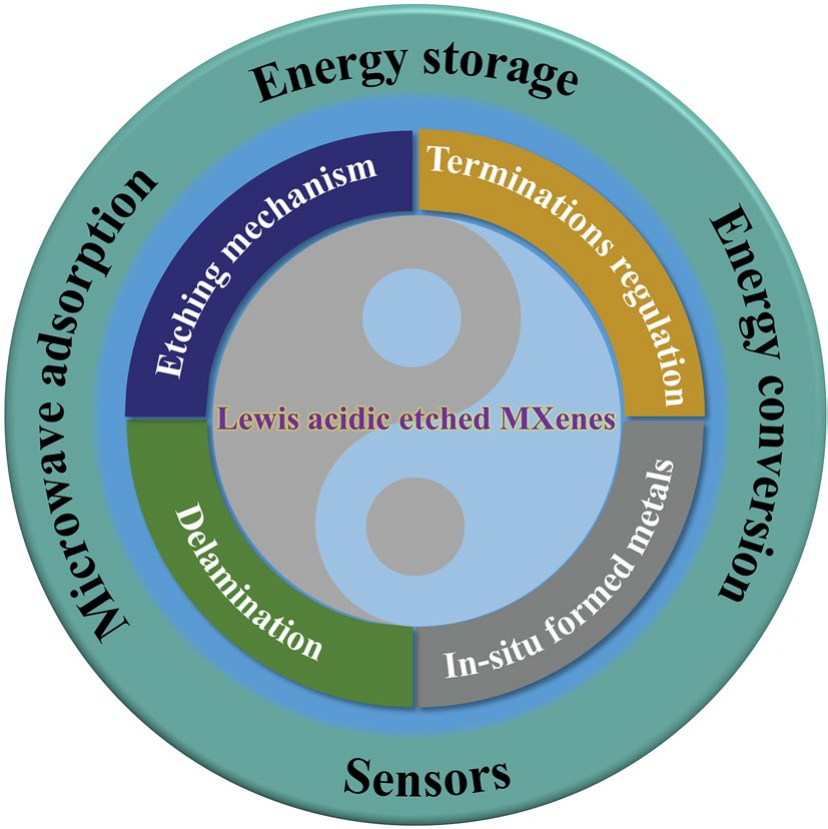
An overview demonstrating the Lewis acidic etching method

The Lewis acidic molten salts etching approach has received considerable attention since 2019 [66, 67], which can be proved by the increasing article numbers (Fig. 3a). In summary, the MXenes and MXene-based composites prepared by Lewis acidic etching route have been applied by researchers from different fields, and significant progress has been achieved in the development of Lewis acidic etching (Fig. 3b–c), showing great utilization potential. Therefore, it is highly required to summarize these advances timely. Nevertheless, to the best of our knowledge, there has been no review discussing the research progress of Lewis acidic molten salts etching approach. Herein, we systematically summarize the recent advances of Lewis acidic etching strategy. First, we briefly introduce the traditional synthesis techniques of MXenes based on non-Lewis acidic etching route, mainly including HF etching, in-situ HF etching, bifluoride salts etching, electrochemical etching, alkali etching, common molten salts etching, etc. Then, Lewis acidic etching strategy are mainly presented from the following four aspects: etching mechanism, terminations regulation, in-situ formed metals and delamination of multi-layered MXenes (Fig. 4). Subsequently, the applications of MXenes and MXene-based composites produced by Lewis acidic molten salts etching route in energy storage and conversion, sensors and microwave absorption have been comprehensively discussed. Finally, we also propose some perspectives for the challenges and opportunities of Lewis acidic etching. This review aims to provide some help in the future development of Lewis acidic molten salts etching strategy and enable more researchers to fully understand this emerging method, thereby promoting its widespread utilization.

## MXene Synthesis: Non-Lewis Acidic Etching Methods

### HF Etching

**Fig. 5:**
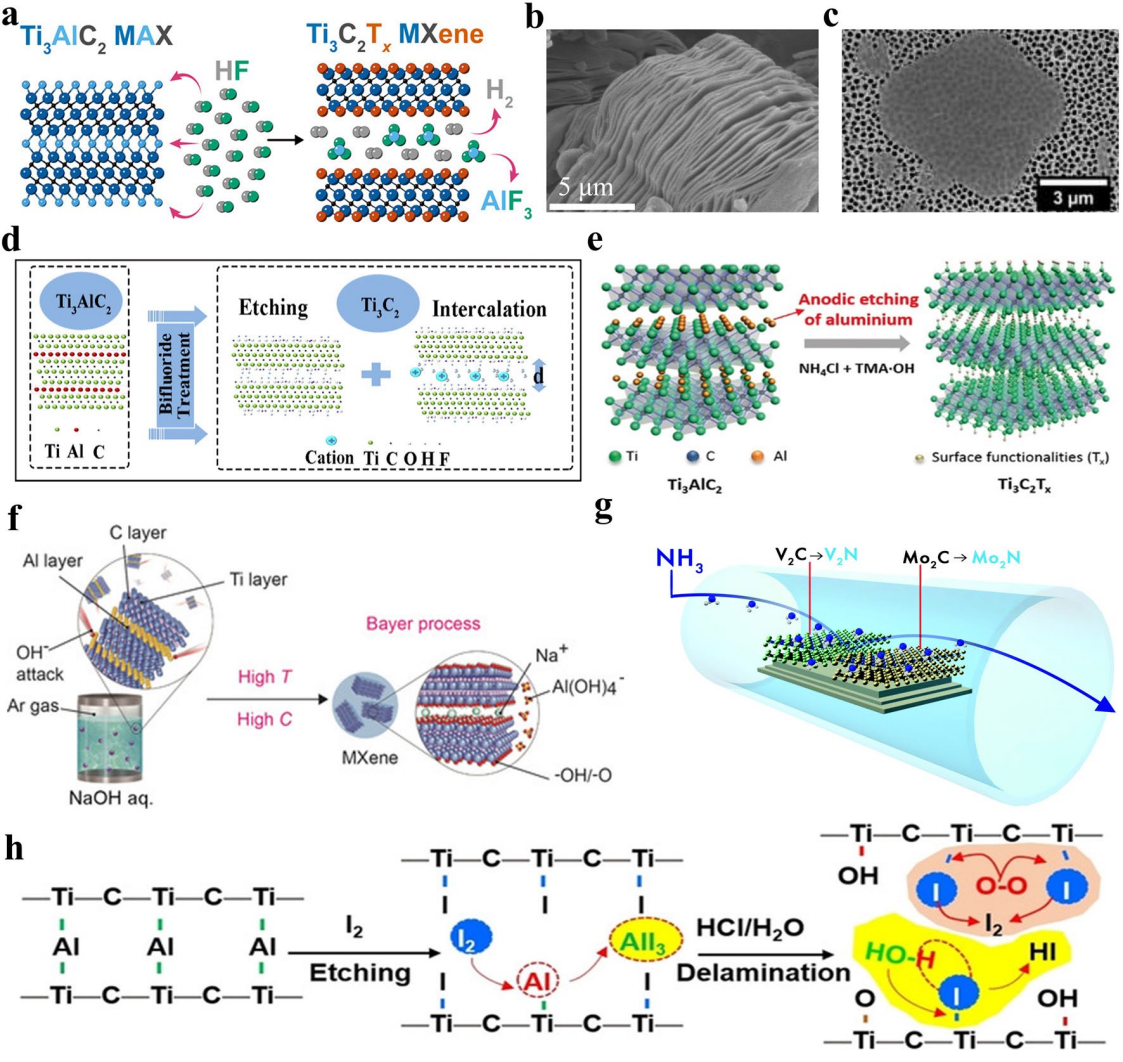
**a** Schematic showing the preparation of multi-layered Ti_3_C_2_T_*x*_ MXene by HF etching. Reproduced with permission from [70] Copyright 2022, American Chemical Society. **b** SEM image of multi-layered Ti_3_C_2_T_*x*_ MXene. Reproduced with permission from [72] Copyright 2020, Springer Nature. **c** SEM image of single-layered Ti_3_C_2_T_*x*_ MXene. Reproduced with permission from [82] Copyright 2021, American Chemical Society. **d** Schematic exhibiting the reaction mechanism between Ti_3_AlC_2_ and bifluorides. Reproduced with permission from [93] Copyright 2016, Elsevier. **e** Schematic of the electrochemical etching of Ti_3_AlC_2_ MAX in a binary aqueous electrolyte. Reproduced with permission from [98] Copyright 2018, Wiley–VCH. **f** Schematic of synthesis of Ti_3_C_2_T_*x*_ MXene by NaOH-assisted hydrothermal method. Reproduced with permission from [101] Copyright 2018, Wiley–VCH. **g** Synthesis of Mo_2_N and V_2_N MXene via ammoniation strategy. Reproduced with permission from [105] Copyright 2017, Royal Society of Chemistry. **h** Schematic diagram showing the preparation of Ti_3_C_2_T_*x*_ MXene via iodine-assisted etching. Reproduced with permission from [108] Copyright 2021, The Authors, published by Wiley–VCH

It is well known that graphene can be obtained by mechanical exfoliation of graphite because there is only weak van der Waals interaction between the layers [68, 69]. However, the metallic bond between M and A atoms in MAX precursors is stronger than van der Waals force, which makes it impossible to obtain MXenes via mechanical stripping of MAX precursors [21, 26]. Therefore, researchers have to explore other methods to prepare MXenes. In 2011, Gogotsi and Naguib et al. immersed Ti_3_AlC_2_ MAX precursor in HF aqueous solution at room temperature and the Al layers can be selectively removed owing to the large difference between Ti-C and Ti–Al bond strength, thus leading to the successful preparation of multi-layered Ti_3_C_2_T_*x*_ MXene with obvious accordion-like morphology (Fig. 5a–b) [11, 70–72]. For the HF etchant, H^+^ cations serve as oxidant and F^−^ anions act as ligand to combine with by-product of the reaction. The etching process is presented as follows:

1 $${\text{Ti}}_{{3}} {\text{AlC}}_{{2}} + {\text{3HF}} = {\text{Ti}}_{{3}} {\text{C}}_{{2}} + {\text{AlF}}_{{3}} + {3}/{\text{2H}}_{{2}}$$

2 $${\text{Ti}}_{{3}} {\text{C}}_{{2}} + {\text{2H}}_{{2}} {\text{O}} = {\text{Ti}}_{{3}} {\text{C}}_{{2}} \left( {{\text{OH}}} \right)_{{2}} + {\text{H}}_{{2}}$$

3 $${\text{Ti}}_{{3}} {\text{C}}_{{2}} + {\text{2HF}} = {\text{Ti}}_{{3}} {\text{C}}_{{2}} {\text{F}}_{{2}} + {\text{H}}_{{2}}$$

4 $${\text{Ti}}_{{3}} {\text{C}}_{{2}} + {\text{2H}}_{{2}} {\text{O}} = {\text{Ti}}_{{3}} {\text{C}}_{{2}} {\text{O}}_{{2}} + {\text{2H}}_{{2}}$$

Specifically, the almost zero-valence Al atoms in Ti_3_AlC_2_ precursor lose electrons to become Al^3+^ cations and then combine with F^−^ anions ligand to form soluble AlF_3_ [70, 71]. Simultaneously, the multi-layered Ti_3_C_2_ MXene is generally modified with –O, –OH and –F groups based on Eqs. (2–4). The introduction of surface functional groups results in the shift of (002) peak to lower angle, which can effectively confirm the successful preparation of MXenes [70, 73]. Since then, a series of MXenes such as Ti_2_CT_*x*_, Nb_2_CT_*x*_, V_2_CT_*x*_, Ti_3_CNT_*x*_ and Nb_4_C_3_T_*x*_ have been successfully prepared by HF etching [13, 74–77]. Additionally, the obtained multi-layered MXenes can be intercalated by large organic molecules such as dimethyl sulfoxide (DMSO), urea, tetramethylammonium hydroxide (TMAOH), isopropanol (IPA), isopropyl amine (i-PrA), tetrabutylammonium hydroxide (TBAOH), which further expands the gallery spacing and weakens the interlayer interaction [78–81], finally enabling the multi-layered MXenes to be delaminated into few-layered or single-layered MXenes (Fig. 5c) [82].

MAX precursors with different M-site atoms, A-site atoms, X-site atoms and* n* values usually require different HF concentration, etching time and reaction temperature. Generally, Mo-based and Nb-based MAX require higher HF concentration than Ti-based MAX to prepare corresponding MXenes [83, 84]. In addition, the etching of Si-based MAX including Ti_3_SiC_2_ requires HF and other strong oxidants (H_2_O_2_, KMnO_4_, etc.) to complete together [85]. Besides, compared with carbide MXenes, nitride MXenes require more precise adjustment of etching parameters during the preparation process, because the M–N bond may be broken under the attack of HF etchant [57]. However, harsh reaction environment could result in the formation of defects, such as Ti vacancy, which can adversely affect the electronic conductivity of MXenes [86]. Furthermore, some non-MAX precursors such as Zr_3_Al_3_C_5_, Hf_3_Al_4_C_6_ and Mo_2_Ga_2_C can also be etched by HF to obtain Zr_3_C_2_T_*x*_, Hf_3_C_2_T_*x*_ and Mo_2_CT_*x*_ MXene, respectively, which greatly expands the scope of precursors and is of great significance to the development of MXenes [18–20]. In general, HF etching method with excellent universality plays a crucial role in the rapid development of MXenes and some researchers still adopt this approach to prepare MXenes until now. Nevertheless, the strong corrosiveness of HF greatly increases the experimental risk and deteriorates its industrialization prospect. The development of a greener, safer and milder etchant is still the focus in the field of MXenes.

### In-Situ HF Etching

In 2014, a relatively safe etching route by mixing HCl and LiF was proposed by Ghidiu et al. to prepare Ti_3_C_2_T_*x*_ MXene [65]. In this approach, HF can be in-situ generated to initiate the etching process based on Eq. (5) and Li^+^ cations spontaneously intercalate into the interlayer of Ti_3_C_2_T_*x*_ MXene.

5 $${\text{HCl}} + {\text{LiF}} = {\text{HF}} + {\text{LiCl}}$$

The *c-*lattice parameter of MXenes obtained by LiF/HCl etching is greatly larger than that of MXenes prepared by HF etching, which is mainly owing to the intercalation of Li^+^ cations and water molecules. Benefiting from the hydrophilic nature, the prepared Ti_3_C_2_T_*x*_ MXene demonstrates clay-like behavior and can be processed into different desired shapes such as rolled film. Additionally, clay-like Ti_3_C_2_T_*x*_ MXene can be shaped into the letter “M” and exhibit a high electrical conductivity of 1500 S cm^−1^. More importantly, thanks to the insertion of Li^+^ ions and water, the van der Waals forces between the layers are weakened and multi-layered MXenes can be directly centrifuged after sonication to obtain few-layered MXenes. It is worth mentioning that the molar ratio of LiF and Ti_3_AlC_2_ MAX has a great influence on the lateral size of the final Ti_3_C_2_T_*x*_ MXene, and higher LiF concentration favors the complete etching of Ti_3_AlC_2_ MAX and intercalation of more Li^+^ ions into the MXene interlayers.

Therefore, in-situ HF etching is further upgraded to the “minimally intensive layer delamination” (MILD) method in 2017 by increasing the molar ratio of LiF and Ti_3_AlC_2_ MAX to 7.5:1.0, which can achieve the delamination of multi-layered MXenes via simple hand-shaking process rather than ultrasonication [87]. The obtained Ti_3_C_2_T_*x*_ MXene nanosheets demonstrate large lateral size and high quality, resulting in a high electronic conductivity of 4600 ± 1100 S cm^−1^. So far, other fluoride salts including NaF, KF, FeF_3_, etc., and acids such as H_2_SO_4_ have also been used by researchers [88–91]. For example, Soundiraraju et al. reported the successful preparation of Ti_2_NT_*x*_ MXene by immersing Ti_2_AlN MAX in a mixture of KF and HCl [89]. Generally, the in-situ HF etching method based on LiF/HCl etchant has appeared the most in the recently published articles on MXenes. The biggest advantage of in-situ HF etching route is the improvement of experimental safety and the ability to prepare MXenes nanosheets with larger lateral sizes and fewer defects. However, cations (Li^+^, Na^+^, K^+^, etc.) will intercalate into MXenes interlayers during the preparation process and it is difficult to eliminate them from MXenes, which may have an adverse impact on the further application of MXenes in some fields. The relationship between structure and properties should be further investigated.

### Bifluoride Salts Etching

In 2014, bifluoride NH_4_HF_2_ was used to etch the Ti_3_AlC_2_ MAX precursor to obtain Ti_3_C_2_T_*x*_ MXene [92]. The reaction mechanism is summarized as follows:

6 $${\text{Ti}}_{{3}} {\text{AlC}}_{{2}} + {\text{3NH}}_{{4}} {\text{HF}}_{{2}} = \left( {{\text{NH}}_{{4}} } \right)_{{3}} {\text{AlF}}_{{6}} + {\text{Ti}}_{{3}} {\text{C}}_{{2}} + {1}.{\text{5 H}}_{{2}}$$

7 $${\text{Ti}}_{{3}} {\text{C}}_{{2}} + a{\text{NH}}_{{4}} {\text{HF}}_{{2}} + b{\text{H}}_{{2}} {\text{O}} = {\text{Ti}}_{{3}} {\text{C}}_{{2}} \left( {{\text{OH}}} \right)_{x} {\text{F}}_{y} \left( {{\text{NH}}_{{3}} } \right)_{c} \left( {{\text{NH}}_{{4}} } \right)_{d}$$

In this method, the selective etching of Al layers from Ti_3_AlC_2_ MAX and the intercalation of NH_3_ and NH_4_^+^ into the interlayer of Ti_3_C_2_T_*x*_ MXene occur simultaneously. The intercalation of ammonium species effectively increases the interlayer spacing. Further, NaHF_2_ and KHF_2_ were also utilized as etchants to prepare Ti_3_C_2_T_*x*_ MXene by Feng et al. in 2017 and the etching mechanisms are similar to that of NH_4_HF_2_ [93] (Fig. 5d). In 2020, Natu et al. used NH_4_HF_2_ to etch Ti_3_AlC_2_ MAX with polar organic solvents as the reaction medium instead of water, successfully realizing the water-free synthesis of Ti_3_C_2_T_*x*_ MXene [94]. The NH_4_HF_2_ can be decomposed into HF and NH_4_F, and HF plays the role of etching the Al atomic layers. It is noteworthy that the as-prepared Ti_3_C_2_T_*x*_ MXene is highly fluorinated and the ammonium cations surrounded by organic solvent molecules can intercalate into the layers, which endows MXene with the largest interlayer spacing of 5.07 nm up to now. Overall, the bifluoride salts etching is safer relative to HF etching. However, bifluoride salts are currently only used to etch Ti_3_AlC_2_ MAX and the universality of the method requires to be further improved.

### Electrochemical Etching

During the etching process of MAX precursors by using the three methods mentioned above, the researchers may be directly or indirectly exposed to hazardous HF, which reduces the safety of the experimental process. Moreover, high content of -F surface group may reduce the electronic conductivity of obtained MXenes and block ion transport, which is not conducive to their application in metal-ion batteries [59]. Therefore, it is urgent to explore fluorine-free etching method. In 2014, Gogotsi et al. removed M-atom and A-atom layers from Ti_3_AlC_2_, Ti_2_AlC and Ti_3_SiC_2_ MAX precursors to prepare carbide-derived carbons via electrochemical etching approach [95]. In 2015, Zhao et al. selectively extracted Ti atom layers from Ti_2_SC MAX to obtain carbon/sulfur nanolaminates through electrochemical etching [96]. It can be observed that the above two works failed to obtain MXenes by electrochemical etching method. In 2017, Sun et al. firstly synthesized Ti_2_CT_*x*_ MXene without -F termination in diluted HCl aqueous electrolyte at 0.6 V for 5 days via electrochemical etching [97], and the mechanism can be exhibited as follows:

8 $${\text{Ti}}_{{2}} {\text{AlC}} + y{\text{Cl}}^{ - } + \left( {{2}x + z} \right){\text{H}}_{{2}} {\text{O}} \to {\text{Ti}}_{{2}} {\text{C}}\left( {{\text{OH}}} \right)_{{{2}x}} {\text{Cl}}_{y} {\text{O}}_{z} + {\text{Al}}^{{{3} + }} + \left( {x + z} \right){\text{H}}_{{2}} + \left( {y + { 3}} \right){\text{e}}^{ - }$$

9 $${\text{Al}}^{{{3} + }} + {\text{3e}}^{ - } \to {\text{Al}}$$

At first, only the surface of Ti_2_AlC MAX that is in contact with the electrolyte can be etched and the generated Ti_2_CT_*x*_ MXene is coated on the surface of MAX, which prevents electrolyte from entering the interior and makes it difficult for the inner MAX to be further etched. Subsequently, the Ti_2_CT_*x*_ MXene on the surface will be transformed into a carbon layer. Finally, a three-layered structure including carbide-derived carbon, Ti_2_CT_*x*_ MXene and unetched Ti_2_AlC MAX are obtained, thus leading to a low yield of MXenes. Some key parameters such as electrolyte concentration, etching potential and time have been proven to have a direct impact on the final etching products.

In 2018, Feng et al. prepared Ti_3_C_2_T_*x*_ MXene with a large lateral size of 18.6 μm in a binary aqueous electrolyte containing NH_4_Cl and TMAOH (Fig. 5e) [98]. In this method, the weak Ti–Al bond is broken and then Al^3+^ cations combine with Cl^−^ anions to form AlCl_3_. The obtained Ti_3_C_2_ can be terminated by –OH group and the etching process is illustrated as follows:

10 $${\text{Ti}}_{{3}} {\text{AlC}}_{{2}} {-}{\text{3e}}^{ - } + {\text{3Cl}}^{ - } = {\text{Ti}}_{{3}} {\text{C}}_{{2}} + {\text{AlCl}}_{{3}}$$

11 $${\text{Ti}}_{{3}} {\text{C}}_{{2}} + {\text{2OH}}^{ - } {-}{\text{2e}}^{ - } = {\text{Ti}}_{{3}} {\text{C}}_{{2}} \left( {{\text{OH}}} \right)_{{2}}$$

12 $${\text{Ti}}_{{3}} {\text{C}}_{{2}} + {\text{2H}}_{{2}} {\text{O}} = {\text{Ti}}_{{3}} {\text{C}}_{{2}} \left( {{\text{OH}}} \right)_{{2}} + {\text{H}}_{{2}}$$

It is noteworthy that the intercalation of ammonium species and TMAOH can expand the interlayer spacing of Ti_3_C_2_T_*x*_ MXene and expose more active sites, greatly promoting the etching of inner MAX phase and thus resulting in a relatively high etching yield of at least 60%. Later, a general strategy based on thermal-assisted electrochemical etching route was proposed by Hao’s group to synthesize various MXene (Ti_2_CT_*x*_, Cr_2_CT_*x*_ and V_2_CT_*x*_) [99]. In this approach, 1 M HCl solution is chosen as electrolyte and the heating facilitates the etching of MAX precursor. Specifically, when the etching time and temperature is increased to 9 h and 50 °C, respectively, the obvious accordion-like morphology can be observed for Ti_2_CT_*x*_ MXene. In general, the electrochemical etching enables the preparation of fluorine-free MXenes for the first time and is safer and milder than conventional HF etching method. However, over-etching and low etching yield are unavoidable issues, which greatly hinders the large-scale utilization of electrochemical etching method.

### Alkali Etching

In addition to acids, alkalis can also be utilized to selectively etch MAX precursors. The earliest attempt to prepare MXenes via alkali etching was to immerse Ti_3_AlC_2_ MAX in dilute aqueous NaOH solution at 80 °C by Xie et al. in 2014 [100]. However, only the outermost MAX can be etched owing to the low NaOH concentration. Further, Zhang et al. proposed a NaOH-assisted hydrothermal process in 2018 to prepare Ti_3_C_2_T_*x*_ MXene with a high yield of 92% (Fig. 5f) [101]. In this method, the OH^−^ anions attack Al-atom layers in Ti_3_AlC_2_ MAX to form Al (oxide) hydroxides, which can further dissolve in alkali solution as soluble Al(OH)_4_^−^ anions. It is noteworthy that high reaction temperature (270 °C) and NaOH concentration (27.5 M) can greatly speed up the whole process and promote complete etching of Ti_3_AlC_2_ MAX. The etching process can be described as follows:

13 $${\text{Ti}}_{{3}} {\text{AlC}}_{{2}} + {\text{OH}}^{ - } + {\text{5H}}_{{2}} {\text{O}} = {\text{Ti}}_{{3}} {\text{C}}_{{2}} \left( {{\text{OH}}} \right)_{{2}} + {\text{Al}}\left( {{\text{OH}}} \right)_{{4}}^{ - } + {2}.{\text{5H}}_{{2}}$$

14 $${\text{Ti}}_{{3}} {\text{AlC}}_{{2}} + {\text{OH}}^{ - } + {\text{5H}}_{{2}} {\text{O}} = {\text{Ti}}_{{3}} {\text{C}}_{{2}} {\text{O}}_{{2}} + {\text{Al}}\left( {{\text{OH}}} \right)_{{4}}^{ - } + {3}.{\text{5H}}_{{2}}$$

It is worth mentioning that special attention should be paid to controlling reaction condition when MXene is prepared by alkali etching method. A recent work by our group found that Ti_3_C_2_T_*x*_ MXene can be completely converted to K_2_Ti_8_O_17_ nanowires after immersed in 5 M KOH solution at 50 °C for 10 days [102]. The reaction mechanism can be described as follows: The Ti-C bond in Ti_3_C_2_T_*x*_ MXene is easily destroyed in the concentrated alkali environment and MXene is oxidized into titanium oxide nanoparticles, and the titanium oxide nanoparticles can then react with KOH to generate the final K_2_Ti_8_O_17_ nanowires. Accordingly, the stability of various MXenes in concentrated alkali solution deserves further investigation. In general, although MXenes with abundant –O and –OH functional groups can be prepared via alkali etching route, harsh reaction condition greatly increases the experimental risk, which makes alkali etching unsuitable for large-scale preparation of MXenes.

### Common Molten Salts Etching

The above-mentioned methods for preparing MXenes are all based on the wet chemical etching route. However, Gogotsi et al. reported molten salts etching method to prepare Ti_4_N_3_T_*x*_ MXene in 2016, which opens the door to the preparation of MXenes via dry chemical etching techniques [103]. In this method, a mixture of LiF, NaF and KF is used to selectively etch Al-atom layers from Ti_4_AlN_3_ MAX precursor at 550 °C under Ar atmosphere to synthesize Ti_4_N_3_T_*x*_ MXene, and the few-layered nanoflakes can be obtained by TBAOH intercalation and subsequent ultrasonication. It is worth mentioning that since the discovery of MXenes, Ti_4_N_3_T_*x*_ is the first nitride MXene that has been experimentally synthesized. In 2021, Zong et al. reported the synthesis of Ti_2_NT_*x*_ MXene by etching Ti_2_AlN MAX precursor with the mixture of fluoride salt (KF, LiF, and NaF) in a molar ratio of 1:3 at 550 °C [104]. Up to now, the family of nitride MXenes only contains Ti_2_N, Mo_2_N, V_2_N and Ti_4_N_3_ [89, 103, 105], which is mainly due to the difficulty in preparation. Firstly, the number of nitride MAX precursor is relatively limited due to the high formation energy. Then, M-A bond in nitride MAX exhibits high strength compared with that of carbide MAX, indicating that larger exfoliation energy is needed for nitride MAX. Finally, nitride MXene is prone to dissolve in HF solution and exhibits poor structural stability owing to the low cohesive energy [38, 71]. Therefore, great efforts should be made to explore the suitable etching route for nitride MAX. In general, molten salts etching, as the first method to prepare nitride MXenes, is of great significance. However, this method is only applicable to the preparation of Ti_4_N_3_T_*x*_ and Ti_2_NT_*x*_ MXene at present, and the obtained MXene exhibits high content of -F surface group. The above two key points greatly limit the development of common molten salts etching method.

### Other Etching Methods

In addition to the etching methods described above, there are other methods that can also be used to prepare MXenes. In 2017, Gogotsi’s group presented an ammoniation strategy to transform Mo_2_C and V_2_C MXene to corresponding Mo_2_N and V_2_N at 600 °C by using NH_3_ as nitrogen source (Fig. 5g) [105], which further enriches the family of nitride MXenes. It is worth mentioning that the obtained Mo_2_N and V_2_N MXene demonstrate higher room temperature electronic conductivity than their carbide counterparts. In 2020, Sun et al. successfully prepared mesoporous Mo_2_C MXene via ultraviolet-driven etching of Mo_2_Ga_2_C precursor, which avoids the utilization of hazardous HF and greatly improves the experimental safety [106]. They also presented thermal reduction strategy to synthesize Ti_2_C MXene from Ti_2_SC MAX. In this method, reductive gas such as H_2_ can react with weakly-bonded S atoms, continuously accelerating the etching process [107]. In 2021, iodine-assisted etching route was developed by Feng et al. to synthesize Ti_3_C_2_T_*x*_ MXene rich in -O terminations in anhydrous acetonitrile at 100 °C (Fig. 5h) [108]. Owing to the water-free etching process, Ti_3_C_2_T_*x*_ MXene with an average lateral size of 1.8 µm exhibits superior structural integrity and can be stored stably in water for more than 2 weeks. Further, Jawaid et al. successfully prepared halogenated Ti_3_C_2_T_*x*_ MXene under anhydrous environment by using halogens (Br_2_, I_2_, ICl, IBr) as etchants, and the halogen terminations may endow MXenes with some new properties [109]. Besides, ionic liquids such as 1-butyl-3-methylimidazolium hexafluoro-phosphate (BMIMPF_6_) was utilized as etchant to prepare Ti_2_CT_*x*_ MXene at 80 °C for 20 h [110]. The ionic liquids can intercalate into interlayers, leading to expanded interlayer spacing and the separation of layers.

Compared with above-mentioned top-down methods, the bottom-up approaches are usually able to synthesize defect-free 2D materials, which can also be utilized to obtain high-quality MXenes. In 2015, Ren et al. developed chemical vapor deposition method to fabricate large-area high-quality ultrathin α-Mo_2_C nanosheets, which possess a thickness of few nanometers and a lateral size of around 100 µm [111]. Moreover, the prepared α-Mo_2_C MXene exhibits superior stability under various environments including water, HCl or annealing at 200 °C in air. More importantly, this method reveals excellent generality and can also be used to construct WC and TaC crystals. In conclusion, many various methods have been proposed to prepare MXenes until now. However, each etching route has its own shortcomings and is unlikely for large-scale utilization in the future. As we all know, exploring a safe, green, non-hazardous, low-cost and easily scalable preparation method is the biggest challenge for the researchers in the fields of MXenes, which plays a crucial role in the development of MXenes in the next decades.

## MXene Synthesis: Lewis Acidic Etching Method

In 2019, Huang’s group proposed a brand-new, safe and green method, namely Lewis acidic molten salts etching, to prepare Cl-terminated MXene [66]. Owing to a series of advantages, Lewis acidic etching has attracted intensive attention since it was presented, greatly promoting the development of MXenes in the past three years. The introduction of Lewis acidic molten salts etching approach is mainly classified into following four aspects: etching mechanism, terminations regulation, in-situ formed metals and delamination of multi-layered MXenes.

### Etching Mechanism

**Fig. 6:**
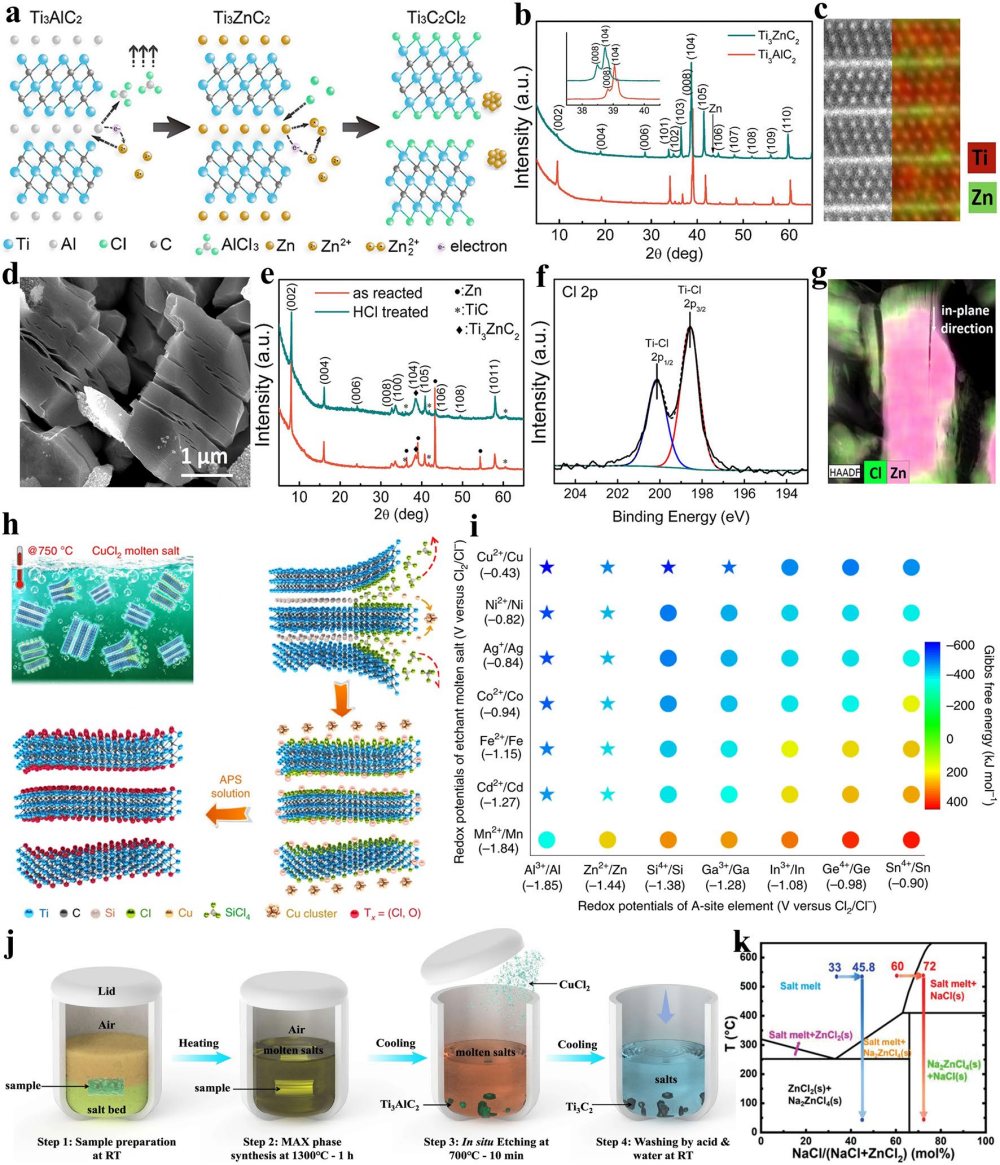
**a** Schematic showing the preparation of Ti_3_C_2_Cl_2_ MXene by ZnCl_2_ Lewis acidic salt etching. **b** XRD patterns of Ti_3_AlC_2_ and Ti_3_ZnC_2_ MAX. **c** HAADF-STEM image of Ti_3_ZnC_2_ MAX and corresponding energy-dispersive spectroscopy (EDS) mapping. **d** SEM image of multi-layered Ti_3_C_2_Cl_2_ MXene. **e** XRD patterns of Ti_3_C_2_Cl_2_ MXene and Ti_3_C_2_Cl_2_/Zn hybrids. **f** Cl 2*p* XPS spectra of Ti_3_C_2_Cl_2_ MXene. **g** EDS mapping analysis showing the Lewis acidic etching of Ti_3_AlC_2_ MAX. Reproduced with permission from [66] Copyright 2019, American Chemical Society. **h** Schematic demonstrating the preparation of Ti_3_C_2_T_*x*_ MXene by CuCl_2_ etching. **i** Schematic showing the relationship between redox potential of A-site elements, redox potential of Lewis acidic salts and Gibbs free energy. Reproduced with permission from [67] Copyright 2020, The Authors, published by Springer Nature. **j** Schematic illustration of the one-pot synthesis of Ti_3_C_2_T_*x*_ MXene under air atmosphere. Reproduced with permission from [112] Copyright 2021, The Authors, published by Springer Nature. **k** Phase diagram of NaCl/ZnCl_2_ mixture. Reproduced with permission from [113] Copyright 2021, Wiley–VCH

The mechanism of Lewis acidic molten salts etching method is based on the replacement reaction between Lewis acidic molten salts and A-site atoms in MAX precursor. Taking the etching of Ti_3_AlC_2_ MAX precursor by the firstly used Lewis acidic salt ZnCl_2_ as an example, Huang et al. thoroughly mixed the Ti_3_AlC_2_ and ZnCl_2_ with a molar ratio of 1:1.5 in a glovebox and the mixture was placed in alumina crucible, which is then heated at 550 °C for 5 h in a tube furnace under Ar atmosphere (Fig. 6a) [66]. It can be observed that main peaks of final product shift to lower angles relative to those of Ti_3_AlC_2_ MAX in the X-ray diffraction (XRD) patterns (Fig. 6b), revealing an enlarged lattice parameter. Additionally, the densely-packed structure can still be kept, and high-angle annular dark-field scanning transmission electron microscopy (HAADF-STEM) image in Fig. 6c suggests that interlayer element is brighter compared with other elements, thus proving that Ti_3_ZnC_2_ MAX has been successfully prepared. The specific formation mechanism can be described by Eqs. (15–16):

15 $${\text{Ti}}_{{3}} {\text{AlC}}_{{2}} + {1}.{\text{5ZnCl}}_{{2}} = {\text{Ti}}_{{3}} {\text{C}}_{{2}} + {1}.{\text{5Zn}} + {\text{AlCl}}_{{3}} \uparrow$$

16 $${\text{Ti}}_{{3}} {\text{C}}_{{2}} + {\text{Zn}} = {\text{Ti}}_{{3}} {\text{ZnC}}_{{2}}$$

It is well-known that ZnCl_2_ will exist in the form of molten state at 550 °C and is ionized into Zn^2+^ cations. Then, the Zn^2+^ with strong electron obtaining ability will react with Al atoms in Ti_3_AlC_2_ to generate Al^3+^ cations and Zn atoms. Al^3+^ cations are prone to combine with Cl^−^ anions to form AlCl_3_, which will evaporate at 178 °C. At the same time, Zn atoms can diffuse into the interlayer of Ti_3_C_2_ and occupy the position originally belonging to Al atoms, thereby leading to the generation of new MAX phase Ti_3_ZnC_2_. It is worth mentioning that the removal of AlCl_3_ from the system considerably accelerates the element replacement reaction.

When the molar ratio of Ti_3_AlC_2_ and ZnCl_2_ further increases to 1:6, Ti_3_C_2_ MXene with -Cl termination instead of Ti_3_ZnC_2_ MAX can be successfully obtained. Firstly, the classical multi-layered structure is observed for Ti_3_C_2_Cl_2_ MXene (Fig. 6d). Then, the XRD pattern of as reacted product indicates that the strongest (104) peak at around 39° of Ti_3_AlC_2_ MAX almost vanishes and the (002) peak moves to a lower angle of 7.94° (Fig. 6e), suggesting an expanded interlayer spacing. Additionally, the characteristic XRD peaks of Zn metal can also be observed. Further, Ti-Cl chemical bond in Ti_3_C_2_Cl_2_ MXene can be confirmed by Cl 2*p* X-ray photoelectron spectroscopy (XPS) spectra (Fig. 6f) [66]. Above discussions effectively confirm the formation of Ti_3_C_2_Cl_2_ MXene and the reaction process can be described by following equations:

17 $${\text{Ti}}_{{3}} {\text{ZnC}}_{{2}} + {\text{Zn}}^{{{2} + }} = {\text{Ti}}_{{3}} {\text{C}}_{{2}} + {\text{Zn}}_{{2}}^{{{2} + }}$$

18 $${\text{Ti}}_{{3}} {\text{C}}_{{2}} + {\text{2Cl}}^{ - } = {\text{Ti}}_{{3}} {\text{C}}_{{2}} {\text{Cl}}_{{2}} + {\text{2e}}^{ - }$$

19 $${\text{Zn}}_{{2}}^{{{2} + }} + {\text{2e}}^{ - } = {\text{2Zn}}$$

As exhibited in Fig. 6a, the formation of Ti_3_C_2_Cl_2_ MXene can be divided into two stages including the initial formation of Ti_3_ZnC_2_ MAX shown in Eqs. (15–16) and the subsequent reaction of Ti_3_ZnC_2_ MAX with excessive ZnCl_2_ to obtain Ti_3_C_2_Cl_2_ exhibited in Eqs. (17–19). Specifically, the Zn atoms in Ti_3_ZnC_2_ MAX can be separated from interlayer and react with excessive Zn^2+^ to generate Zn_2_^2+^ cations, and the Cl^−^ anions can easily intercalate into the interlayer of Ti_3_C_2_, forming Ti–Cl chemical bond (Fig. 6g). Meanwhile, the Zn_2_^2+^ cations gain electron to form Zn metal. In general, Zn^2+^ cations and Cl^−^ anions in ZnCl_2_ act as H^+^ and F^−^ in HF etchant, respectively.

Later in 2020, Prof. Huang and coworkers generalized the previously proposed Lewis acidic etching method, which greatly expands the family of Lewis acidic molten salts (FeCl_2_, CoCl_2_, NiCl_2_, CuCl_2_, CdCl_2_, CuI, CuBr_2_, etc.) and can prepare various MXenes from Al, Si and Ga-based MAX precursors [67]. They immersed Ti_3_SiC_2_ MAX in CuCl_2_/NaCl/KCl mixed molten salts at 750 °C (Fig. 6h), and NaCl and KCl play a role in providing molten salts environment. The etching process is illustrated as follows:

20 $${\text{Ti}}_{{3}} {\text{SiC}}_{{2}} + {\text{2CuCl}}_{{2}} = {\text{Ti}}_{{3}} {\text{C}}_{{2}} + {\text{ SiCl}}_{{4}} \uparrow + {\text{ 2Cu}}$$

21 $${\text{Ti}}_{{3}} {\text{C}}_{{2}} + {\text{CuCl}}_{{2}} = {\text{Ti}}_{{3}} {\text{C}}_{{2}} {\text{Cl}}_{{2}} + {\text{Cu}}$$

Specifically, the zero-valence Si atoms in the interlayer lose electrons to become Si^4+^ cations and then combine with Cl^−^ anions to form SiCl_4_, which can readily escape as gas at around 57 °C. Subsequently, the Cu^2+^ cations can obtain electrons to transform into Cu metal. Finally, excessive Cl^−^ anions can be grafted onto exposed Ti atoms, resulting in the formation of Ti_3_C_2_Cl_2_ MXene. It is worth mentioning that individual HF is difficult to etch Ti_3_SiC_2_ [85], while Lewis acidic salts can readily complete the above etching process, which reflects the superiority of Lewis acidic etching. After the etching process, NaCl, KCl and excessive CuCl_2_ can be eliminated by washing the etching products with deionized water.

Further, Huang et al. proposed an effective criterion to determine whether the A-site atoms in MAX can react with metal ions in the Lewis acidic salts. They found that the metal ions in the Lewis acidic salts with high electrochemical redox potential can etch the MAX precursor with low redox potential A-site atoms. The relationship between redox potential of A-site elements, redox potential of Lewis acidic salts and Gibbs free energy is illustrated in Fig. 6i. For example, CuCl_2_ molten salt can easily remove the Si atoms from Ti_3_SiC_2_ MAX as mentioned above, which is ascribed to the higher redox potential of Cu^2+^/Cu pair (−0.43 V versus Cl_2_/Cl^−^) compared with that of Si^4+^/Si pair (−1.38 V versus Cl_2_/Cl^−^). The general etching reaction between A-site elements in MAX phase and Lewis acidic molten salts MQ_*y*_ (Q = Cl, Br or I) can be described as follows:

22 $${\text{A}} + z/y{\text{MQ}}_{y} = {\text{AQ}}_{z} + z/y{\text{M}}$$

It can be inferred that the difficulty degree of etching M_*n*+1_AX_*n*_ precursors by Lewis acidic molten salts is independent of M, X and *n* values, and only depends on the redox potential of A-site element. Therefore, based on Lewis acidic etching criterion, a large number of MAX phases (Ti_3_AlC_2_, Ti_3_ZnC_2_, Ti_3_SiC_2_, Ti_3_AlCN, Ti_2_GaC, Ti_2_AlC, Ta_2_AlC, Nb_2_AlC, etc.) can be etched by using different Lewis acidic salts (FeCl_2_, CoCl_2_, NiCl_2_, CuCl_2_, CdCl_2_, AgCl, CuI, CuBr_2_, etc.).

The reaction mechanism of Lewis acidic etching method is basically clear now, but the understanding of Lewis acidic etching method can still be further deepened by optimizing etching parameters such as reaction atmosphere and etchant compositions. For example, in order to prevent the oxidation of the prepared MXenes, the inert gas such as Ar is generally used for protection in the process of Lewis acidic etching. However, Lin et al. proposed a simple one-pot synthesis method, containing molten salts shielded strategy for MAX preparation and subsequent in-situ Lewis acidic etching, to achieve the preparation of MXenes under air atmosphere (Fig. 6j) [112]. Specifically, they thoroughly mixed Ti, Al and C powders with NaCl and KCl salts, and the mixture was then pressed into a pellet. Subsequently, the pellet was further placed in a salt bed composed of NaCl and KCl which can melt at around 650 °C to insulate the air, and the Ti_3_AlC_2_ MAX can be obtained by heating at 1300 °C for 1 h in muffle furnace without Ar protection. Finally, CuCl_2_ was introduced to the reaction system at 700 °C to achieve the in-situ etching of Ti_3_AlC_2_ MAX for 10 min, thus leading to the quick formation of Cl-terminated Ti_3_C_2_T_*x*_ MXene. Therefore, with the assistance of the molten salts shielding strategy, Lewis acidic molten salts can directly etch MAX precursor in air atmosphere, which greatly promotes the development of Lewis acidic etching method. In addition, Zhang et al. demonstrated one-step eutectic etching strategy for the synthesis of in-plane porous Ti_3_C_2_Cl_2_ MXene by regulating the etchant composition from ZnCl_2_ to NaCl/ZnCl_2_ mixture [113]. When ZnCl_2_ is used to etch the MAX phase, the volatilization of generated AlCl_3_ at high temperature will cause structural/crystal defects in the precursor, which will then evolve into void space. However, the generated pores will gradually collapse owing to the absence of temporary physical support during cooling process. Further, they found that when the etchant is changed into a mixture of ZnCl_2_ and NaCl in a molar ratio of 4:6, the NaCl mole percent will gradually increase to 72% with the progress of etching process, which makes the salt melt shift across the phase boundary and leads to the precipitation of NaCl particles at 550 °C based on the phase diagram (Fig. 6k). The formation of NaCl particles can occupy void space to prevent the shrinkage and collapse of pores, finally resulting in a large increase in mesoporous volume and specific surface area. Therefore, the pore structure of the prepared MXenes can be greatly adjusted by the manipulation of etchant composition. The above-mentioned two works indicate that the Lewis acidic etching method still possesses broad development space and can be further improved from different aspects in the future.

### Terminations Regulation

A large number of theoretical calculations and experiments have proved that the composition of surface groups can considerably influence the physical and chemical properties of MXenes, such as electronic property, mechanical property, magnetic property, optical property, chemical stability, dispersibility and redox activity [15, 26, 55, 61, 63, 114–116]. Therefore, in order to accurately explore that how surface functional groups affect the properties of MXenes, it is pretty important to synthesize MXenes with controllable surface chemistry. However, preparing MXenes with single and uniform surface groups has been a huge challenge for researchers. Taking HF etching as an example, MXenes exhibits mixed surface terminations including –O, –OH and –F [31, 43]. Firstly, the complex and uncontrollable surface termination greatly affect the properties of MXenes, which is not conducive to the management of experimental variables and the large-scale preparation of MXenes. Additionally, functional groups like –F can irreversibly react with Li^+^ or Na^+^ cations and block their transport, which negatively affects the electrochemical performance of MXenes electrodes [117, 118]. Therefore, preparing MXenes with fluorine-free surface groups should be the best choice for researchers in the fields of lithium-ion batteries (LIBs) or sodium-ion batteries (SIBs). However, it is difficult to obtain MXenes with homogeneous and fluorine-free terminations before 2019. Fortunately, the fabrication of MXenes with totally controllable surface chemistry is realized by the propose of Lewis acidic molten salts etching strategy. The functional groups of MXenes prepared by Lewis acidic etching mainly depends on the Lewis acidic salts anions. For example, Ti_3_C_2_ MXene terminated by –Cl functional group can be synthesized by Cl^−^ anions-containing Lewis acidic salts etching. However, a small part of -Cl terminations may be replaced by –O groups during the washing process, and the content of –O surface group mainly depends on the competition between –O and –Cl terminations [67, 119]. Lin et al. proved that -O surface group in Nb_2_C MXene prepared by CuCl_2_ etching demonstrates stronger competitiveness compared with –Cl surface groups, thus leading to Nb_2_CT_*x*_ MXene rich in –O termination, while the competition between –O and –Cl for Ti_3_C_2_ MXene is relatively weak [119]. Since the –O functional groups are conducive to the electrochemical process of metal-ion batteries and SCs [120, 121], it is necessary to further increase the content of –O termination in Ti_3_C_2_T_*x*_ MXene. Pang et al. etched Ti_3_AlC_2_ by CuCl_2_·2H_2_O or CoCl_2_·6H_2_O Lewis acidic salts [122], the additional introduction of six water molecules can effectively raise the number of –O functional groups and the etching process can be described as follows:

23 $${\text{2Ti}}_{{3}} {\text{AlC}}_{{2}} + {\text{3CuCl}}_{{2}} \to {\text{2Ti}}_{{3}} {\text{C}}_{{2}} + {\text{2AlCl}}_{{3}} \uparrow + {\text{3Cu}}$$

24 $${\text{Ti}}_{{3}} {\text{C}}_{{2}} + {\text{CuCl}}_{{2}} \to {\text{Ti}}_{{3}} {\text{C}}_{{2}} {\text{Cl}}_{{2}} + {\text{Cu}}$$

25 $${\text{Ti}}_{{3}} {\text{C}}_{{2}} + {\text{2H}}_{{2}} {\text{O}} \to {\text{Ti}}_{{3}} {\text{C}}_{{2}} \left( {{\text{OH}}} \right)_{{2}} + {\text{H}}_{{2}}$$

26 $${\text{Ti}}_{{3}} {\text{C}}_{{2}} \left( {{\text{OH}}} \right)_{{2}} \to {\text{Ti}}_{{3}} {\text{C}}_{{2}} {\text{O}}_{{2}} + {\text{H}}_{{2}}$$

**Fig. 7:**
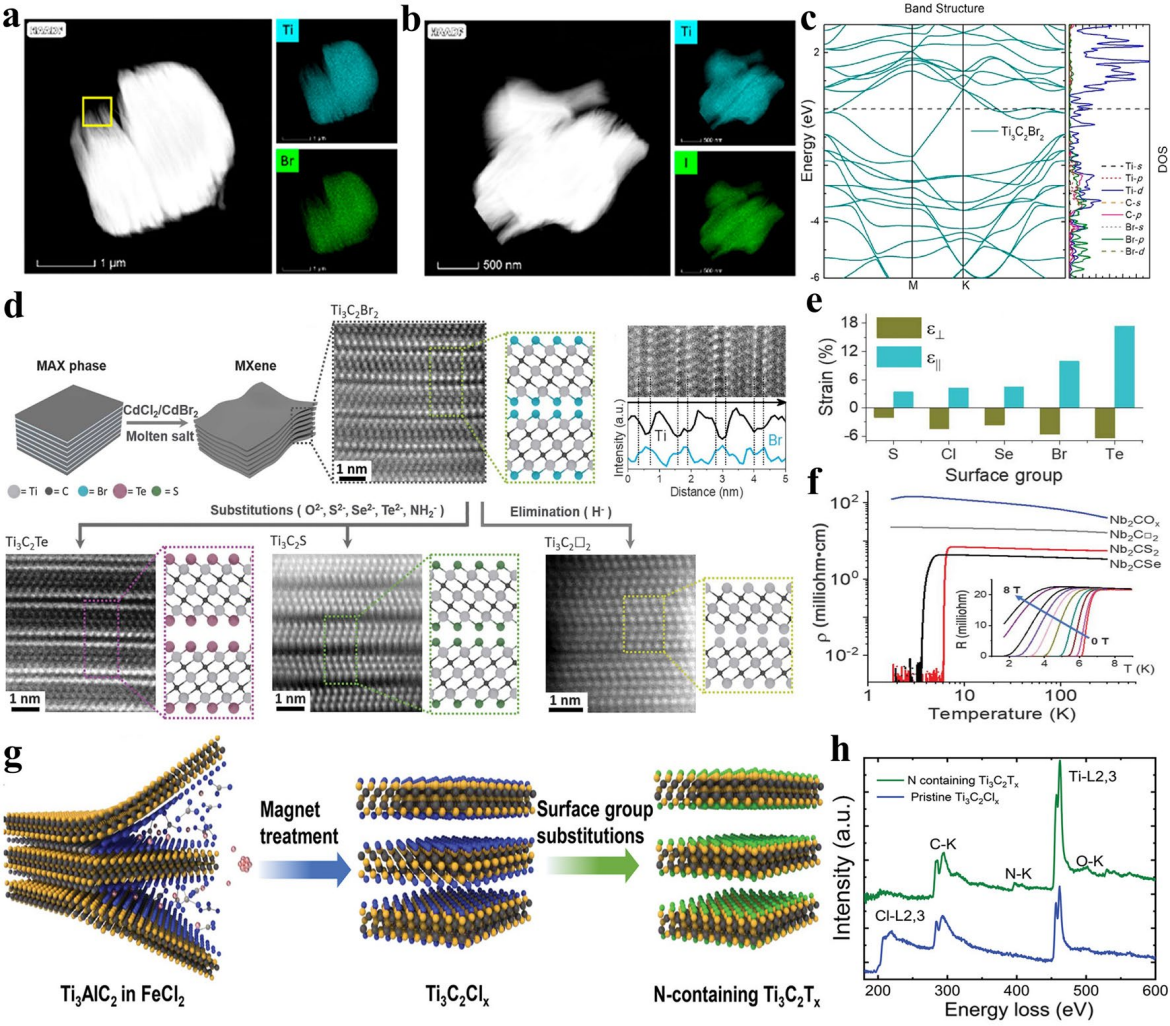
**a**–**b** HAADF-STEM images and corresponding EDS mapping of Ti_3_C_2_Br_2_ and Ti_3_C_2_I_2_ MXene, respectively. **c** Electronic structure of Ti_3_C_2_Br_2_ MXene. Reproduced with permission from [123] Copyright 2021, American Chemical Society. **d** Schematic showing the preparation of Ti_3_C_2_T_*x*_ MXene via Lewis acidic etching and subsequent termination substitution in molten inorganic salts as well as corresponding HAADF-STEM images and EDS analysis. **e** Biaxial straining of Ti_3_C_2_T_*x*_ MXene lattice induced by the terminations. **f** Temperature-dependent resistivity of Nb_2_CT_*x*_ MXenes. Reproduced with permission from [42] Copyright 2020, The American Association for the Advancement of Science. **g** Schematic of the preparation of N-containing Ti_3_C_2_T_*x*_ MXene via FeCl_2_ Lewis acidic salt etching. **h** EELS analysis of pristine Ti_3_C_2_T_*x*_ and N-containing Ti_3_C_2_T_*x*_ MXene. Reproduced with permission from [126] Copyright 2022, The Authors, published by Wiley–VCH

Due to the wide variety of halogen elements in the Lewis acidic molten salts, MXene can also be terminated by other halogen anions. For example, Ti_3_C_2_ MXene with –Br or –I functional groups can be obtained via CuBr_2_ or CuI etching, respectively (Fig. 7a-b). Further, Ti_3_C_2_ MXene can be endowed with –BrI, –ClI and –ClBr binary terminals as well as –ClBrI ternary halogen terminations. It is gratifying to note that Ti_3_C_2_ MXene with –Cl, –Br or –I functional groups all exhibit metallic-level conductivity (Fig. 7c), while F or OH-terminated Ti_3_C_2_ MXene often demonstrates semiconductor behavior [11, 66, 123, 124]. More importantly, due to the strong electrochemical reactivity, Br or I-terminated Ti_3_C_2_ MXene demonstrates excellent zinc storage performance. However, Ti_3_C_2_ MXene with –O, –F, –OH and –Cl functional groups exhibit low discharge specific capacity and oblique charge discharge curve when used as cathode for zinc-ion batteries (ZIBs) [123]. The above discussion reveals that the electrochemical performance of MXene electrodes can be greatly enhanced by adjusting the type of functional groups. Additionally, the halogen terminations of MXenes can be used as halogen sources and nucleation sites to prepare MXene/halide heterostructures. For example, Cui et al. synthesized intimate contact Bi_12_O_17_Cl_2_/Ti_3_C_2_ MXene 2D/2D heterojunctions by the in-situ growth of Bi_12_O_17_Cl_2_ nanosheets on the surface of Cl-terminated Ti_3_C_2_ MXene that was obtained via CuCl_2_ etching [125].

Since the bond dissociation energies of Ti–Br and Ti–Cl bond are smaller than that of Ti–O, Ti–OH and Ti–F bonds [126], it is possible to chemically modify the surface of MXenes originally terminated with –Br or –Cl functional groups at a relatively low temperature. Based on this point, Kamysbayev et al. proposed a universal strategy to successfully prepare O, S, Se, Te and NH-terminated Ti_3_C_2_ MXene and non-terminated Ti_3_C_2_ via Lewis acidic etching and subsequent termination substitution/elimination reactions in molten inorganic salts (Fig. 7d) [42]. Firstly, Ti_3_C_2_Br_2_ MXene was prepared by mixing Ti_3_AlC_2_ MAX with CdBr_2_ Lewis acidic salt. As followed, Ti_3_C_2_Br_2_ MXene was dispersed in CsBr/KBr/LiBr eutectic salt at 300 °C and then reacted with NaNH_2_, Li_2_O, Li_2_S, Li_2_Se or Li_2_Te at around 500–600 °C in an Ar-filled glove box to synthesize Ti_3_C_2_(NH), Ti_3_C_2_O, Ti_3_C_2_S, Ti_3_C_2_Se, Ti_3_C_2_Te MXene, respectively. Moreover, when treated with LiH at 300 °C, the surface groups of Ti_3_C_2_Br_2_ MXene can be completely eliminated, thus leading to the formation of bare Ti_3_C_2_ MXene. This strategy realizes the controllable chemical transformation of functional groups for the first time. The surface groups can significantly regulate the interatomic spacings of MXenes lattice and their electronic properties. For example, the Ti_3_C_2_Te lattice exhibits a greater in-plane lattice expansion (18%) (Fig. 7e). More importantly, Cl, S and Se-terminated Nb_2_C MXene prepared by the above-mentioned strategy exhibits unprecedented superconductivity behavior (Fig. 7f), while Nb_2_C MXene with –F and –O terminations synthesized by HF etching fails to demonstrate superconducting state, which is also proved by theoretical calculations [62]. The discovery of superconductivity phenomenon is mainly attributed to the influence of functional groups including –Cl, –S and -Se on MXenes structure, such as lattice strain, carrier localization, phonon frequency, and electron–phonon coupling [127], which greatly promotes the research of MXenes in the field of physics.

Very recently, Zhang et al. reported the synthesis of Ti_3_C_2_Br_*x*_ MXene through CuCl_2_ etching of Ti_3_AlC_2_ precursor and subsequent termination substitution in AlBr_3_/NaBr/KBr eutectic molten salts [128]. When the molar ratio of AlBr_3_ is lower than 50%, the AlBr_3_/NaBr/KBr molten system with plenty of naked Na^+^, K^+^ and Br^−^ demonstrates strong Lewis basicity. The Na^+^ and K^+^ cations can intercalate into the interlayer of MXene and the previous –Cl termination can be replaced by the Br^−^ anions via nucleophilic reaction, which simultaneously achieves the increase of interlayer spacing and the substitution of surface groups. The enlarged interlayer spacing leads to an expanded multi-layered morphology of Ti_3_C_2_Br_*x*_ MXene, which may be conducive to the following delamination process to obtain few-layered nanosheets. In addition, Liu et al. synthesized N-containing Ti_3_C_2_T_*x*_ MXene via FeCl_2_ Lewis acidic etching, subsequent magnet cleaning and final thermal treatment in Li_3_N/LiCl/KCl molten salts (Fig. 7g) [126]. The magnet cleaning process can not only effectively remove Fe nanoparticles, but also prevent the –Cl surface group from being largely replaced by –O, which is beneficial for following termination substitution reaction. The dramatic decrease in Cl L2,3-edge intensity and appearance of N–K edge in electron energy-loss spectroscopy (EELS) confirm that the -Cl functional groups can be transformed to –N terminations in Li_3_N/LiCl/KCl Lewis basic molten salts (Fig. 7h), thus leading to the N-containing Ti_3_C_2_T_*x*_ MXene.

In short, combined with Lewis acidic etching, termination exchange reactions in Lewis basic molten salts medium can endow MXenes with unconventional terminations, which have never appeared before and are hard to be directly obtained by individual Lewis acidic etching. By selecting various Lewis bases with different anions such as S^2−^, Se^2−^, Te^2−^, N^3−^ and NH_2_^−^, researchers can obtain the desired surface groups-terminated MXenes. The above discussions fully reveal the great potential of the halogenated MXenes synthesized by Lewis acidic etching method in various fields. Taking consideration of the rich selection of M and X elements as well as various surface groups mentioned above, there are a large number of new MXenes that can be explored, which may show some excellent properties. Up to now, the regulation of MXene surface groups through chemical transformation is still in its infancy and plenty of theoretical calculations and experiments are required to further explore the effect of these new functional groups on the properties of different MXenes, such as electrical conductivity, ion diffusion kinetics, thermal conductivity, work function, optical conductivity, mechanical property, hydrophilicity, chemical and thermal stability.

### In-Situ Formed Metals

**Fig. 8:**
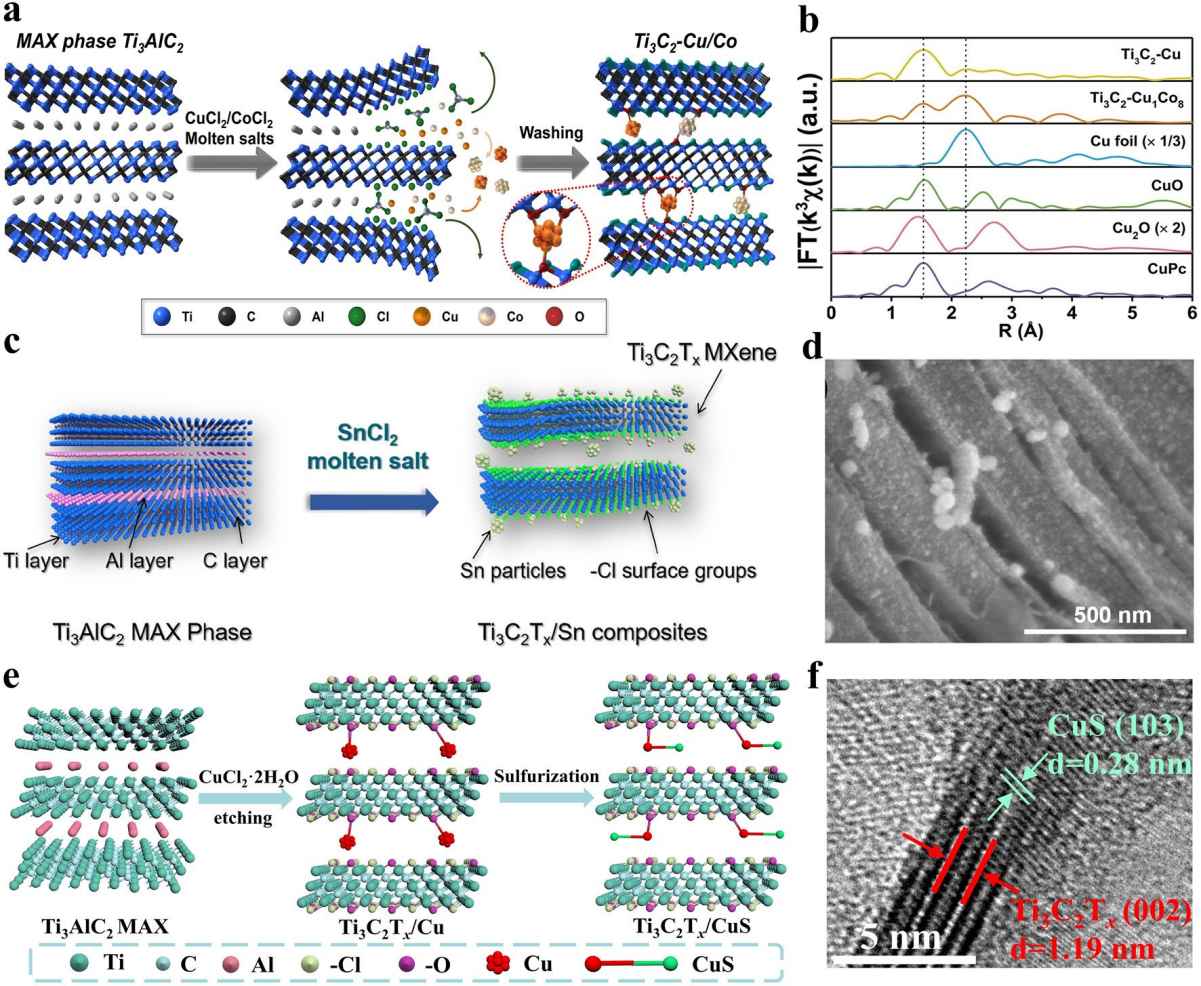
**a** Schematic illustration of the synthesis of Ti_3_C_2_-Cu/Co composites. **b** XAS spectra of Ti_3_C_2_-Cu hybrids. Reproduced with permission from [122] Copyright 2021, Wiley–VCH. **c** Schematic showing the preparation of Ti_3_C_2_T_*x*_/Sn composites by SnCl_2_ Lewis acidic salt etching. **d** SEM image of Ti_3_C_2_T_*x*_/Sn hybrids. Reproduced with permission from [145] Copyright 2022, Elsevier. **e** Schematic diagram of the preparation of Ti_3_C_2_T_*x*_/CuS composites. **f** High-resolution transmission electron microscope (HRTEM) image of Ti_3_C_2_T_*x*_/CuS. Reproduced with permission from [148] Copyright 2022, Royal Society of Chemistry

According to the etching mechanism shown in Eq. (22), metal ions in the Lewis acidic molten salts can be in-situ reduced to various metals during the etching process of MAX precursors, thus leading to the formation of MXenes/metals composites. Taking CuCl_2_ etching of Ti_3_SiC_2_ MAX as an example, Cu nanoparticles can be uniformly anchored among the multi-layered Ti_3_C_2_T_*x*_ MXene matrix and the Ti_3_C_2_T_*x*_/Cu hybrids can be successfully obtained [67]. It is worth mentioning that Cu nanoparticles are able to be loaded in the Ti_3_C_2_T_*x*_ MXene matrix through one-step in-situ growth process, so the interaction between them is stronger than that of Ti_3_C_2_T_*x*_/Cu composites prepared by mechanical mixing, which is another key advantage of Lewis acidic etching method relative to traditional etching route. Recently, it has been reported that when the Ti_3_AlC_2_ MAX precursor is etched by CuCl_2_·2H_2_O or CoCl_2_·6H_2_O, the obtained Ti_3_C_2_T_*x*_ MXene can be decorated with Cu or Co nanoparticles (Fig. 8a), respectively [122]. The strong interaction between Cu nanoparticles and –O surface group of Ti_3_C_2_T_*x*_ MXene can be proved by X-ray absorption (XAS) spectrum (Fig. 8b), effectively preventing the detachment of Cu from the Ti_3_C_2_T_*x*_ MXene matrix. Furthermore, owing to the diversity of MXene compositions and the large family of Lewis acidic molten salts, various metal nanoparticles (Fe, Co, Ni, Zn, Ag, Cd, Sn, etc.) can be distributed among the different MXenes (Ti_3_C_2_, Ti_3_CN, Ti_2_C, Ta_2_C, Nb_2_C, etc.), which greatly enriches the choice of researchers.

In the early development stage of the Lewis acidic etching strategy, researchers generally remove the in-situ generated metal nanoparticles from MXenes/metals composites in order to obtain pure MXenes, which almost becomes a custom. However, metal or metal compounds have been intensively investigated in energy storage and conversion, electromagnetic interference shielding and other fields in the past few decades [129–133]. Consequently, rational and effective application of various metals in MXenes/metals composites obtained by Lewis acidic etching is worth investigating. Additionally, MXenes have been widely utilized to support metal or metal compounds. Based on the excellent synergistic effect between MXenes and metals or metal compounds, MXenes/metals hybrids and MXenes/metal compounds composites exhibit better performance than their individual components and have been widely investigated in various fields [54, 73, 134–138]. Nevertheless, the traditional fabrication process of MXenes/metals hybrids or MXenes/metal compounds composites usually requires the synthesis of MXenes and the subsequent introduction of additional metal sources, which makes the preparation process complicated [117, 139–144]. As mentioned above, Lewis acidic salts can achieve the etching of MAX precursor and the introduction of metal sources simultaneously. Therefore, if Lewis acidic molten salts are used to etch MAX phases, the obtained MXenes/metals composites can be applied directly, and then the MXenes/metal compounds composites can be prepared by one-step sulfurization, phosphorization, tellurization or selenization treatment of MXenes/metals composites, which opens a new window for the universal construction of MXene-based hybrids through Lewis acidic etching route, showing great research potential.

In general, MXenes/metals or MXenes/metal compounds composites can be obtained quickly and easily by taking full advantage of Lewis acidic etching products. As expected, the MXenes/metals and MXenes/metal compounds composites prepared by Lewis acidic etching route have been rationally applied in some fields due to the superior structural stability. For example, the reported Ti_3_C_2_–Cu composites via CuCl_2_·2H_2_O molten salts etching exhibit superior electrochemical performance in SCs when used as electrode material [122]. Additionally, Sn-nanoconfined Ti_3_C_2_T_*x*_ MXene was synthesized by one-step SnCl_2_ molten salt etching (Fig. 8c–d), and demonstrated excellent cyclic stability as anode for LIBs [145]. Further, CoCl_2_ etching and in-situ sulfidation process were used to prepare strongly coupled N-doped MXene-CoS_2_ composites, which can effectively alleviate the shuttle effect of lithium polysulfides (LiPSs) in lithium-sulfur (Li–S) batteries [146]. Recently, our group presented a general method to fabricate a series of Ti_3_C_2_T_*x*_ MXene/transition metal sulfides (MS_*y*_, M = Fe, Co, Ni or Cu) composites via Lewis acidic etching and subsequent sulfurization treatment (Fig. 8e-f) [147, 148]. When served as anodes for SIBs, the readily produced hybrids can show greatly boosted sodium storage performance owing to the interfacial electronic coupling. Until now, the applications of Lewis acidic molten salts etching products, especially the in-situ formed metals, have been relatively rare and concentrated in the field of energy storage. More rational utilization of etching products in other fields, such as electromagnetic interference shielding, demonstrates great potential and deserves further exploration.

### Delamination of Multi-Layered MXenes

The MXenes prepared by Lewis acidic etching strategy exhibit obvious multi-layered accordion morphology. In order to demonstrate true 2D morphology, it is necessary to implement intercalation and delamination process to obtain single-layered or few-layered MXene nanosheets, which can expose more active sites and possess higher surface area [71]. In general, MXenes prepared by HF etching are terminated with –O and –OH functional groups and therefore show excellent hydrophilicity [81], which makes it easy to disperse MXenes in water. The well-dispersed MXenes can then be intercalated by organic molecules (urea, amine, DMSO, TMAOH, TBAOH, IPA, etc.) and inorganic substances (LiCl, NaCl, KCl), which increases the gallery spacing and weakens the van der Waals force and hydrogen bond between MXene layers [43, 78–80, 149, 150]. Finally, the few-layered MXenes nanosheets can be obtained by ultrasonic and centrifugation processing [151, 152]. Nevertheless, MXenes prepared by Lewis acidic etching method fail to be terminated by –OH functional group, which greatly decreases their hydrophilicity [153–155]. Therefore, it is difficult to uniformly disperse the obtained MXenes in water to form stable colloidal solution, which greatly reduces the delamination efficiency, thus exhibiting a low yield of few-layered and single-layered nanosheets. In order to enhance the delamination efficiency, researchers often considerably extend the sonication time, which tends to endow MXenes with small lateral size and poor quality. In general, most of the MXenes synthesized by Lewis acidic etching is at the multi-layered state according to the published articles, and only a few of them are at the few-layered state.

For example, Ti_3_C_2_Cl_*x*_ nanosheets with lateral dimensions of 0.4–2.0 μm were prepared by 10 h ultrasonication treatment of multi-layered MXene obtained by ZnCl_2_ etching [156]. Yang et al. synthesized Ti_3_C_2_T_*x*_ nanosheets through ZnCl_2_ etching and IPA intercalation assisted by ultrasonication treatment for 20 h [157]. Additionally, Zhao et al. successfully produced Ti_3_C_2_Cl_*x*_ nanosheets via ZnCl_2_ etching and subsequent ultrasonication process lasting 20 h [158]. Further, Zhou et al. prepared Cl-terminated Ti_3_C_2_ MXene by CdCl_2_ molten salt etching, and the multi-layered MXene was intercalated by IPA molecule followed by 25 h ultrasonication process to obtain few-layered nanosheets with lateral size of hundreds of nanometers [159]. Furthermore, Liu et al. used large organic base molecule TBAOH to intercalate into Ti_3_C_2_T_*x*_ MXene produced by CuCl_2_ etching, followed by ultrasonication bath for 6 h at low temperature to separate the layers, leading to the few-layered nanosheets with a lateral size of around 600 nm [153]. It is worth mentioning that TMAOH intercalated Lewis acidic salts etched-Ti_3_C_2_T_*x*_ MXene suspension can be stably stored for 2 weeks without obvious precipitation. Finally, DMSO molecule intercalation and ultrasonication process can also be used to delaminate the Cl-terminated Ti_3_C_2_ MXene prepared by CuCl_2_ etching to obtain few-layered samples [125]. To sum up, due to the poor hydrophilicity of MXenes prepared by Lewis acidic etching, the delamination usually requires large organic molecules and long-time sonication, which leads to low yields of few-layered MXenes and small lateral sizes of nanosheets.

**Fig. 9:**
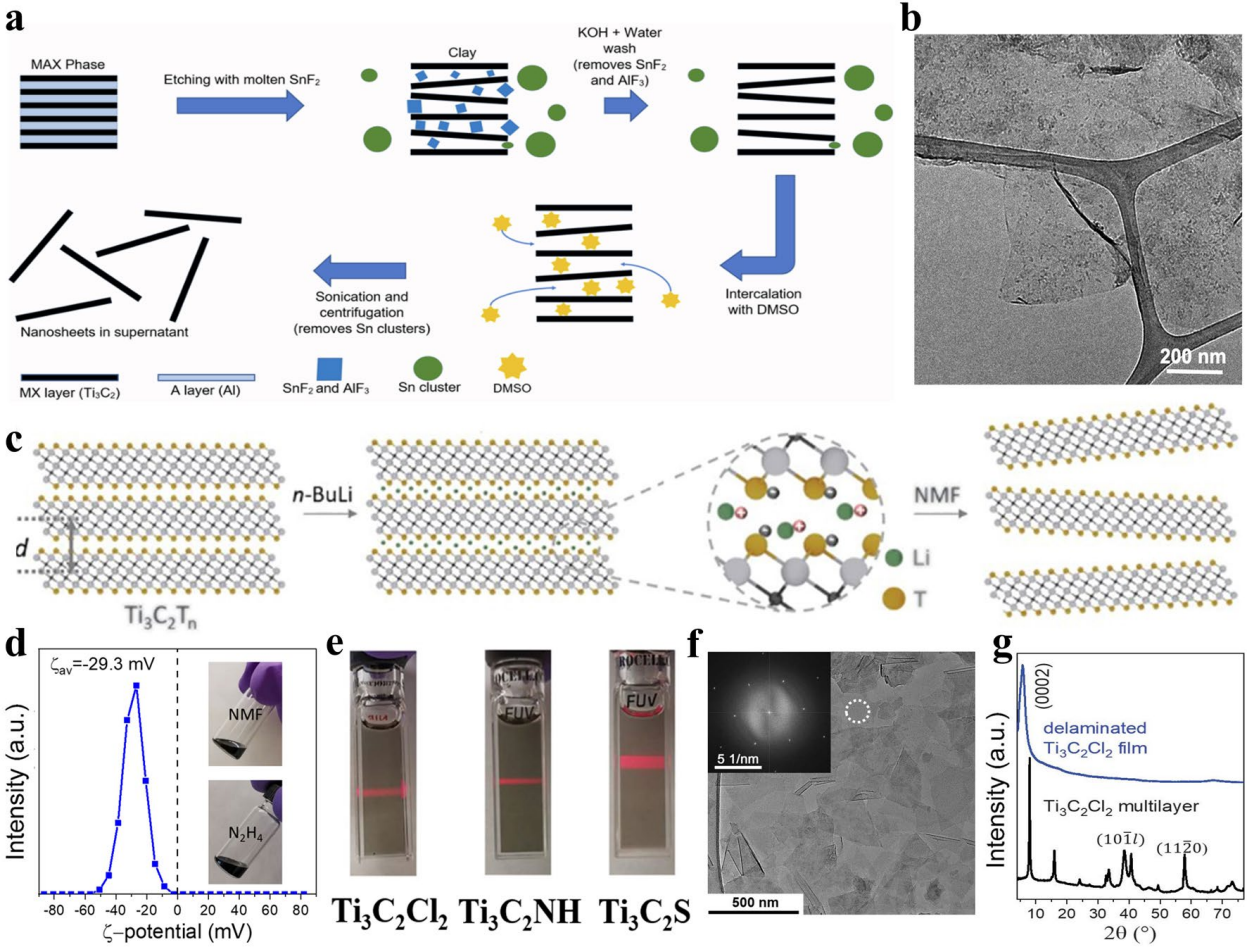
**a** Schematic of the synthesis of KOH-treated few-layered Ti_3_C_2_T_*x*_ MXene nanosheets. **b** Transmission electron microscope (TEM) image of Ti_3_C_2_T_*x*_ nanosheets. Reproduced with permission from [154] Copyright 2021, The Authors. **c** Schematic of the intercalation and delamination process. **d** Zeta potential of Ti_3_C_2_Cl_2_ MXene in NMF and inset representing the high concentrated MXene inks in NMF and N_2_H_4_. **e** Digital photographs of stable colloidal solutions of Ti_3_C_2_T_*x*_ MXenes (T = –Cl, –NH and –S) in NMF showing Tyndall effect. **f** TEM image of Ti_3_C_2_Cl_2_ MXene nanosheets and inset showing its selected area electron diffraction (SAED) pattern. **g** XRD patterns of multi-layered and delaminated Ti_3_C_2_Cl_2_ MXene. Reproduced with permission from [42] Copyright 2020, The American Association for the Advancement of Science

In order to solve the above-mentioned issues, two viable and effective methods have been proposed. The first method is to enhance the hydrophilicity of MXenes, and the other method is to disperse MXenes in organic solvents instead of water for intercalation and delamination. For example, Arole et al. presented a method to produce water-dispersible Ti_3_C_2_T_*x*_ nanosheets via SnF_2_ molten salt etching, KOH washing, DMSO intercalation and bath sonication for 1 h (Fig. 9a) [154]. The KOH washing facilitates the introduction of –OH terminal groups on Ti_3_C_2_T_*x*_ MXene and thus greatly boosts the formation of stable Ti_3_C_2_T_*x*_ suspension with a zeta potential of −31.7 mV. The few-layered or single-layered Ti_3_C_2_T_*x*_ nanosheets can be prepared by DMSO intercalation and sonication for only 1 h (Fig. 9b), which is conducive to reduce the required energy during the sonication process. Finally, the Ti_3_C_2_T_*x*_ film produced by vacuum filtration exhibits an electronic conductivity of 706 S cm^−1^, In addition, Kamysbayev et al. immersed multi-layered Ti_3_C_2_T_*x*_ (T = –Cl, –S or –NH) MXene obtained by Lewis acidic etching and surface functionalization in *n*-butyllithium (*n*-BuLi) hexanes solution to complete the Li^+^ intercalation process (Fig. 9c). Then, the lithium-intercalated Ti_3_C_2_T_*x*_ MXene was added to the polar organic solvent N-methyl formamide (NMF) rather than water, leading to the stable colloidal solution with a zeta potential of −29.3 mV after bath sonication for 1 h (Fig. 9d) [42]. The as-prepared Ti_3_C_2_T_*x*_ suspension of single-layered nanosheets exhibit obvious Tyndall effect and superior crystallinity (Fig. 9e–g), showing the advantage and potential of delamination in polar organic solvents. However, *n*-BuLi is difficult to be widely used as intercalator due to its high risk. In conclusion, although some progress has been made in the delamination of multi-layered MXenes obtained by Lewis acidic etching, there is still no simple, safe and effective method to prepare MXene nanosheets with high yield and large lateral size.

Table 1 compares non-Lewis acidic etching strategies with Lewis acidic etching in terms of some key indicators such as universality, scalability, safety and terminations.

**Table 1:** Comparison of non-Lewis acidic etching techniques and Lewis acidic etching routes

Methods	Universality	Scalability	Safety	Terminations
HF etching	Good	Good	Poor	–O, –OH and –F
In-situ HF etching	Good	Good	Medium	–O, –OH and –F
Alkali etching	Poor	Poor	Poor	–O and –OH
Electrochemical etching	Medium	Poor	Good	–O, –OH and –Cl
Bifluoride salts etching	Poor	Medium	Medium	–O, –OH and –F
Common molten salts etching	Poor	Medium	Good	–O and –F
Lewis acidic etching	Good	Good	Good	–Cl, –Br or –I

It can be concluded that Lewis acidic etching exhibits good universality, scalability and safety relative to other methods. Table 2 comprehensively summarizes the parameters for the preparation of various MXenes by Lewis acidic etching, which includes the molar ratio of MAX precursor and Lewis acidic salts, reaction temperature, time and atmosphere. Additionally, delamination parameters such as the type of intercalators and sonication time are also presented.

**Table 2:** The specific parameters for the preparation of various MXenes via Lewis acidic etching

MXenes	MAX: Lewis acidic salt (molar ratio)	Reaction condition	Intercalator	Sonication time	Refs.
Ti_3_C_2_Cl_2_	Ti_3_AlC_2_:ZnCl_2_ = 1:6	550 °C, 5 h, Ar	**/**	**/**	[66]
Ti_3_C_2_Cl_2_	Ti_3_AlC_2_:ZnCl_2_ = 1:6	550 °C, 7 h, Ar	**/**	**/**	[246]
Ti_3_C_2_Cl_2_	Ti_3_AlC_2_:ZnCl_2_ = 1:2.4	650 °C, 5 h, Ar	/	**/**	[211]
Ti_3_C_2_Cl_2_	Ti_3_AlC_2_:ZnCl_2_ = 1:6	550 °C, 7 h, Ar	/	**/**	[247]
Ti_3_C_2_Cl_2_	Ti_3_AlC_2_:ZnCl_2_ = 1:6	550 °C, 5 h, Ar	/	**/**	[113]
Ti_3_C_2_Cl_2_	Ti_3_AlC_2_:CuCl_2_ = 1:6	550 °C, 5 h, Ar	/	**/**	[237]
Ti_3_C_2_Cl_2_	Ti_3_AlC_2_:CuCl_2_ = 1:3	700 °C, 7 h, Ar	/	**/**	[212]
Ti_3_C_2_Cl_2_	Ti_3_AlC_2_:CuCl_2_ = 1:3	700 °C, 10 h, Ar	/	**/**	[218]
Ti_3_C_2_Cl_2_	Ti_3_AlC_2_:CdCl_2_ = 1:8	610 °C, 6 h, Ar	NaCl	72 h	[219]
Ti_3_C_2_Cl_2_	Ti_3_AlC_2_:CdCl_2_ = 1:8	610 °C, 6 h, Ar	CTAB	72 h	[219]
Ti_3_C_2_Cl_2_	Ti_3_AlC_2_:CdCl_2_ = 1:8	600 °C, 6 h, /	/	**/**	[222]
Ti_3_C_2_Cl_*x*_	Ti_3_AlC_2_:ZnCl_2_ = 1:5.7	550 °C, 10 h, Ar	**/**	10 h	[156]
Ti_3_C_2_Cl_*x*_	Ti_3_(Al_1-*x*_Cu_*x*_)C_2_:ZnCl_2_ = 1:7	600 °C, 5 h, Ar	**/**	20 h	[158]
Ti_3_C_2_Cl_*x*_	Ti_3_AlC_2_:CdCl_2_ = 1:8	610 °C, 7 h, N_2_	IPA	25 h	[159]
Ti_3_C_2_Cl_*x*_	Ti_3_AlC_2_:NiCl_2_ = 1:6	750 °C, 0.3 h, Air	/	**/**	[177]
Ti_3_C_2_Cl_*x*_	Ti_3_AlC_2_:CuCl_2_ = 1:3	700 °C, 7 h, Ar	DMSO	**/**	[125]
Ti_3_C_2_Cl_*x*_	Ti_3_AlC_2_:CuCl_2_ = 1:3	700 °C, 24 h, Ar	/	**/**	[128]
Ti_3_C_2_Cl_*x*_	Ti_3_AlC_2_:FeCl_2_ = 1:3	700 °C, 7 h, Ar	/	**/**	[126]
Ti_3_C_2_Br_2_	Ti_3_AlC_2_:CdBr_2_ = 1:8	610 °C, 6 h, Ar	*n*-BuLi	1 h	[42]
Ti_3_C_2_Br_*x*_	Ti_3_AlC_2_:NiBr_2_ = 1:6	750 °C, 0.3 h, Air	/	/	[178]
Ti_3_C_2_I_2_	Ti_3_AlC_2_:CuI = 1:6	700 °C, 7 h, Ar	**/**	**/**	[123]
Ti_3_C_2_I_2_	Ti_3_AlC_2_:CuI = 1:6	700 °C, 7 h, Ar	/	/	[194]
Ti_3_C_2_I_*x*_	Ti_3_AlC_2_:CuI = 1:6	700 °C, 7 h, Ar	/	**/**	[234]
Ti_3_C_2_T_*x*_	Ti_3_SiC_2_:CuCl_2_ = 1:3	750 °C, 24 h, Ar	**/**	**/**	[67]
Ti_3_C_2_T_*x*_	Ti_3_AlC_2_:CuCl_2_ = 1:3	680 °C, 24 h, Ar	TBAOH	6 h	[153]
Ti_3_C_2_T_*x*_	Ti_3_AlC_2_:CuCl_2_ = 1:6	700 °C, 0.7 h, Air	/	**/**	[175]
Ti_3_C_2_T_*x*_	Ti_3_AlC_2_:CuCl_2_ = 1:3	700 °C, 8 h, Ar	/	**/**	[180]
Ti_3_C_2_T_*x*_	Ti_3_AlC_2_:CuCl_2_·2H_2_O = 1:3	750 °C, 24 h, Ar	**/**	**/**	[122]
Ti_3_C_2_T_*x*_	Ti_3_AlC_2_:CuCl_2_·2H_2_O = 1:3	750 °C, 20 h, Ar	/	**/**	[148]
Ti_3_C_2_T_*x*_	Ti_3_AlC_2_:ZnCl_2_ = 1:8.8	600 °C, 5 h, Ar	IPA	20 h	[157]
Ti_3_C_2_T_*x*_	Ti_3_AlC_2_:CoCl_2_ = 1:3	750 °C, 15 h, Ar	/	**/**	[232]
Ti_3_C_2_T_*x*_	Ti_3_AlC_2_:CoCl_2_ = 1:3	700 °C, 24 h, Ar	/	**/**	[146]
Ti_3_C_2_T_*x*_	Ti_3_AlC_2_:CoCl_2_ = 1:3	750 °C, 2 h, N_2_	/	**/**	[251]
Ti_3_C_2_T_*x*_	Ti_3_AlC_2_:FeCl_2_ = 1:5	700 °C, 6 h, Ar	/	**/**	[244]
Ti_3_C_2_T_*x*_	Ti_3_AlC_2_:FeCl_2_·4H_2_O = 1:3	750 °C, 24 h, Ar	/	**/**	[147]
Ti_3_C_2_T_*x*_	Ti_3_AlC_2_:SnCl_2_ = 1:3	600 °C, 8 h, Ar	/	**/**	[145]
Ti_3_C_2_T_*x*_	Ti_3_AlC_2_:SnF_2_ = 1:6	550 °C, 6 h, Ar	DMSO	1 h	[154]
Ti_3_CNCl_2_	Ti_3_AlCN:CoCl_2_ = 1:6	750 °C, 24 h, Ar	**/**	1 h	[250]
Ti_3_CNCl_2_	Ti_3_AlCN:NiCl_2_ = 1:4.6	750 °C, 4 h, Ar	/	**/**	[233]
Ti_3_CNT_*x*_	Ti_3_AlCN:CuCl_2_ = 1:3	700 °C, 24 h, Ar	/	**/**	[240]
Ti_2_NT_*x*_	Ti_2_AlN:CuCl_2_ = 1:3	450 °C, 1 h, Ar	/	**/**	[176]
Nb_2_CCl_*x*_	Nb_2_AlC:CdCl_2_ = 1:10	750 °C, 36 h, Ar	/	**/**	[62]
Nb_2_CT_*x*_	Nb_2_AlC:CuCl_2_ = 1:3	750 °C, 5 h, Ar	**/**	**/**	[119]
Nb_2_CT_*x*_	Nb_2_AlC:SnF_2_ = 1:6	750 °C, 36 h, Ar	i-PrA	1 h	[155]

## Applications

Benefiting from the unique layered structure, superior electronic conductivity, low ion diffusion barrier, uniform and tunable surface functional groups and large interlayer spacing, the obtained MXenes and MXene-based composites via Lewis acidic molten salts etching route have been intensively investigated in various fields, such as energy storage, energy conversion, sensors and microwave absorption.

### Energy Storage

Due to the rapid consumption of fossil fuels and gradually serious environmental pollution, the development of clean renewable energy has become more and more important [160, 161]. However, the intermittency and instability of renewable energy makes it necessary to store the generated electricity [162]. In recent years, electrochemical energy storage devices have received increasing research interests because of their fast response speed, versatility and application flexibility [163]. Among the various electrochemical energy storage devices, LIBs currently occupy an absolute dominant position. Other technologies such as SIBs, ZIBs, Li–S batteries and SCs are in a rapid development period [164]. In particular, SIBs are promising for large-scale energy storage in the following few years owing to the low price and abundant sodium resources [165].

#### Lithium-Ion Batteries

**Fig. 10:**
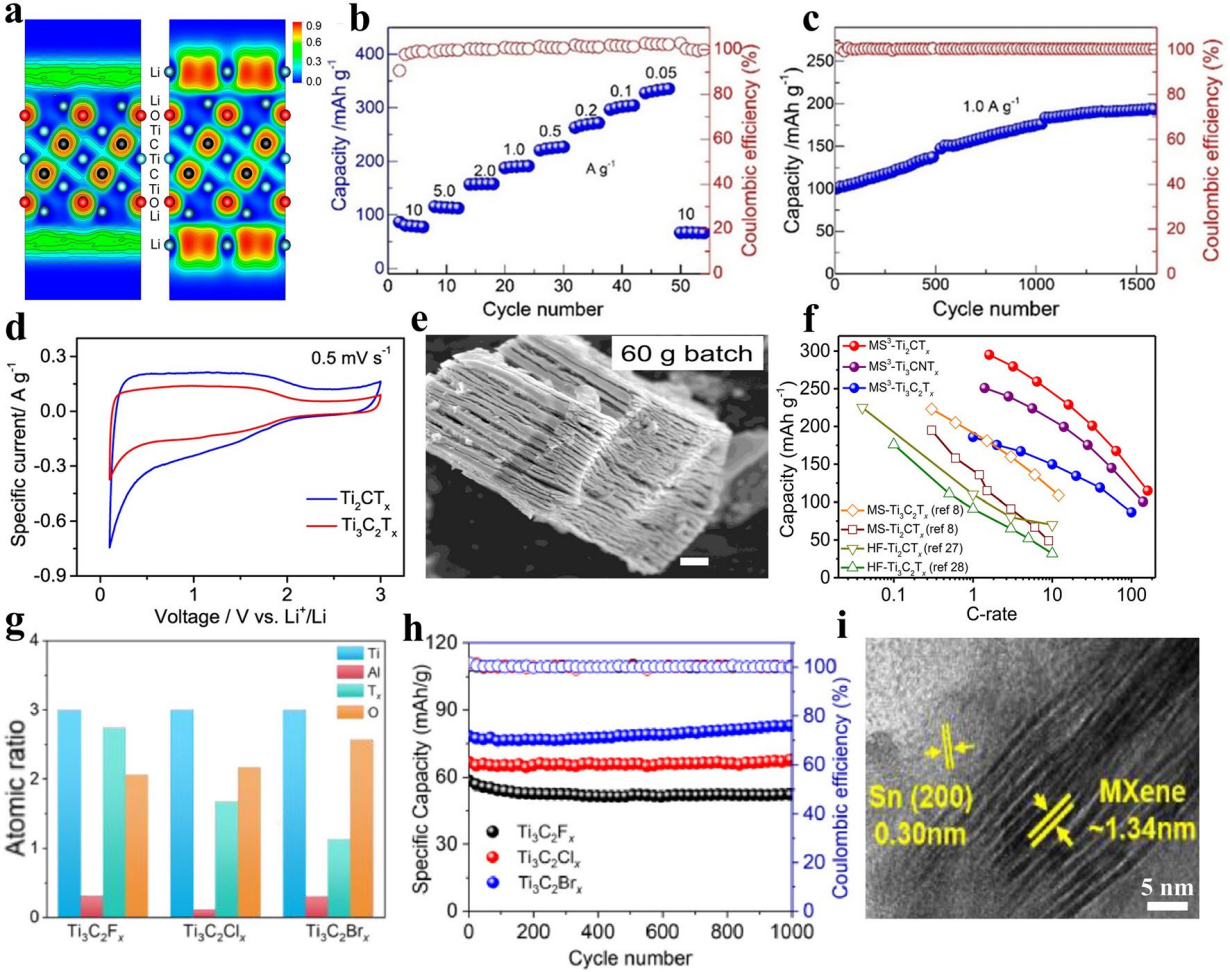
**a** Valence electron localization function of Ti_3_C_2_O_2_Li_2_ with or without an extra Li layer. Reproduced with permission from [120] Copyright 2014, American Chemical Society. **b** Rate performance of Nb_2_CT_*x*_ MXene. **c** Long-term cyclic performance of Nb_2_CT_*x*_ MXene. Reproduced with permission from [119] Copyright 2021, Wiley–VCH. **d** CV curves of Ti_2_CT_*x*_ and Ti_3_C_2_T_*x*_ MXene. Reproduced with permission from [112] Copyright 2021, The Authors, published by Springer Nature. **e** SEM image of Ti_3_C_2_T_*x*_ MXene prepared from 60 batch of Ti_3_AlC_2_ MAX. **f** Specific capacities of Ti_3_C_2_T_*x*_, Ti_2_CT_*x*_ and Ti_3_CNT_*x*_ MXene at various C-rates. Reproduced with permission from [175] Copyright 2022, Zhengzhou University. **g** Elemental composition comparison of Ti_3_C_2_F_*x*_, Ti_3_C_2_Cl_*x*_ and Ti_3_C_2_Br_*x*_ MXenes. **h** Long-term cyclic performance of Ti_3_C_2_F_*x*_, Ti_3_C_2_Cl_*x*_ and Ti_3_C_2_Br_*x*_ MXenes at 1 A g^−1^. Reproduced with permission from [178] Copyright 2022, Elsevier. **i** TEM image of Ti_3_C_2_T_*x*_/Sn anode after 2200 cycles. Reproduced with permission from [145] Copyright 2022, Elsevier

In the past three decades, significant progress has been made in the development of LIBs technology. Derived from the high power and energy density, long cycle life, high energy conversion efficiency and low discharge rate, LIBs system has now been widely used in consumer devices, electric vehicles and large-scale energy storage [166, 167]. Layered materials have been intensively studied as electrode materials for LIBs due to their unique structural stability. As early as 2012, Tang et al. confirmed that Ti_3_C_2_ MXene possesses low Li^+^ diffusion barrier, superior electronic conductivity, low discharge voltage and high lithium storage capacity via theoretical computation, making it a promising LIBs anode [59]. Since then, a large number of works have experimentally proved that MXene-based anodes can deliver relatively high discharge capacity, stable long-term cyclic performance and outstanding rate performance [72, 168–171]. The theoretical capacities of MXene electrodes depends on formula weight, interlayer spacing as well as the type of transition metals and surface groups. MXenes with low formula weights such as V_2_C and Ti_2_C can afford large gravimetric capacities [88, 172, 173]. In addition, large interlayer spacing can allow more Li^+^ ions to intercalate into the MXenes, accordingly leading to higher specific capacities [30, 174]. Surface functional groups such as –F and –OH has been proved to impede Li^+^ transportation via theoretical calculations, which is unfavorable for realization of theoretical specific capacity [59, 172]. However, O-terminated Ti_3_C_2_ MXene can absorb two Li layers, confirming the lithium affinity of –O functional groups and thus contributing to the enhanced lithium storage capacity (Fig. 10a) [120].

It is worth mentioning MXenes obtained by Lewis acidic etching method may exhibit better electrochemical performance due to the absence of –F and –OH surface groups and the introduction of –O termination. In 2020, Li et al. prepared multi-layered Ti_3_C_2_T_*x*_ MXene (T = –O and –Cl) via CuCl_2_ etching of Ti_3_SiC_2_ precursor [67]. When used as anode for LIBs, the obtained Ti_3_C_2_T_*x*_ MXene exhibit a distinct pseudocapacitive-shaped cyclic voltammetry (CV) curves without redox peaks in organic electrolyte. In comparison, MXene anodes synthesized by HF etching generally show obvious redox peaks in CV profiles [168, 171]. In addition, Ti_3_C_2_T_*x*_ MXene delivers a discharge capacity of 205 mAh g^−1^ at 0.6C rate, corresponding to the fact that per Ti atom can transfer around 0.4 electrons. Further, superior long-term cyclic stability up to 2400 cycles can be obtained for multi-layered Ti_3_C_2_T_*x*_ MXene electrode. The superior lithium storage performance can be attributed to the strong adsorption ability of -O surface group on Li^+^ cations [120]. Moreover, the Li^+^ insertion/deinsertion storage mechanism can be confirmed by the enlarged/decreased interlayer spacing through in-situ XRD measurement. In 2021, the Nb_2_CT_*x*_ MXene (T = –O and –Cl) with obvious accordion-like morphology was prepared by CuCl_2_ molten salt etching of Nb_2_AlC precursor [119]. The theoretical calculations indicate that –O surface groups can obtain more electrons from Nb atoms than –Cl termination for Nb_2_CT_*x*_ MXene, thus resulting in the substitution of –O for –Cl functional groups during ammonium persulfate solution washing process, which is conducive to its lithium storage performance. Nb_2_CT_*x*_ MXene anode delivers a high discharge capacity of 330 mAh g^−1^ at 50 mA g^−1^ (Fig. 10b), which is larger than the theoretical specific capacities of Nb_2_C (305 mAh g^−1^) and Nb_2_CO_2_ (292 mAh g^−1^). Additionally, capacity rising phenomenon can be clearly observed for Nb_2_CT_*x*_ MXene at 1000 mA g^−1^ (Fig. 10c), which can be attributed to the electrochemical activation and common pillaring effect.

In 2022, Lin et al. prepared Ti_2_CT_*x*_ and Ti_3_C_2_T_*x*_ MXene by one-pot method in an air atmosphere with Ti, C, and Al powders as precursor, simultaneously achieving the synthesis of MAX and in-situ CuCl_2_ etching in molten salts system [112]. When served as anodes for LIBs, Ti_2_CT_*x*_ and Ti_3_C_2_T_*x*_ electrode both exhibit rectangular and symmetric CV curves in a voltage range of 0.1–2 V (Fig. 10d), which is similar to those of previously reported MXene electrodes prepared by Lewis acidic etching [67]. Furthermore, Ti_2_CT_*x*_ anode shows discharge capacities of 256 and 164 mAh g^−1^ at 0.5 and 10 mV s^−1^, respectively. In comparison, specific capacities of 164 and 113 mAh g^−1^ can be obtained for Ti_3_C_2_T_*x*_ electrode at the same scan rate. The superior rate performance of these two MXene electrodes is mainly due to the pseudocapacitive-controlled charge storage mechanism. Later, Chen et al. reported the synthesis of Ti_3_C_2_T_*x*_, Ti_2_CT_*x*_, Ti_3_CNT_*x*_ and Ti_4_N_3_T_*x*_ MXene via molten salts-shielded synthesis strategy in air atmosphere with MAX phases as precursor and CuCl_2_ molten salt as etchant [175]. The low-melting eutectic salt reaction medium can effectively restrain the oxidation of MXenes at high temperature. More importantly, the proposed method can be used to prepare Ti_3_C_2_T_*x*_ MXene on a relatively large scale. The distinct accordion-like structure can still be obtained for Ti_3_C_2_T_*x*_ MXene prepared from 60 g batch of Ti_3_AlC_2_ MAX (Fig. 10e), demonstrating superior scalability. Among the Ti_3_C_2_T_*x*_, Ti_2_CT_*x*_ and Ti_3_CNT_*x*_ MXene electrodes, Ti_2_CT_*x*_ anode delivers the largest discharge capacity, and the three electrodes all exhibit excellent rate performance (Fig. 10f).

Recently, Cao et al. demonstrated the successful preparation of multi-layered Ti_2_NT_*x*_ MXene (T = –O and vCl) via CuCl_2_ etching at 450 °C for 1 h [176]. The existence of Ti-Cl and Ti–O bonds can be confirmed by XPS and XAS measurement. Theoretical calculations indicate that the Gibbs energy change of –O substituting –Cl termination is −2.3 eV, while the value of –O replacing –F surface group is 0.07 eV, demonstrating that –Cl can be readily substituted by –O. The obtained Ti_2_NT_*x*_ MXene can deliver high discharge capacities of 303.4 and 158.4 mAh g^−1^ at 0.1 and 5 A g^−1^, respectively. More importantly, a large capacity of 350 mAh g^−1^ after 1,200 cycles at 1 A g^−1^ can be obtained for Ti_2_NT_*x*_ electrode, revealing outstanding cycling stability. A hybrid capacitor by pairing Ti_2_NT_*x*_ anode and super activated carbon cathode can demonstrate an energy density of 45.36 Wh kg^−1^ at 123.32 W kg^−1^. It is noteworthy that the MXene electrodes mentioned above are all at multi-layered state, and few-layered MXene electrodes may show better electrochemical performance. Liu et al. reported the synthesis of exfoliated Ti_3_C_2_T_*x*_ MXene (termed as e-MS-Ti_3_C_2_T_*x*_) via CuCl_2_ molten salts etching and subsequent TBAOH intercalation [153]. The e-MS-Ti_3_C_2_T_*x*_ electrode delivers discharge capacities of 225 and 95 mAh g^−1^ at 0.2 and 16 A g^−1^, respectively, which is higher than that of unexfoliated Ti_3_C_2_T_*x*_ anode. The boosted rate performance of e-MS-Ti_3_C_2_T_*x*_ electrode is mainly assigned to the enhanced contact area between electrode and electrolyte after exfoliation. Additionally, the increased content of -O termination after TBAOH treatment will also benefit the lithium storage performance [177].

The type of halogen terminations may have an influence on the electrochemical performance of MXene. Liu et al. synthesized Ti_3_C_2_T_*x*_ MXene with –F, –Cl or –Br functional groups via HF, NiCl_2_ or NiBr_2_ etching, respectively [178]. The typical accordion morphology can be afforded for three halogenated MXenes. The element composition analysis shows that Ti_3_C_2_Br_*x*_ MXene possesses the highest content of –O surface group among the Ti_3_C_2_F_*x*_, Ti_3_C_2_Cl_*x*_ and Ti_3_C_2_Br_*x*_ MXene (Fig. 10g). This phenomenon is mainly assigned to the fact that the formation energy of Ti–Br bond is the lowest compared with that of Ti–Cl and Ti–F bonds according to theoretical calculations. Consequently, –O can easily replace the -Br surface group when the prepared Ti_3_C_2_Br_*x*_ MXene is exposed to air, and the highest content of -O functional group contributes to the largest discharge capacity of 189 mAh g^−1^ for Ti_3_C_2_Br_*x*_ anode. Finally, the Ti_3_C_2_Br_*x*_ electrode exhibits the best rate performance and cyclic performance relative to Ti_3_C_2_F_*x*_ and Ti_3_C_2_Cl_*x*_ anode (Fig. 10h). The electrode materials mentioned above are all individual MXenes, while the MXene/active metal composites can also be utilized as anodes for LIBs. Wu et al. reported the preparation of Sn-nanoconfined Ti_3_C_2_T_*x*_ MXene hybrids via SnCl_2_ molten salt etching of Ti_3_AlC_2_ [145]. The Sn nanoparticles are in-situ grown and confined between the Ti_3_C_2_T_*x*_ MXene during the etching process. It is noteworthy that the confined effect of MXene can effectively prevent the aggregation of Sn nanoparticles at high temperature and accommodate its large volume variation upon cycling. As a result, Ti_3_C_2_T_*x*_/Sn anode remains a reversible capacity of 226.2 mAh g^−1^ after 1000 cycles at 0.2 A g^−1^, which is higher than that of pure Ti_3_C_2_T_*x*_ and Sn electrode. The volume expansion of Sn nanoparticles can gradually enlarge the interlayer spacing of MXene from 1.17 to 1.34 nm after 2200 cycles (Fig. 10i), which greatly exposes more active sites upon cycling and contributes to the obvious capacity rising phenomenon.

#### Sodium-Ion Batteries

**Fig. 11:**
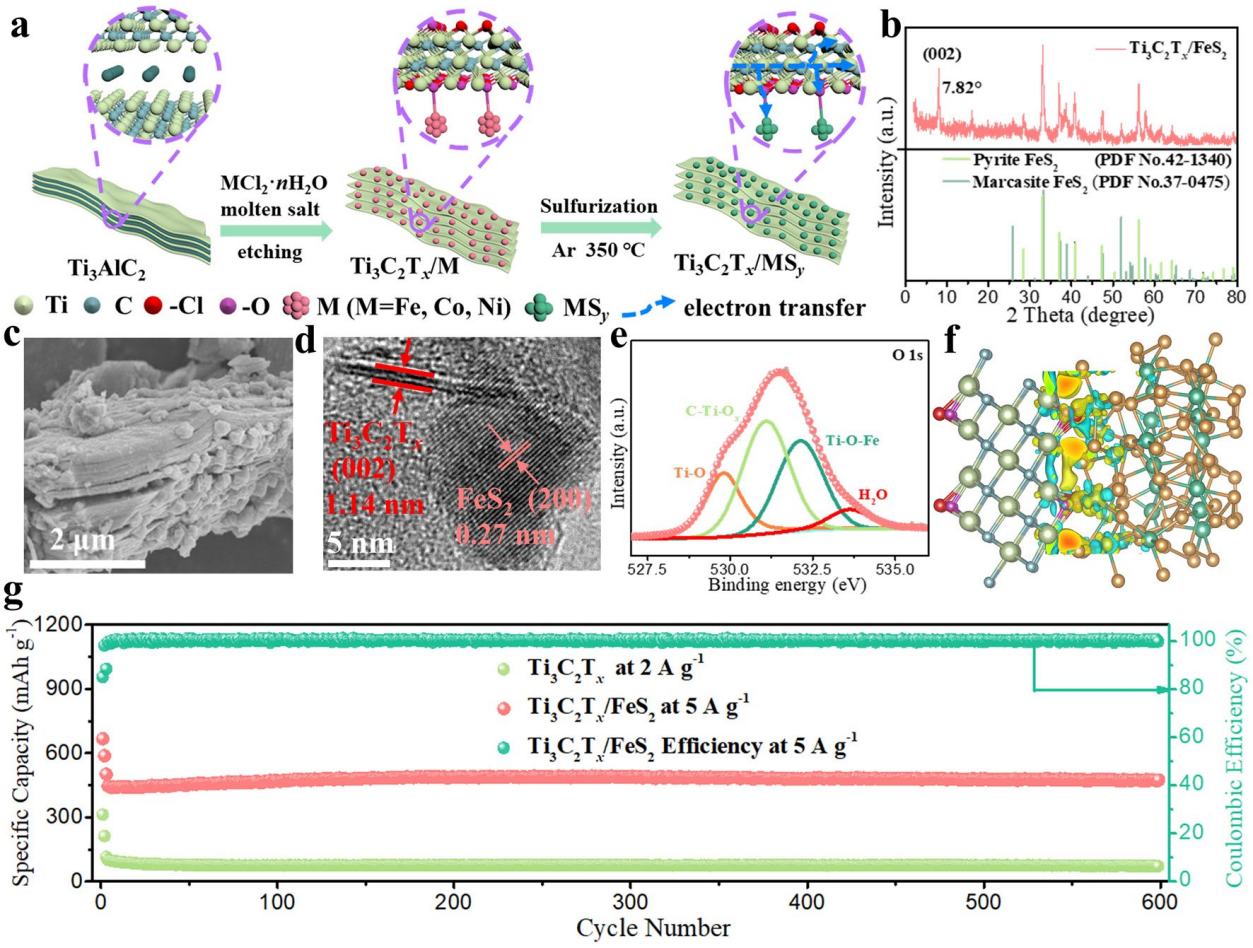
**a** Schematic of the fabrication process of Ti_3_C_2_T_*x*_/MS_*y*_ hybrids. **b** XRD pattern of Ti_3_C_2_T_*x*_/FeS_2_ composites. **c** SEM image of Ti_3_C_2_T_*x*_/FeS_2_ hybrids. **d** HRTEM image of Ti_3_C_2_T_*x*_/FeS_2_. **e** O 1* s* XPS spectra of Ti_3_C_2_T_*x*_/FeS_2_. **f** Charge density difference of Ti_3_C_2_T_*x*_/FeS_2_ composites. **g** Cyclic performance of Ti_3_C_2_T_*x*_ MXene and Ti_3_C_2_T_*x*_/FeS_2_ hybrids at various current densities. Reproduced with permission from [147] Copyright 2022, Wiley–VCH

SIBs possess a similar working mechanism to LIBs. It is well known that the working voltage of SIBs is lower than that of LIBs, thus resulting in a relatively low energy density of SIBs [179, 180]. However, due to the abundance of sodium resources and attractive cost-effectiveness [181], SIBs are promising to be utilized in some fields that do not require high energy density, such as large-scale energy storage [182, 183]. As a type of negative electrode for SIBs, transition metal sulfides exhibit high theoretical specific capacities and safe discharge voltage [184]. Nevertheless, large volume expansion and inferior charge transfer kinetics of transition metal sulfides greatly deteriorate their sodium storage performance [185]. Our group has been working on improving the electrochemical performance of transition metal compounds in the past few years [73, 117]. Recently, we proposed a general strategy for constructing Ti_3_C_2_T_*x*_ MXene/transition metal sulfides (Ti_3_C_2_T_*x*_/MS_*y*_, T = –O and –Cl, M = Fe, Co and Ni) heterostructures as SIBs anodes through directly sulfurizing Lewis acidic etching products (MXenes/metal composites) (Fig. 11a–b) [147]. Different from the traditional construction process of preparing MXene first, then adsorbing transition metal ions and finally vulcanizing, this strategy simplifies the preparation procedure of MXene/transition metal sulfides hybrids via rationally utilizing Lewis acidic etching products. Additionally, this fabrication process avoids the use of HF as etchant and water as reaction medium, which effectively improves the experimental safety and alleviates the oxidation of MXene, respectively. For the fabricated Ti_3_C_2_T_*x*_/FeS_2_ heterostructures, FeS_2_ nanoparticles are in-situ grown and tightly fixed on the Ti_3_C_2_T_*x*_ MXene substrate (Fig. 11c–d), demonstrating a stable framework for continuous Na^+^ insertion/extraction. Furthermore, O 1* s* XPS spectrum and theoretical calculations confirm that Ti_3_C_2_T_*x*_ MXene and FeS_2_ nanoparticles demonstrate interfacial electronic coupling effect via Ti–O–Fe bonds (Fig. 11e–f), facilitating the rapid interfacial electron transfer from MXene to FeS_2_. Additionally, Ti_3_C_2_T_*x*_/FeS_2_ hybrids also exhibit rapid Na^+^ diffusion kinetics and mechanical strain release channels. Consequently, derived from the above advantages, the Ti_3_C_2_T_*x*_/FeS_2_ anode delivers a reversible capacity of 474.9 mAh g^−1^ after 600 cycles at 5 A g^−1^ (Fig. 11g), and reveals a high capacity of 456.6 mAh g^−1^ at 10 A g^−1^, suggesting outstanding long-term cyclic performance and rate capability. In addition, the full-cell device assembled with Ti_3_C_2_T_*x*_/FeS_2_ anode and Na_3_V_2_(PO_4_)_3_ cathode can demonstrate a discharge capacity of 431.6 mAh g^−1^ after 1000 cycles at 3 A g^−1^ and an energy density of around 130 Wh kg^−1^ at a power density of 415 W kg^−1^, indicating superior application prospect of Ti_3_C_2_T_*x*_/FeS_2_ heterostructures.

Copper sulfide (CuS), as another common transition metal sulfide, also possesses high theoretical capacity and low price, and has been widely studied as anode for SIBs. However, electrode pulverization and inferior electrochemical reaction kinetics have an adverse effect on its performance [186]. In order to improve the sodium storage performance of CuS, our group readily fabricated Ti_3_C_2_T_*x*_/CuS hybrids (T = –O and –Cl) via CuCl_2_·2H_2_O etching and following in-situ sulfurization treatment [148]. The construction process also achieves the effective application of Lewis acidic molten salts etching products. Plenty of CuS nanoparticles are distributed among the Ti_3_C_2_T_*x*_ MXene substrate, and they are connected by Ti–O–Cu covalent bonds. Benefiting from the extremely boosted electron transfer and Na^+^ migration, enhanced sodium adsorption and structural integrity, the obtained Ti_3_C_2_T_*x*_/CuS anode delivers stable long-term cyclic performance with a discharge capacity of 347 mAh g^−1^ after 800 cycles at 3 A g^−1^ and wonderful rate performance with a high capacity of 346.3 mAh g^−1^ at 8 A g^−1^. More importantly, the constructed SIBs full cell with Na_3_V_2_(PO_4_)_3_ cathode and Ti_3_C_2_T_*x*_/CuS anode reveals a capacity of 189.6 mAh g^−1^ after 800 cycles at 2 A g^−1^. In the above-mentioned two works, MXene/transition metal sulfide heterostructures are efficiently constructed by rationally utilizing Lewis acidic etching products, namely MXenes/metal composites, which fully reflects the great advantages of Lewis acidic etching relative to other etching methods.

#### Zinc-Ion Batteries

**Fig. 12:**
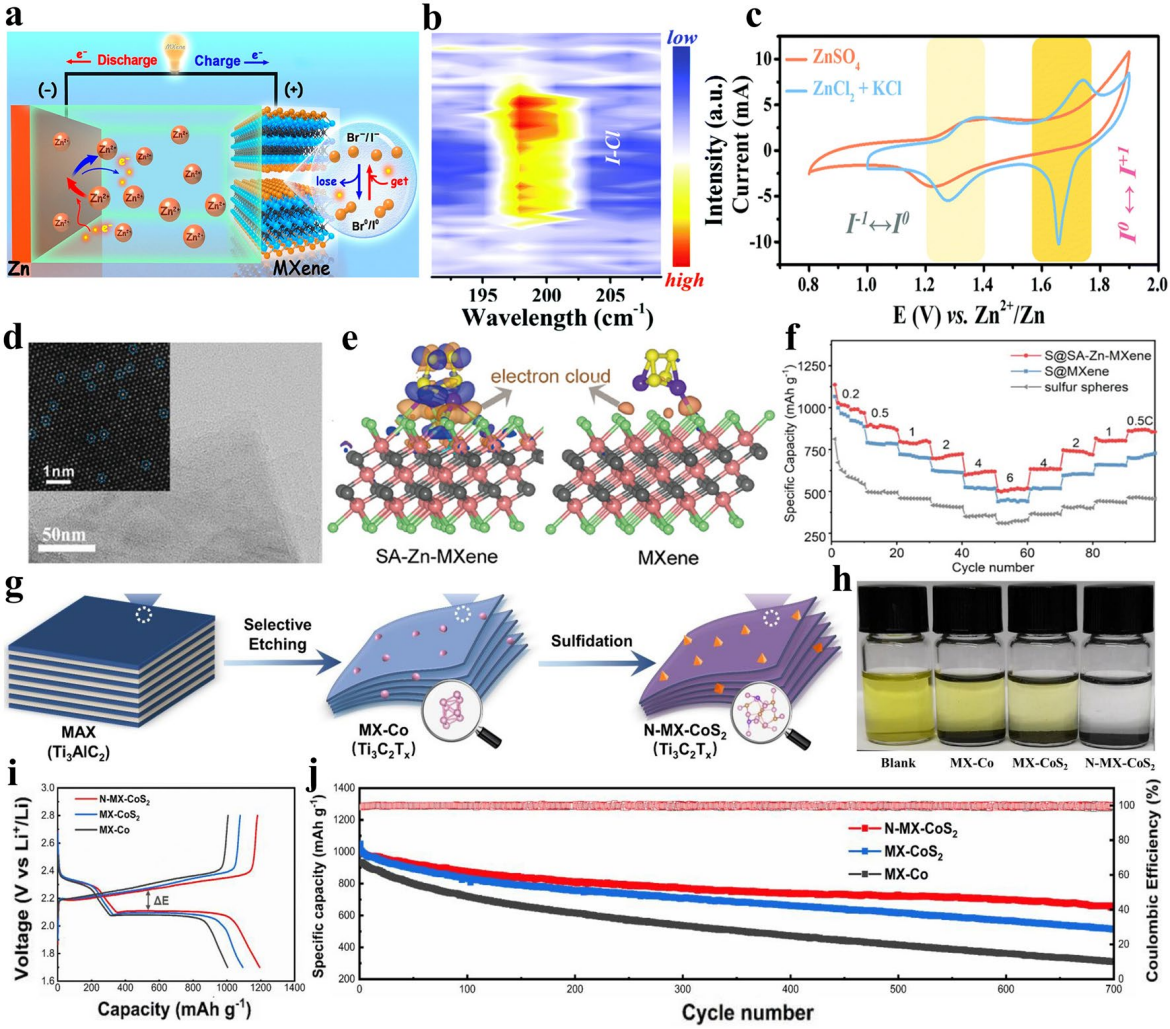
**a** Schematic diagram of the working mechanism of Zn/halogenated Ti_3_C_2_ MXene cells. Reproduced with permission from [123] Copyright 2021, American Chemical Society. **b** In-situ Raman spectra of the Ti_3_C_2_I_2_ cathode around 200 cm^−1^. **c** CV curves of Ti_3_C_2_I_2_ cathode in ZnCl_2_ + KCl electrolyte or conventional ZnSO_4_ electrolyte. Reproduced with permission from [194] Copyright 2021, Royal Society of Chemistry. **d** HAADF-STEM image of SA-Zn-MXene. **e** Charge density difference for Li_2_S_4_ polysulfides adsorbed on SA-Zn-MXene and MXene. **f** Rate capabilities of S, S@MXene and S@SA-Zn-MXene. Reproduced with permission from [157] Copyright 2020, Wiley–VCH. **g** Schematic of the preparation process of N-MX-CoS_2_ hybrids. **h** Adsorption test of MX-Co, MX-CoS_2_ and N-MX-CoS_2_. **i** Galvanostatic charge–discharge curves of Li–S batteries with various materials-modified separator at 0.2C. **j** Long-term cyclic stability of Li–S batteries with various materials-treated separators at 1 C. Reproduced with permission from [146] Copyright 2021, Elsevier

Due to the flammability of organic electrolyte, LIBs and SIBs systems possess inevitable potential safety hazards, resulting in the combustion of electric vehicles and large-scale energy storage devices from time to time in the past few years [187]. Therefore, ZIBs system has recently attracted considerable attention owing to the use of aqueous electrolyte, which possesses high safety and low price [188, 189]. Additionally, ZIBs also demonstrate other important merits, such as high eco-efficiency and ionic conductivity of approximately 1 S cm^−1^ [190]. The halogen electrodes including Br_2_ and I_2_ exhibit conversion reaction mechanism and high theoretical specific capacities, and have been widely investigated as cathode materials for ZIBs [191–193]. Fortunately, the MXenes obtained by Lewis acidic etching can be endowed with –Br or –I functional groups, so it is necessary to investigate their zinc storage performance (Fig. 12a). In 2021, Li et al. prepared Ti_3_C_2_ MXene with –Cl, –Br and –I functional groups through CuCl_2_, CuBr_2_ and CuI etching, respectively [123]. When applied as cathodes for ZIBs, the Ti_3_C_2_Br_2_ and Ti_3_C_2_I_2_ MXenes exhibit obvious redox peaks in CV profiles and flat discharge–charge plateaus in galvanostatic charge–discharge curves, while Ti_3_C_2_Cl_2_ MXene shows no redox peaks and oblique charge–discharge curves. The conversion reaction process of Ti_3_C_2_Br_2_ and Ti_3_C_2_I_2_ MXene is illustrated as follows:

27 $${\text{2Br}}^{ - } - {\text{2e}}^{ - } \to {\text{2Br}}^{0}$$

28 $${\text{2I}}^{ - } - {\text{2e}}^{ - } \to {\text{2I}}^{0}$$

In contrast, the Cl^−^ anions in Ti_3_C_2_Cl_2_ MXene will loss electrons and turn into Cl_2_ gas, which may escape from the MXene interlayer, making this conversion reaction irreversible. Consequently, Ti_3_C_2_Br_2_ and Ti_3_C_2_I_2_ MXene demonstrate energy densities of 129 and 135.6 Wh kg^−1^, respectively, which is significantly higher than that of Ti_3_C_2_Cl_2_ MXene. This work realizes the application of halogen terminations for the first time, and also suggests that the electrochemical performance of MXenes can be greatly improved by regulating the surface groups.

It is worth mentioning that the reaction mechanism of above-mentioned Ti_3_C_2_I_2_ MXene is still stopping at individual I^−^/I^0^ redox pair, thus resulting in a low discharge voltage of around 1.3 V when used as ZIBs cathode. If the I^0^/I^+^ redox pair can be activated, the discharge voltage can be further increased. However, I^+^ cations are unstable in common electrolytes such as ZnSO_4_ solution. In order to solve this issue, Li et al. first synthesized Ti_3_C_2_I_2_ MXene via CuI etching and then chose electrolyte system that contains electronegative F^−^ or Cl^−^ anions, which can considerably stabilize the I^+^ cations during the cycling process [194]. Specifically, once I^+^ cations are generated, F^−^ or Cl^−^ anions can combine with them, making I^+^ cations exist stably, which can be effectively confirmed by the peak at 200 cm^−1^ in the in-situ Raman spectra of the Ti_3_C_2_I_2_ MXene (Fig. 12b). Therefore, the I^0^/I^+^ redox pair are successfully activated in ZnCl_2_ + KCl electrolyte and a higher reduction peak at 1.66 V can be observed (Fig. 12c). As expected, Ti_3_C_2_I_2_ MXene in ZnCl_2_ + KCl electrolyte affords a large discharge capacity of 207 and 126 mAh g^−1^ at a current density of 0.5 and 5 A g^−1^, respectively, showing superior rate performance. Additionally, outstanding cyclic stability can be delivered for Ti_3_C_2_I_2_ MXene with approximately 80% capacity retention after 2800 cycles. The excellent zinc storage performance is attributed to the rapid electron/Zn^2+^ transfer kinetics and strong confinement effect of MXene that prevents interlayer iodine active species from escaping to the outside. In short, the additional high discharge plateau at 1.65 V endows Ti_3_C_2_I_2_ MXene with a large energy density of 280 Wh kg^−1^. The above two works indicate that MXenes prepared by Lewis acidic molten salts etching has broad development space in aqueous ZIBs.

#### Lithium-Sulfur Batteries

Li–S batteries have received extensive attention since its birth in 1962. Compared with LIBs, SIBs and ZIBs systems, Li–S batteries demonstrate a very high theoretical energy density of 2600 Wh kg^−1^ and have been regarded as one of the most promising energy storage devices in the future [195, 196]. However, ultralow electronic conductivity of sulfur (10^–30^ S cm^−1^) and Li_2_S, sluggish reaction kinetics, severe shuttle effect of LiPSs and low utilization rate of sulfur result in inferior rate performance and cyclic durability [197]. Luckily, MXenes are expected to improve the electrochemical performance of Li–S batteries owing to their metallic conductivity, chemical adsorption ability, catalytic effect and structural diversity [198, 199]. In 2020, single atom zinc implanted Ti_3_C_2_T_*x*_ MXene (SA-Zn-MXene) as sulfur host was successfully fabricated via ZnCl_2_ etching of Ti_3_AlC_2_ MAX [157]. They found that Zn^2+^ cations in ZnCl_2_ molten salt can not only react with the interlayer Al atoms, but also replace a few Ti species in MXene lattice during the etching process, which can be further confirmed by the bright dots corresponding to Zn atoms in HAADF-STEM image (Fig. 12d). On one hand, the SA-Zn-MXene exhibits strong interaction with Li_2_S_4_ polysulfides (Fig. 12e), and can catalyze the polysulfides transformation by decreasing the reaction barriers. On the other hand, the nucleation of Li_2_S_2_/Li_2_S can be significantly boosted by Zn atoms in MXene lattice. Additionally, the large volume variation of sulfur upon cycling can be accommodated by flexible Ti_3_C_2_T_*x*_ MXene. Consequently, the S@SA-Zn-MXene electrode exhibits the lowest overpotential of 23 mV and superior rate performance with a high capacity of 517 mAh g^−1^ at 6C (Fig. 12f). More importantly, the enhanced cyclic stability can be obtained for S@SA-Zn-MXene, which delivers a discharge capacity of 706 mAh g^−1^ after 400 cycles at 1C.

Polar metal compounds including metal oxides, metal sulfides, metal selenides, metal nitrides, metal borides and metal phosphides have been considered as effective electrocatalysts to accelerate reaction kinetics of LiPSs [200–202]. In 2022, Yang et al. obtained N-doped Ti_3_C_2_T_*x*_ MXene-CoS_2_ heterostructure (N-MX-CoS_2_) via CoCl_2_ etching and subsequent in-situ sulfidation treatment with thiourea as sulfur source (Fig. 12g), and then utilized N-MX-CoS_2_ as a bifunctional catalyst to modify the commercial separator [146]. The N element can be doped into the hybrids during the process of thiourea pyrolysis, which can be proved by the existence of N–C and Ti–N peaks in XPS spectra. This strategy enables CoS_2_ nanoparticles to be tightly anchored among the MXene substrate. In the N-MX-CoS_2_ hybrids, the N atoms and polar CoS_2_ nanoparticles can form strong chemical interaction with LiPSs and provide rich catalytic sites to facilitate LiPSs conversion reaction. The Ti_3_C_2_T_*x*_ MXene with metallic-level conductivity can further promote sulfur redox reaction kinetics. Since N-MX-CoS_2_ heterostructure possesses dual functions of adsorption and catalysis, the shuttle effect of LiPSs can be effectively inhibited and polarization can be greatly decreased (Fig. 12h–i). Finally, the batteries with modified separator deliver superior rate performance (775 mAh g^−1^ at 4C) and excellent long-term cycling stability with a low decay rate of 0.052% per cycle (651 mAh g^−1^ after 700 cycles at 1C and 721.6 mAh g^−1^ after 400 cycles at 2C) (Fig. 12j). To our relief, a series of MXenes/metal compound composites with strong binding energy can also be synthesized via Lewis acidic etching route, which is worthy of in-depth exploration and may provide new inspiration to the further development of Li–S batteries.

#### Metal Anodes-Based Batteries

Metal anodes including Li, Na, K, Al, Mg and Zn have been intensively investigated in high-performance secondary metal batteries in the past few decades owing to their low plating/stripping potentials and high theoretical specific capacities, thus leading to high energy densities [203–206]. Nevertheless, there are some key factors that deteriorate the electrochemical performance of metal anodes. First, the uneven deposition of metals during the discharge process will contribute to the uncontrolled growth of dendrites, which can pierce the separator and result in the short-circuit of cells. Then, the nearly infinite volume expansion leads to the rupture of solid electrolyte interphase (SEI) layer. In addition, the side reactions and the formation of “dead metal” will continuously consume the electrolyte and metal sources. Consequently, metal anodes generally demonstrate low coulombic efficiency, short cycle life and serious safety hazards [207, 208]. Some common strategies such as optimizing electrolyte composition, building artificial SEI film and fabricating three-dimensional robust frameworks have been utilized to enhance the performance of metal anodes [209]. Fortunately, MXenes with high electronic conductivity, low ion migration barrier, unique layered structure and sufficient functional groups can greatly facilitate the plating/stripping kinetics, effectively preventing the dendrite growth [15, 210].

**Fig. 13:**
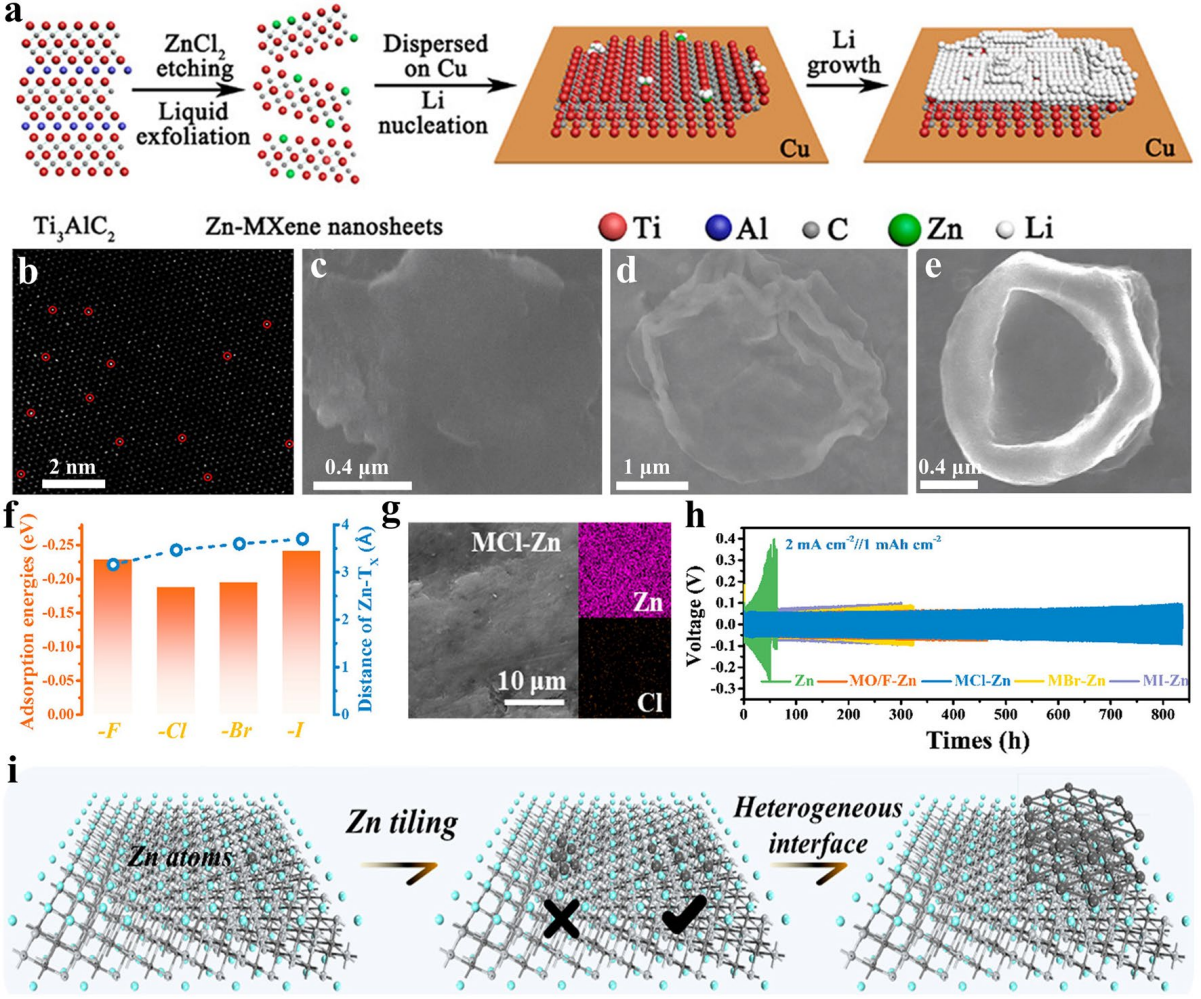
**a** Schematic showing the preparation of Zn-MXene for Li nucleation and growth. **b** HAADF-STEM image of Zn-MXene nanosheets. **c-e** SEM images of Zn-MXene nanosheets after Li plating with capacities of 5, 20 and 60 μAh cm^−2^, respectively. Reproduced with permission from [156] Copyright 2020, American Chemical Society. **f** Calculated atomic distance between Zn atom and neighboring halogen termination as well as Zn adsorption energies of different MXene surfaces. **g** SEM image and EDS mapping of the Ti_3_C_2_Cl_2_-Zn electrode after cycling. **h** Long-term cyclic performance of various symmetric cells. **i** Schematic diagram of the Zn deposition process on the MXene substrate. Reproduced with permission from [212] Copyright 2021, American Chemical Society

For example, single zinc atoms immobilized Ti_3_C_2_Cl_*x*_ MXene (Zn-MXene) as a host for lithium metal was reported through ZnCl_2_ molten salts etching (Fig. 13a–b), and the content of Zn monoatoms is around 0.87 at% [156]. It is worth mentioning that sufficient reaction time is conducive to the substitution of Zn for Ti. Due to the lithiophilic nature of Zn, the lithium metal can preferentially nucleate around the single atom Zn sites on the surface of Ti_3_C_2_Cl_*x*_ MXene. When the plating level increases to 5 μAh cm^−2^, a flat and smooth morphology can be obtained (Fig. 13c). Furthermore, Li metal grows vertically along the nucleation site to form a bowl-shaped lithium at higher deposition amounts (Fig. 13d–e), finally achieving dendrite-free Li metal anode. Therefore, Zn-MXene films can exhibit a low nucleation overpotential of 11.3 mV for half battery and a long cycle life of 1200 h for symmetric cells at 1 mA cm^−2^ with a fixed capacity of 1 mAh cm^−2^. In addition, the Zn-MXene//LiFePO_4_ full cell delivers a high capacity of 150 mAh g^−1^ at 0.5C and superior cyclic stability with a capacity of about 100 mAh g^−1^ after 500 cycles at 10C.

In recent years, MXenes have attracted extensive attention in inhibiting the growth of Zn dendrites due to their superior lattice matching [211]. More importantly, the surface groups of MXenes are considered to affect the adsorption energy and migration kinetics of Zn^2+^ ions. Based on this point, Li et al. reported the synthesis of various halogenated MXenes including Ti_3_C_2_Cl_2_, Ti_3_C_2_Br_2_ and Ti_3_C_2_I_2_ MXene as interface layers via corresponding Lewis acidic molten salts etching to regulate the Zn deposition [212]. They found that above-mentioned halogenated MXenes possess metallic-level conductivities and good affinity to Zn metal via theoretical simulation (Fig. 13f), which is conducive to the deposition of Zn^2+^ ions. Consequently, Zn^2+^ ions tend to grow horizontally rather than vertically under the regulation of outermost halogen surface groups, which effectively inhibits the disordered growth of Zn metal at the beginning. Subsequently, the constructed coherent heterogeneous interface induces the uniform deposition of Zn metal (Fig. 13i). As expected, the obtained halogenated MXene-Zn anode exhibits better electrochemical performance than bare Zn metal anode. Among the MXenes with –Cl, –Br or –I terminations, the –Cl functionalized Ti_3_C_2_ MXene-Zn anode possesses the strongest ability to inhibit the growth of Zn dendrites derived from its medium adsorption and diffusion for Zn^2+^. Consequently, Ti_3_C_2_Cl_2_-Zn electrode exhibits a flat and smooth surface after cycling, and a cycle life of 840 h at 2 mA cm^−2^ with a fixed capacity of 1 mAh cm^−2^ for symmetric cells (Fig. 13g–h). Finally, the Ti_3_C_2_Cl_2_-Zn//Ti_3_C_2_I_2_ full cell demonstrates superior long-term cyclic stability up to 9,000 cycles, which is better than that of Zn//Ti_3_C_2_I_2_ battery. The above two works indicate that MXenes obtained by Lewis acidic etching have a good induction effect on the uniform growth of metal anodes.

#### Dual-Ion Batteries

**Fig. 14:**
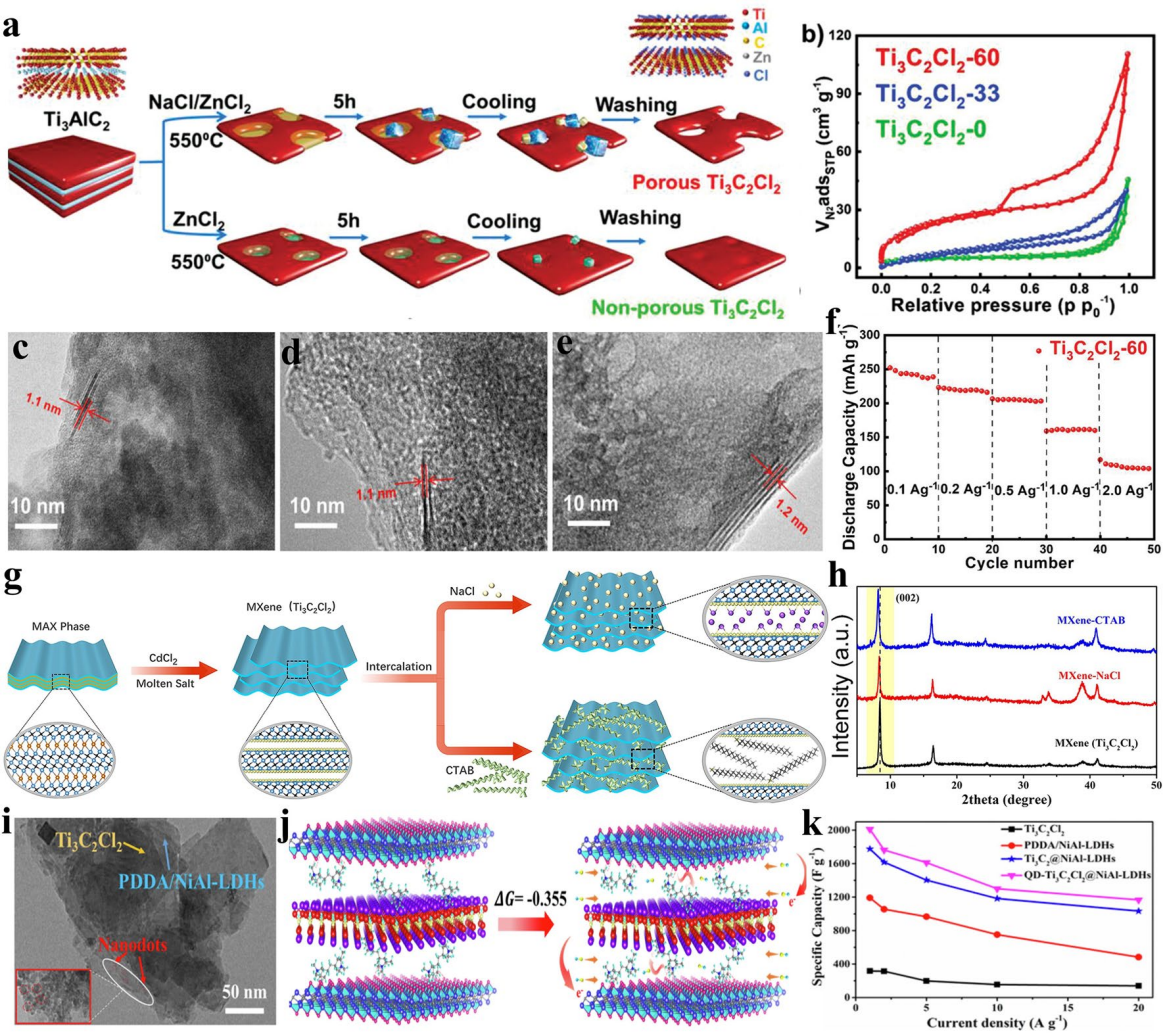
**a** Schematic showing the preparation of in-plane porous Ti_3_C_2_Cl_2_ MXene via eutectic etching. **b** N_2_ adsorption–desorption isotherms of various MXenes. TEM images of Ti_3_C_2_Cl_2_ MXene obtained by eutectic etching with **c** 0%, **d** 33% and **e** 60% NaCl mole percent in the NaCl/ZnCl_2_ mixture. **f** Rate performance of Ti_3_C_2_Cl_2_-60/graphite DIBs. Reproduced with permission from [113] Copyright 2021, Wiley–VCH. **g** Schematic demonstrating the preparation of MXene-NaCl and MXene-CTAB. **h** XRD patterns of MXene, MXene-NaCl and MXene-CTAB. Reproduced with permission from [219] Copyright 2021, Elsevier. **i** HRTEM image of QD-Ti_3_C_2_Cl_2_@NiAl-LDHs composites. **j** The free energy diagram of pseudocapacitive process on the QD-Ti_3_C_2_Cl_2_@NiAl-LDHs. **k** Specific capacities of the various electrodes at different current densities. Reproduced with permission from [222] Copyright 2021, Elsevier

As a novel battery concept, the dual-ion batteries (DIBs) demonstrate a brand-new working mechanism, which is realized by intercalating the anions and cations of electrolyte into respective electrodes. In general, DIBs possess some advantages such as high theoretical energy density, low cost and long cycle life [213]. Nevertheless, low anode specific capacity of DIBs still remains a big problem. MXenes, as a type of 2D layered material, have abundant nano-level channels to allow the insertion and de-insertion of ions and can be regarded as electrodes for DIBs. In 2022, Ti_3_C_2_Cl_2_ MXene with adjustable in-plane porosity was obtained by eutectic molten salts (NaCl/ZnCl_2_) etching (Fig. 14a) [113]. The formation mechanism of in-plane pore has been discussed previously in detail. Specifically, the surface area and mesoporous volume of Ti_3_C_2_Cl_2_ MXene increase gradually with the rise of NaCl content in ZnCl_2_/NaCl mixture (Fig. 14b–e), which can create more active sites and benefit the Li^+^ migration. For example, when the mole percent of NaCl in ZnCl_2_/NaCl mixture is 60%, the obtained Ti_3_C_2_Cl_2_-60 MXene delivers a specific surface area of 85 m^2^ g^−1^ and pore size concentrates on 3–4 nm. Additionally, Ti_3_C_2_Cl_2_-60 demonstrates superior TiC_6_ octahedral core symmetry and low Li^+^ migration kinetics. Consequently, the Ti_3_C_2_Cl_2_-60 MXene as LIBs anode delivers a discharge capacity of 382 mAh g^−1^ at 0.1 A g^−1^. Further, when paired with graphite cathode, Ti_3_C_2_Cl_2_-60/graphite DIBs afford a high discharge capacity of 242 mAh g^−1^ at 0.1 A g^−1^ (Fig. 14f), and the superior cyclic stability with 83% capacity retention can also be obtained (141 mAh g^−1^ after 1000 cycles at 1 A g^−1^), demonstrating excellent electrochemical performance. The in-plane porous MXene produced by Lewis acidic molten salts etching can also be utilized in other fields such as catalysis.

#### Supercapacitors

SCs store and release energy through the physical adsorption and desorption of electrolyte ions on the electrode surface, and show higher energy density compared with common capacitors [214]. Up to now, SCs have been regarded as a reliable and promising energy storage device due to high power density, environmental friendliness and high safety [215]. MXenes become one of the best electrodes for SCs owing to the moderate specific area, high electronic conductivity, rich ion diffusion channels and large faradaic pseudocapacitance [28, 216]. MXene was first used as electrode material for SCs in 2013 [217]. Yury et al. reported the synthesis of a series of cations (Li^+^, Na^+^, K^+^, NH_4_^+^ and Mg^2+^)-intercalated Ti_3_C_2_T_*x*_ MXene, and a volumetric capacitance of 350 F cm^−3^ can be obtained in KOH electrolyte, which is better than that of traditional porous carbon. Later, freestanding Ti_3_C_2_T_*x*_ films prepared by LiF/HCl etching exhibit a high volumetric capacitance of 900 F cm^−3^ at 2 mV s^−1^ in H_2_SO_4_ electrolyte [65]. Recently, MXenes synthesized by Lewis acidic etching have also been investigated as electrode materials for SCs. For example, Ti_3_C_2_–Cu composites was prepared by CuCl_2_·2H_2_O Lewis acidic salts etching in 2021, and the Cu nanoparticles are tightly anchored on the Ti_3_C_2_T_*x*_ MXene via Ti–O–Cu interfacial bond [122]. Previous studies have shown that O-terminated MXenes exhibit high capacitance due to the easy absorption of H^+^ by –O functional group. The Ti_3_C_2_–Cu electrode is able to deliver a capacitance of 885 F g^−1^ at 0.5 A g^−1^ in H_2_SO_4_ electrolyte. Additionally, the symmetric SCs based on Ti_3_C_2_–Cu achieve a high areal energy density of 103.3 μWh cm^−2^ at 0.8 mW cm^−2^. The superior electrochemical performance of Ti_3_C_2_–Cu electrode is mainly attributed to high content of –O termination, high pseudocapacitive contribution of Cu nanoparticles, boosted charge/mass transfer kinetics and strong interaction between MXene and Cu.

Later, Khan et al. synthesized Ti_3_C_2_Cl_2_, Ti_3_C_2_Br_2_ and Ti_3_C_2_I_2_ MXene via CuCl_2_, CuBr_2_ and CuI molten salts etching, respectively [218]. When used as electrode materials for SCs in 3 M H_2_SO_4_ electrolyte, typical pseudocapacitive-shaped CV curves can be obtained and a specific capacity of 92, 29 and 63C g^−1^ can be afforded for Ti_3_C_2_Cl_2_, Ti_3_C_2_Br_2_ and Ti_3_C_2_I_2_ MXene at 5 mV s^−1^. Further, the capacity retention ratio can reach as high as 85.22% after 10,000 cycles at 1.4 A g^−1^ for Ti_3_C_2_I_2_ electrode, suggesting outstanding long-term cyclic stability. The excellent electrochemical performance of –F, –Br or –I functionalized MXene mainly benefits from the high electrochemical activity of corresponding halogen surface groups. In order to further improve the performance of MXenes produced by Lewis acidic etching, several common and effective strategies such as tuning the interlayer spacing and compositing MXene with other electroactive materials have been proposed by researchers. For example, Zhang et al. reported the synthesis of Ti_3_C_2_Cl_2_ MXene via CdCl_2_ etching in 2022 and then increased the interlayer spacing of MXene by the intercalation of NaCl and cetyltrimethylammonium bromide (CTAB), respectively (Fig. 14g) [219]. As expected, large organic cation CTA^+^ can greatly enlarge the interlayer spacing to 1.16 nm, while the small Na^+^ cation-intercalated MXene shows a value of 1.14 nm (Fig. 14h). The large interlayer spacing of MXene-CTAB can increase the contact area between MXene and electrolyte, boost the ion diffusion kinetics and decrease the charge transfer resistance. Additionally, the CTA^+^ cations can react with the ions between MXene layers, and effectively adsorb counter ions in the electrolyte to deliver additional specific capacitance. Consequently, the Ti_3_C_2_Cl_2_ MXene-CTAB electrode can provide a higher capacitance of 258.28 F g^−1^ in (NH_4_)_2_SO_4_ electrolyte than MXene-NaCl. The highest energy density of MXene-CTAB electrode can reach 11.48 Wh kg^−1^ at a power density of 200 W kg^−1^. More importantly, Ti_3_C_2_Cl_2_ MXene-CTAB exhibits superior cyclic durability with only 7% capacitance loss after 3000 cycles at 1.0 A g^−1^, which is superior to that of Ti_3_C_2_Cl_2_ MXene and Ti_3_C_2_Cl_2_ MXene-NaCl.

LDHs have been regarded as promising electrode materials for SCs owing to their high specific capacitance and energy density [220]. However, low electronic conductivity of LDHs results in inferior rate capability and cyclic performance [221]. Hybridizing LDHs with highly conductive MXenes may lead to great improvement in electrode performance. In 2022, Zhao et al. firstly prepared Ti_3_C_2_Cl_2_ MXene nanodots (QD-Ti_3_C_2_Cl_2_) via CdCl_2_ etching and subsequent hydrothermal reaction, and the nanodots are distributed on the surface of MXene nanosheets [222]. Then, the negatively-charged QD-Ti_3_C_2_Cl_2_ can integrate with positively-charged NiAl-LDHs to electrostatically fabricate QD-Ti_3_C_2_Cl_2_@NiAl-LDHs composites, where the NiAl-LDHs nanosheets are dispersed on MXene nanosheets (Fig. 14i). Derived from the superior charge transfer kinetics of QD-Ti_3_C_2_Cl_2_, a small band gap of 0.52 eV can be obtained for QD-Ti_3_C_2_Cl_2_@NiAl-LDHs, which is lower than that of NiAl-LDHs (1.51 eV), demonstrating enhanced electronic conductivity. Additionally, compared with NiAl-LDHs, QD-Ti_3_C_2_Cl_2_@NiAl-LDHs hybrids possess a higher specific area of 658.74 m^2^ g^−1^ and lower Gibbs free energy of redox reaction (−0.355 eV) (Fig. 14j), greatly accelerating the mass transport and adsorption of active species. Therefore, the obtained QD-Ti_3_C_2_Cl_2_@NiAl-LDHs electrode achieves a pretty high capacitance of 2010.8 F g^−1^ at 1.0 A g^−1^ (Fig. 14k), and excellent long-term cyclic performance with 94.1% capacitance retention after 10,000 cycles, which is better than those of NiAl-LDHs electrode. Additionally, a large energy density of 100.5 Wh kg^−1^ can be delivered for QD-Ti_3_C_2_Cl_2_@NiAl-LDHs at a power density of 299.8 W kg^−1^. The above examples reveal that MXenes prepared by Lewis acidic etching are promising anodes for electrochemical energy storage systems.

### Energy Conversion

Exploring renewable energy is one of the greatest challenges for the sustainable development of contemporary society. Advanced energy conversion systems such as photovoltaic electrodes, catalysis, and fuel cell, will play a crucial role in the future [223]. For example, photovoltaic devices such as silicon solar cells have been commercialized on a large scale and dominate the market in China, and perovskite solar cells (PSCs) are attracting increasing attention. Additionally, several common and classical catalysis reactions including hydrogen evolution reaction (HER), oxygen evolution reaction (OER), carbon dioxide reduction reaction (CO_2_RR) and nitrogen reduction reaction (NRR) have also been widely investigated in the past decades [224].

#### Perovskite Solar Cells

**Fig. 15:**
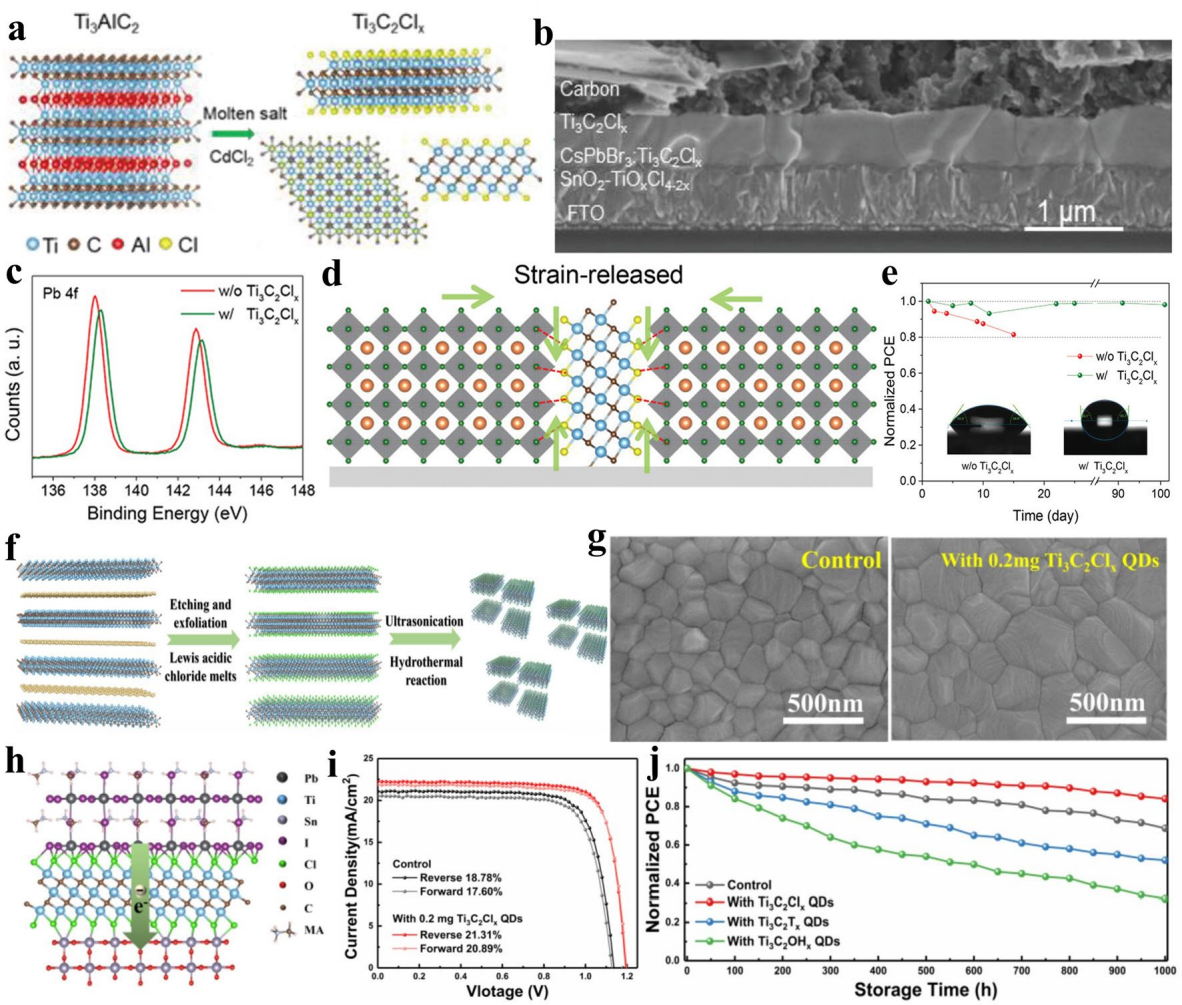
**a** Schematic showing the preparation of Ti_3_C_2_Cl_*x*_ MXene. **b** Cross-sectional SEM image of all-inorganic PSCs. **c** Pb 4f XPS spectra of CsPbBr_3_ perovskite film. **d** Schematic of released strain in the CsPbBr_3_ grains with Ti_3_C_2_Cl_*x*_ MXene. **e** Long-term stability of the devices in air under 80% relative humidity and 25 °C. Reproduced with permission from [159] Copyright 2021, The Authors, published by Wiley–VCH. **f** Schematic demonstrating the preparation of Ti_3_C_2_Cl_*x*_ QDs. **g** SEM images of pristine and Ti_3_C_2_Cl_*x*_ QDs-modified perovskite films. **h** Schematic illustration of the perovskite-Ti_3_C_2_Cl_*x*_ QDs-SnO_2_ heterojunction. **i** Reverse and forward scans for the pristine and Ti_3_C_2_Cl_*x*_ QDs-modified devices. **j** Normalized PCEs against storage time for the pristine and Ti_3_C_2_Cl_*x*_ QDs-treated devices. Reproduced with permission from [228] Copyright 2021, Elsevier

Compared with silicon solar cells, PSCs possess the advantages of low cost, high absorption coefficient, easy preparation and high theoretical photoelectric conversion efficiency (PCE). In recent years, a high PCE value of 25.7% can be obtained for PSCs, which makes PSCs become one of the most promising photovoltaic devices in the near future [225]. However, defective interfaces and grain boundaries of PSCs greatly deteriorate device performance [226]. Recently, MXenes have been intensively researched in PSCs as additive, electrode or hole/electron transport layer owing to their high electrical conductivity and carrier mobility, adjustable work function, abundant surface functional groups and excellent mechanical property [227]. In 2021, Zhou et al. synthesized Ti_3_C_2_Cl_*x*_ MXene suspension via CdCl_2_ etching and following IPA intercalation, and the Ti_3_C_2_Cl_*x*_ MXene as addictive is then incorporated into the bulk and surface of perovskite film (Fig. 15a–b) [159]. The peak shift in Pb 4f XPS spectrum indicates that the -Cl surface group with strong electron-withdrawing ability in Ti_3_C_2_Cl_*x*_ MXene and the unsaturated Pb^2+^ in CsPbBr_3_ can form Pb-Cl bond along the interface (Fig. 15c). The strong interaction between Ti_3_C_2_Cl_*x*_ MXene and CsPbBr_3_ can effectively relieve the lattice strain of perovskite (Fig. 15d), and the defects situated at the interface and grain boundaries can be healed. Therefore, a high PCE of 11.08% and open-circuit voltage of 1.702 V can be obtained for the fabricated all-inorganic CsPbBr_3_ PSCs. More importantly, the Ti_3_C_2_Cl_*x*_-functionalized CsPbBr_3_ devices exhibit outstanding long-term stability of 100 days under 80% relative humidity and 25 °C (Fig. 15e), demonstrating the high-quality of perovskite films.

Later, Ti_3_C_2_Cl_*x*_ quantum dots (Ti_3_C_2_Cl_*x*_ QDs) with an average size of around 8 nm were prepared through CuCl_2_ etching, subsequent hydrothermal reaction and ultrasonication treatment (Fig. 15f) [228]. As followed, the Ti_3_C_2_Cl_*x*_ QDs is introduced into the precursor solution to fabricate perovskite layer via one-step processing. Similarly, the -Cl functional groups in Ti_3_C_2_Cl_*x*_ QDs can generate chemical bond with Pb^2+^ cations in Rb_0.05_Cs_0.05_(FA_0.83_MA_0.17_)_0.90_Pb(I_0.83_Br_0.17_)_3_ perovskite compound, which can also be confirmed by the peaks move in Pb 4f and Cl 2*p* XPS spectra. Additionally, scanning electron microscopy (SEM) images demonstrate that the perovskite grain size can be enlarged from 273 to 453 nm after the addition of Ti_3_C_2_Cl_*x*_ QDs (Fig. 15g), reducing the number of grain boundary. The introduction of Ti_3_C_2_Cl_*x*_ QDs acting as nucleation sites can effectively slow down the growth of perovskite crystal and the film crystallinity can be greatly enhanced with an optimized orientation, thus leading to fewer crystal distortion. Moreover, the interfacial charge transfer resistance between perovskite layer and SnO_2_ electron transport layer can be greatly reduced owing to the highly conductive Ti_3_C_2_Cl_*x*_, and the energy level alignment can be further improved (Fig. 15h). Consequently, the Ti_3_C_2_Cl_*x*_ QDs-modified device can deliver a high PCE value of 21.31% and an open-circuit voltage of 1.19 V (Fig. 15i). Furthermore, the extraordinary long-term stability with 84% PCE retention after 1000 h storage time under 40% relative humidity can be afforded for Ti_3_C_2_Cl_*x*_ QDs-modified device (Fig. 15j). The two works indicate that the Cl-terminated MXenes prepared by Lewis acidic etching has great potential to enhance the performance of PSCs devices.

#### Hydrogen Evolution Reaction

H_2_ is regarded as a clean and renewable energy source that can replace fossil fuels in the future owing to its high heat value and pollution-free combustion product. The HER is an effective method to produce high-purity H_2_ via water splitting [229]. Some noble catalysts such as Au, Pt and Pd have been widely used to accelerate the H_2_ evolution kinetics and decrease overpotentials because of their high activities [230]. Nevertheless, high price and low reserve greatly impede the large-scale application of noble catalysts. In recent years, MXenes have been considerably researched as catalyst derived from their 2D layered structure, tailorable surface chemistry, diverse composition, high electrical conductivity and superior mechanical stability [231, 232]. Particularly, the polar terminations of MXenes can strongly interact with other materials to form high-performance catalysts. For example, Jiang et al. firstly synthesized Ti_3_CNCl_2_ MXene via NiCl_2_ molten salt etching of Ti_3_AlCN MAX. Then, Ti_3_CNCl_2_@CoS_2_ core–shell architecture as efficient HER catalyst can be obtained by hydrothermal reaction [233]. Theoretical calculations reveal that electron can transfer from Ti_3_CNCl_2_ MXene to CoS_2_ through -Cl functional group, demonstrating strong interaction between them. Additionally, the Gibbs free energy of H_2_ adsorption of Ti_3_CNCl_2_@CoS_2_ hybrids is close to zero, indicating a strong catalytic activity. It is noteworthy that the electrons of Ti_3_CNCl_2_@CoS_2_ become more delocalized, which suggests that H^+^ cations are easy to combine with electrons. As a result, benefiting from the synergistic effect of CoS_2_ and highly-conductive Ti_3_CNCl_2_, the constructed Ti_3_CNCl_2_@CoS_2_ hybrids deliver a low Tafel slope of 89 mV dec^−1^ and small charge transfer resistance in 0.5 M H_2_SO_4_ electrolyte. Moreover, boosted long-term stability can be obtained for Ti_3_CNCl_2_@CoS_2_, which can sustain catalytic activity for 10 h and the current density shows almost no attenuation.

#### Overall Water Splitting

Water splitting provides a simple, effective and promising method for H_2_ and O_2_ production, which can effectively solve the energy crisis [234]. In general, electrolysis of water is composed of HER at the cathode and the OER at the anode and requires a theoretical voltage of 1.23 V [235]. Nevertheless, the multi-electron reaction results in sluggish kinetics, which greatly limits the H_2_ and O_2_ production and increases the drive voltage [236]. Exploring high-performance electrocatalysts can effectively solve the above-mentioned issues. For example, Sarfraz et al. reported the synthesis of Cl-terminated Ti_3_C_2_Cl_2_ MXene via CuCl_2_ etching of Ti_3_AlC_2_ precursor in 2022, and Ti_3_C_2_Cl_2_ MXene with obvious lamellar structure is then used as catalyst for water splitting in 1 M KOH solution [237]. Compared with Ti_3_C_2_T_*x*_ MXene obtained by HF etching (HF-Ti_3_C_2_T_*x*_ MXene), Ti_3_C_2_Cl_2_ MXene possesses an enlarged interlayer spacing, more active sites and stable architecture. Therefore, Ti_3_C_2_Cl_2_ electrode shows a smaller overpotential of 330 mV than HF-Ti_3_C_2_T_*x*_ MXene (390 mV) at 30 mA cm^−2^ for OER. Similarly, a lower HER overpotential of 259 mV can be obtained for Ti_3_C_2_Cl_2_ at 10 mA cm^−2^, while HF-Ti_3_C_2_T_*x*_ MXene demonstrates a large overpotential of 444 mV at the same current density. Notably, Ti_3_C_2_Cl_2_ electrode exhibits a low Tafel slope of 48 and 92 mV dec^−1^ for OER and HER, respectively, indicating excellent catalytic activity for water splitting.

#### Carbon Dioxide Reduction Reaction

**Fig. 16:**
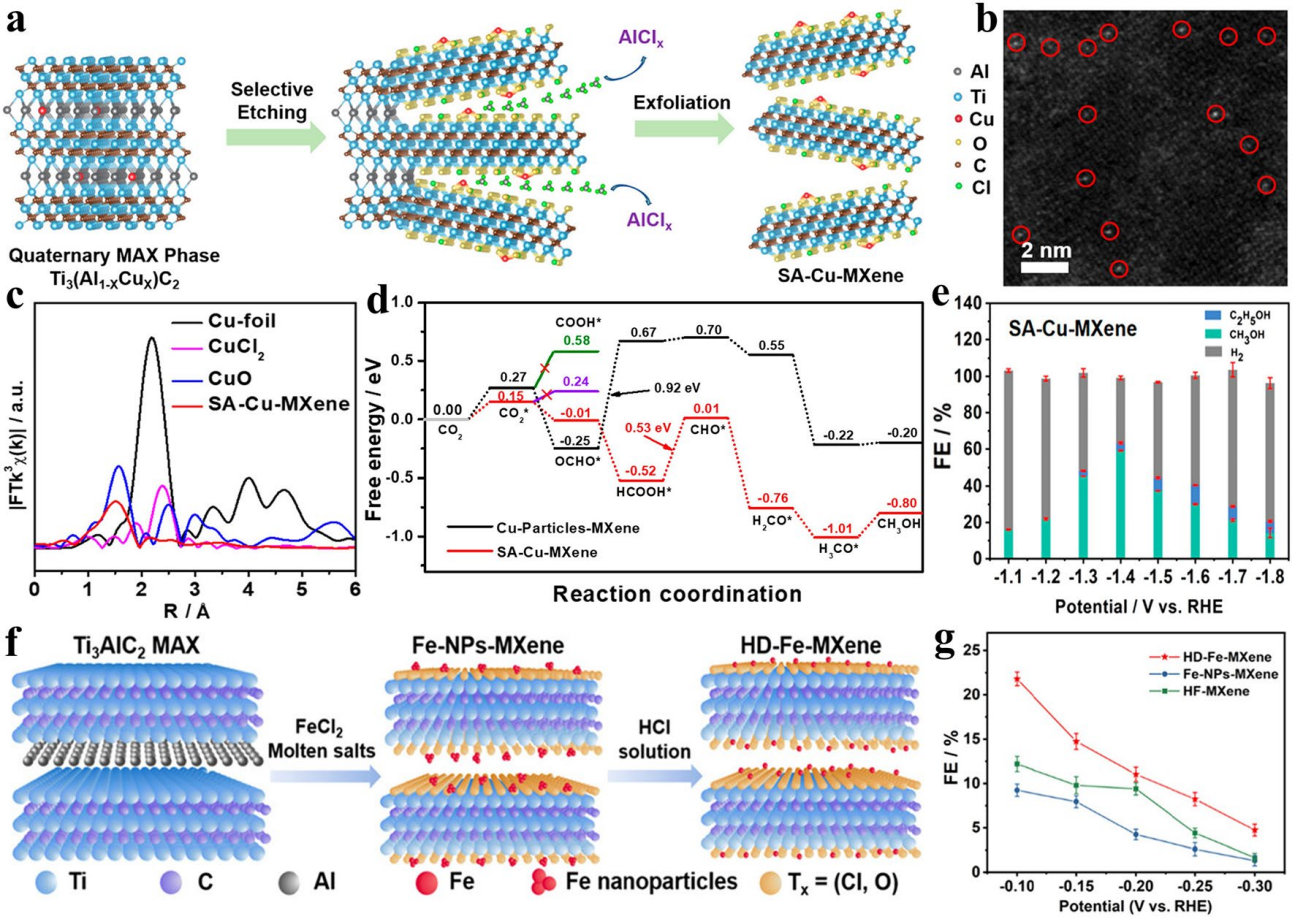
**a** Schematic of the synthesis of SA-Cu-MXene. **b** HAADF-STEM image of SA-Cu-MXene. **c** XAS spectra of SA-Cu-MXene. **d** Free energy diagram of CO_2_ to CH_3_OH. **e** Faradaic efficiency of SA-Cu-MXene at various potentials. Reproduced with permission from [158] Copyright 2021, American Chemical Society. **f** Schematic of the preparation of HD-Fe-MXene. **g** Faradaic efficiency of different materials at various potentials. Reproduced with permission from [244] Copyright 2022, The Authors, published by Wiley–VCH

In recent years, the depletion of fossil fuels has produced a large amount of CO_2_, leading to a huge impact on the environment, such as greenhouse effect and the extinction of some species [238]. Therefore, it is necessary to reduce CO_2_ emission or find effective ways to use it. The CO_2_RR is an effective and green method to convert CO_2_ into high-value chemicals or fuels [239]. However, CO_2_RR suffers from sluggish kinetics owing to the good stability of CO_2_, and efficient catalysts are therefore required [240]. In 2021, Zhao et al. synthesized single atom Cu immobilized Ti_3_C_2_Cl_*x*_ MXene (SA-Cu-MXene) as CO_2_RR electrocatalyst via ZnCl_2_ molten salt etching of Ti_3_(Al_1−*x*_Cu_*x*_)C_2_ MAX (Fig. 16a) [158]. It is worth mentioning that the Cu atoms can be well preserved during the selective etching process. The content of Cu single atoms is approximately to be 1.0 wt% and many bright dots representing Cu atoms can be observed in HAADF-STEM image (Fig. 16b). Additionally, XAS measurement indicates that Cu atoms are coordinated by –O surface groups of Ti_3_C_2_Cl_*x*_ MXene via Cu–O bond (Fig. 16c). Owing to the superior charge transfer kinetics of MXene and unsaturated electronic structure of Cu atoms, the obtained catalyst can greatly reduce the energy barrier from CO_2_ to CH_3_OH and deliver a maximum Faradaic efficiency value of 59.1% at −1.4 V (Fig. 16d–e), demonstrating outstanding catalytic capability. More importantly, a high Faradaic efficiency of over 58% can still be obtained after 30 h, which indicates enhanced stability.

#### Nitrogen Reduction Reaction

NH_3_, as a particularly important chemical material, has played a key role in industry and agriculture. At present, large-scale NH_3_ synthesis mainly relies on the traditional Haber–Bosch process, which requires to be carried out under high temperature and pressure (300–500 °C and 150–200 atm) [241]. In short, Haber–Bosch process not only consumes huge energy but also releases plenty of greenhouse gases into the atmosphere. The electrocatalytic NRR has attracted intensive attention as a green method to produce NH_3_ at room temperature and atmospheric pressure [242]. Nevertheless, promising electrocatalysts with superior stability and high activity are also required to promote the NRR process, which suffers from a large overpotential and low yield of NH_3_ owing to the strong N≡N bond in N_2_ [243]. For example, Wang et al. prepared highly dispersed Fe immobilized in fluorine-free Ti_3_C_2_T_*x*_ MXene (HD-Fe-MXene) as NRR electrocatalyst in 0.1 M Na_2_SO_4_ electrolyte through FeCl_2_ etching and HCl washing (Fig. 16f) [244]. The XRD pattern indicates that the formed Fe nanoparticles can be eliminated by HCl, and energy-dispersive spectroscopy and XPS analysis demonstrate that the remaining Fe atoms with a content of 3.12 wt% are highly anchored on the surface of Ti_3_C_2_T_*x*_ MXene via Fe–O bond. Benefiting from the rich active sites and outstanding electrical conductivity of Fe atoms and fluorine-free Ti_3_C_2_T_*x*_ MXene, the adsorption and activation of N_2_ as well as charge transfer kinetics can be considerably improved. Consequently, the obtained electrocatalyst can afford a high NH_3_ yield of 18.25 µg h^−1^ mg^−1^ and a Faradaic efficiency of 21.8% (Fig. 16g). Additionally, boosted stability with almost unchanged current density after 24 h testing can be obtained for HD-Fe-MXene electrode.

### Sensors

**Fig. 17:**
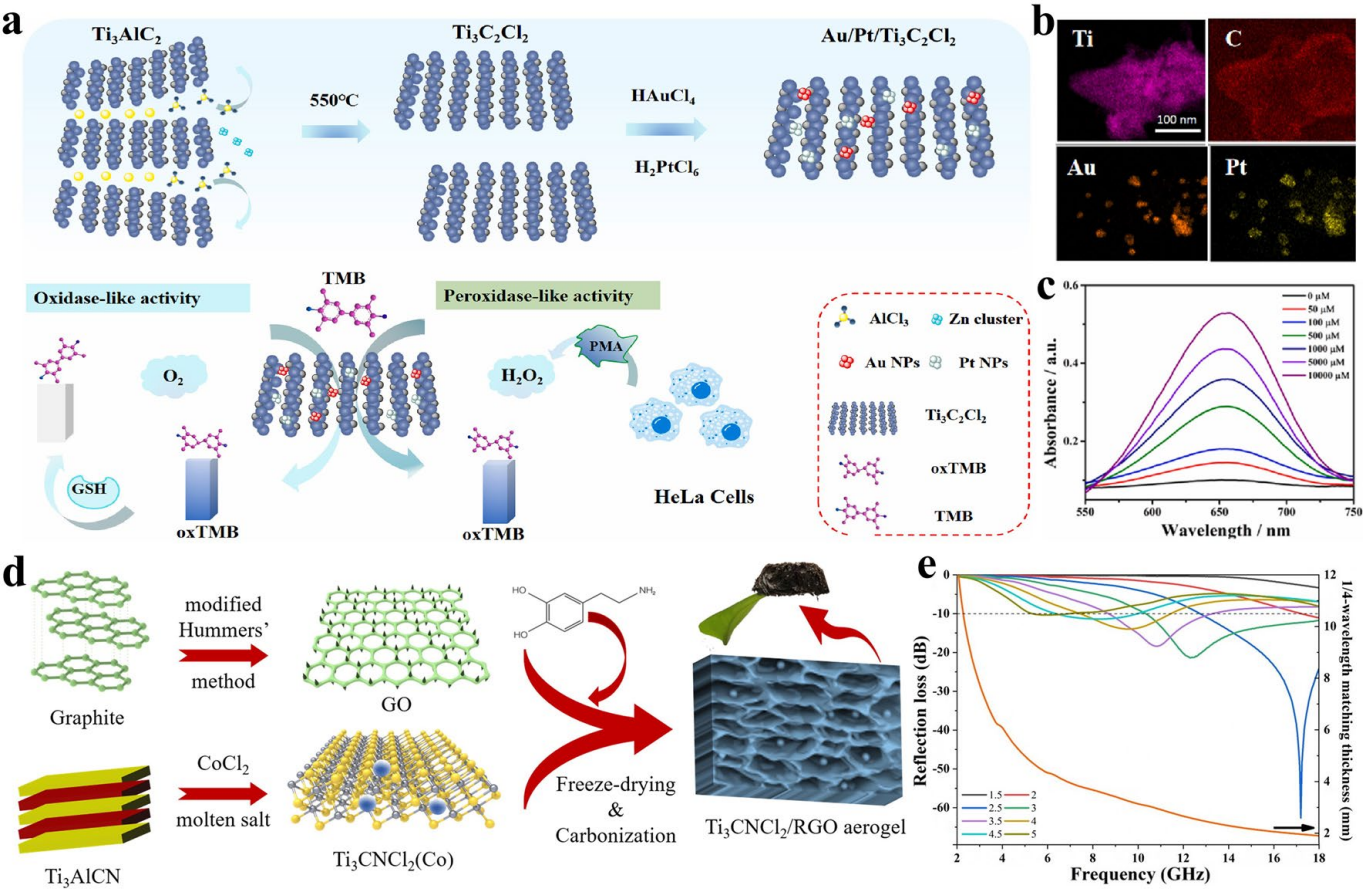
**a** Schematic demonstrating the synthesis of Au/Pt/Ti_3_C_2_Cl_2_ hybrids for in-situ detection of H_2_O_2_ and colorimetric detection of glutathione. **b** EDS element mapping of Au/Pt/Ti_3_C_2_Cl_2_ composites. **c** Absorption spectra of Au/Pt/Ti_3_C_2_Cl_2_ nanocomposites system with increasing concentrations of H_2_O_2_. Reproduced with permission from [247] Copyright 2022, Elsevier. **d** Schematic representing the preparation of Ti_3_CNCl_2_/RGO aerogel. **e** Reflection loss of Ti_3_CNCl_2_/RGO. Reproduced with permission from [250] Copyright 2021, Elsevier

In modern society, sensors play an increasingly significant role in environmental protection, disease detection and industrial production, etc. Currently, there are different types of sensors, such as gas sensors, biosensors and sound sensors [245]. Owing to the medium specific surface area, layered structure, rich active sites and superior electronic conductivity, MXenes have been regarded as promising sensitive materials in recent years [44]. For example, Wu et al. synthesized Ti_3_C_2_Cl_2_ MXene via ZnCl_2_ etching in 2021 and investigated its gas-sensing performance [246]. They found that Ti_3_C_2_Cl_2_ MXene exhibits a response value of 13.2% to NH_3_, indicating the potential of Ti_3_C_2_Cl_2_ MXene in gas sensors. In addition, Au/Pt/Ti_3_C_2_Cl_2_ hybrids as sensors were fabricated by ZnCl_2_ etching and subsequent self-reduction process (Fig. 17a) [247]. The Ti species with low-valence can effectively reduce Au^3+^ and Pt^4+^ cations to Au and Pt nanoparticles, respectively, which are then distributed on the surface of Ti_3_C_2_Cl_2_ nanosheets (Fig. 17b). By virtue of the superior catalytic activity of obtained Au/Pt/Ti_3_C_2_Cl_2_ composites, a colorimetric sensing platform is constructed to in-situ detect the H_2_O_2_ from HeLa cells. The fabricated sensor possesses a wide detection range of 50–10,000 μM and detection limit of 10.24 μM (Fig. 17c), demonstrating promising application in intracellular biosensing.

### Microwave Absorption

With the rapid development of information technology in recent years, smart devices based on electromagnetic wave have been utilized more and more frequently, thus resulting in increasingly serious electromagnetic pollution [248, 249]. Therefore, exploring advanced microwave absorption materials is becoming increasingly important. MXenes demonstrate great potential in the fields of microwave absorption based on the following aspects: (1) The layered architecture can realize multiple reflection and scattering of electromagnetic wave; (2) The high electronic conductivity endows MXenes with high dielectric loss; (3) The surface groups of MXenes will produce dipole under electromagnetic field, which further strengthens the dielectric loss [52]. It is noteworthy that MXenes/metal hybrids obtained by one-step Lewis acidic etching can exhibit good microwave absorption performance due to the addition of ferromagnetic metals. For example, Wang et al. firstly prepared Ti_3_CNCl_2_(Co) hybrids via CoCl_2_ etching, and Ti_3_CNCl_2_(Co) composites were then distributed on the surface of reduced graphene oxide (RGO) nanosheets to form Ti_3_CNCl_2_-decorated RGO aerogel via the crosslinking effect of dopamine hydrochloride (Fig. 17d) [250]. Benefiting from the conductivity loss of 3D interconnecting conductive network, improved polarization loss of abundant heterogeneous interfaces and surface functional groups as well as magnetic loss of Co nanoparticles, the Ti_3_CNCl_2_/RGO aerogel can deliver a minimum reflection loss intensity of -62.62 dB at 17.2 GHz with a thickness of 2.5 mm (Fig. 17e), indicating its boosted microwave absorbing ability.

Table 3 provides an overall summary of the performance of MXenes and MXene-based materials in the fields of LIBs, SIBs, ZIBs, Li–S batteries, metal anodes, DIBs, SCs, PSCs, HER, overall water splitting, CO_2_RR, NRR, sensors and microwave absorption.

**Table 3:** Summary of the performance of MXenes and MXene-based materials prepared by Lewis acidic etching route

Materials	Application	Performance	Refs
Ti_3_C_2_T_*x*_	LIBs	205 mAh g^−1^ at 0.6C	[67]
e-MS-Ti_3_C_2_T_*x*_	LIBs	225 mAh g^−1^ at 0.2 A g^−1^, 95 mAh g^−1^ at 16 A g^−1^	[153]
Ti_3_C_2_Br_*x*_	LIBs	189 mAh g^−1^ at 0.05 A g^−1^	[178]
Ti_3_C_2_T_*x*_/Sn	LIBs	226.2 mAh g^−1^ after 1,000 cycles at 0.2 A g^−1^	[145]
Ti_2_CT_*x*_	LIBs	280 mAh g^−1^ at 0.1 A g^−1^, 160 mAh g^−1^ at 2 A g^−1^	[112]
Ti_2_CT_*x*_	LIBs	280 mAh g^−1^ at 0.1 A g^−1^	[175]
Ti_2_NT_*x*_	LIBs	350 mAh g^−1^ after 1200 cycles at 1 A g^−1^	[176]
Nb_2_CT_*x*_	LIBs	330 mAh g^−1^ at 0.05 A g^−1^, 80 mAh g^−1^ at 10 A g^−1^	[119]
Ti_3_C_2_T_*x*_/FeS_2_	SIBs	474.9 mAh g^−1^ after 600 cycles at 5 A g^−1^	[147]
Ti_3_C_2_T_*x*_/CuS	SIBs	347 mAh g^−1^ after 800 cycles at 3 A g^−1^	[148]
Fe_2_O_3_@Ti_3_C_2_T_*x*_	SIBs	350 mAh g^−1^ after 200 cycles at 1.0 A g^−1^	[180]
Ti_3_C_2_I_2_	ZIBs	181 mAh g^−1^ at 0.25 A g^−1^	[123]
Ti_3_C_2_I_2_	ZIBs	207 mAh g^−1^ at 0.5 A g^−1^, 126 mAh g^−1^ at 5 A g^−1^	[194]
SA-Zn-MXene	Li–S	80% retention after 200 cycles at 4C	[157]
N-MX-CoS_2_	Li–S	651 mAh g^−1^ after 700 cycles at 1C	[146]
Zn-MXene	Li metal	11.3 ± 0.1 mV overpotential, 1,200 h cycle life	[156]
Ti_3_C_2_Cl_2_	Zn metal	840 h at 2 mA cm^−2^ with a capacity of 1 mAh cm^−2^	[212]
Ti_3_C_2_Cl_2_	DIBs	141 mAh g^−1^ after 1000 cycles at 1 A g^−1^	[113]
Ti_3_C_2_Cl_2_	SCs	92 C g^−1^ at 5 mV s^−1^	[218]
Ti_3_C_2_Cl_2_-CTAB	SCs	7% capacitance loss after 3,000 cycles at 1.0 A g^−1^	[219]
N-containing Ti_3_C_2_T_*x*_	SCs	303 F g^−1^ at 2 V s^−1^	[126]
Ti_3_C_2_-Cu	SCs	103.3 μWh cm^−2^ at 0.8 mW cm^−2^	[122]
QD-Ti_3_C_2_Cl_2_			
@NiAl-LDHs	SCs	2010.8 F g^−1^ at 1.0 A g^−1^	[222]
Ti_3_C_2_Cl_*x*_	PSCs	11.08% conversion efficiency, 1.702 V voltage	[159]
Ti_3_C_2_Cl_*x*_ QDs	PSCs	21.31% conversion efficiency, 1.19 V voltage	[228]
Ti_3_CNCl_2_@CoS_2_	HER	Tafel slope of 89 mV dec^−1^	[233]
Ti_3_C_2_T_*x*_:Co	HER	103.6 mV overpotential at 10 mA cm^−2^	[232]
Ti_3_C_2_Cl_2_	Water splitting	330 mV overpotential at 30 mA cm^−2^ for OER 259 mV overpotential at 10 mA cm^−2^ for HER	[237]
SA-Cu-MXene	CO_2_RR	59.1% Faradaic efficiency	[158]
HD-Fe-MXene	NRR	21.8% Faradaic efficiency	[244]
Ti_3_C_2_Cl_2_	Sensors	13.2% response to NH_3_	[246]
Au/Pt/Ti_3_C_2_Cl_2_	Sensors	Wide detection range of 50–10,000 μM	[247]
Ti_3_CNCl_2_/RGO	Microwave absorption	− 62.62 dB at 17.2 GHz	[250]

## Summary and Perspectives

In the past eleven years, great progress has been achieved in the development of MXenes, which can be effectively confirmed by the increasing publications. Notably, the compositional and structural diversity of MXenes have been considerably expanded. In terms of composition, M-site atoms, X-site atoms and surface groups all exhibit rich selection space. Structurally, *i*-MXenes, *o*-MXenes and high-entropy MXenes have also been extensively explored. It can be anticipated that if an efficient and general preparation method is applied, plenty of new MXenes with various compositions and structures can be successfully obtained, and they may demonstrate some fascinating properties. Therefore, exploring a safe, green, non-hazardous, low-cost and easily scalable synthesis method has always been the research focus in the MXene community. Up to now, many preparation strategies for MXenes have been proposed, such as HF etching, in-situ HF etching, bifluoride salts etching, electrochemical etching, alkali etching, common molten salts etching, ionic liquids etching, halogen etching and chemical vapor deposition. Nevertheless, they all have their own shortcomings and are unsuitable for large-scale application (Table 1). For example, HF etching, in-situ HF etching and alkali etching all exhibit high or medium experimental risk. Additionally, bifluoride salts etching, alkali etching, common molten salts etching, ionic liquids etching and halogen etching demonstrate poor universality. More importantly, the MXenes prepared by above-mentioned methods fail to demonstrate controllable and uniform terminations. Lewis acidic etching, as an emerging preparation strategy for MXenes, has attracted increasing attention in the past three years owing to a series of merits, such as significantly boosted safety, superior generality, the ability to endow MXenes with uniform surface groups and the potential for large-scale application.

In this review, we have systematically introduced the Lewis acidic molten salts etching strategy from four aspects: etching mechanism, terminations regulation, in-situ formed metals and delamination of multi-layered MXenes. Briefly, the etching mechanism is mainly based on the replacement reaction between metal cations in Lewis acidic molten salts and zero-valence A-site atoms in MAX precursor. The difficulty degree of etching reaction mainly depends on the redox potentials of molten cations and A-site atoms, but has nothing to do with the M, X-site atoms and *n* value in MAX phase. A simple criterion is that the metal cations in Lewis acidic salts with high redox potentials can readily etch the MAX phases with low redox potential A-site atoms. Accordingly, some nitride MAX including Ti_2_AlN and Ti_4_AlN_3_ and non-Al MAX such as Ti_3_SiC_2_ and Ta_2_GaC with high exfoliation energy can be successfully etched by various Lewis acidic molten salts. Additionally, uniform Cl, Br or I-terminated MXenes is able to be obtained by Lewis acidic etching, and they all exhibit metallic-level conductivities and even superconductivity in the low-temperature region. These redox-active groups-terminated MXenes have been investigated in SCs and advanced batteries. More importantly, halogen groups such as –Cl and –Br can be substituted by S^2−^, Se^2−^, Te^2−^, N^3−^ and NH_2_^−^ anions from Lewis bases molten salts via nucleophilic reaction owing to the low dissociation energies of Ti–Br and Ti–Cl bond, really realizing the controllable terminations. Further, the in-situ generated metal nanoparticles can be tightly anchored among the MXenes substrate during the etching process. The rational and effective application of MXenes/metals and one-step sulfidation treatment-obtained MXenes/metal sulfides composites in LIBs, SIBs, Li–S batteries, SCs and HER have been demonstrated. Subsequently, the multi-layered MXenes obtained by Lewis acidic etching have been delaminated into few-layered nanosheets via *n*-BuLi, IPA, TBAOH, DMSO or i-PrA intercalation. Finally, the application of obtained MXenes and MXene-based hybrids via Lewis acidic etching route in LIBs, SIBs, ZIBs, Li–S batteries, metal anodes, DIBs, SCs, PSCs, HER, overall water splitting, CO_2_RR, NRR, sensors and microwave absorption have been carefully summarized. Up to now, most applications are concentrated in the fields of energy storage and conversion. However, the development of Lewis acidic etching is still in its infancy, and more efforts are required to fully understand this method and promote its widespread application. Some main challenges and opportunities are listed as follows:

1. At present, there are still some details to be researched during the Lewis acidic etching process. For example, the reaction between metal cations in Lewis acidic molten salts and M species in MAX phase during the etching process has been confirmed, which generally results in the formation of single atom-implanted MXenes. However, the specific mechanism and the difficulty degree of above reaction still require further exploration through a lot of experiments and theoretical calculations, which is conducive to the controlled preparation of single atom-implanted MXenes. In addition, a recent study has proved that NaCl can not only provide molten salts environment during the etching process, but also act as pore-forming agent to form Ti_3_C_2_Cl_2_ MXene with in-plane porosity. In the future, more attention should be paid to investigate the universality of NaCl as pore-forming agent, which can greatly promote the preparation of other in-plane porous MXenes through Lewis acidic etching. Notably, the in-situ formed metal nanoparticles are usually aggregated during the cooling process and then anchor on the surface of MXenes. It is unclear whether there are some metal clusters in the interlayer of MXenes. Furthermore, preventing the agglomeration of metal nanoparticles is also worth investigating.
2. In the future, more researches should be carried out to synthesize nitride MXenes via Lewis acidic etching. It can be expected that more nitride MXenes will be successfully prepared due to the superior universality of Lewis acidic etching. Currently, Lewis acidic molten salts are mainly used to etch MAX phase precursors. The future studies are needed to investigate that whether non-MAX ternary layered compounds such as Mo_2_Ga_2_C can be etched by Lewis acidic salts. Further, transition metal borides (MAB) such as MoAlB and Cr_2_AlB_2_, which is similar to MAX phase, have attracted intensive interests in the past few years. Therefore, the universal preparation of MBene by etching MAB precursors with Lewis acidic molten salts is worthy of in-depth investigation. Finally, the parameters of Lewis acidic etching reaction, including the molar ratio of MAX precursor and Lewis acidic salts, reaction temperature, time and atmosphere, are widely different in reported literatures (Table 2). More efforts are still required to optimize the etching parameters on the premise of ensuring the successful preparation of MXenes, such as selecting low-cost Lewis acidic salts, decreasing the amount of Lewis acidic salts and reaction temperature and changing the reaction atmosphere from protective gas to air, which is beneficial for the large-scale preparation of MXenes via Lewis acidic etching.
3. Although MXenes prepared by Lewis acidic etching can be theoretically terminated by uniform halogen groups, some halogen terminations will inevitably be replaced by –O in air atmosphere or during the washing process. In addition, the surface group substitution/elimination reactions in molten inorganic salts are carried out in Ar-filled glovebox in order to avoid the competition between –O functional group and other terminations. In the future, it is an urgent issue to maintain uniform termination for MXenes obtained by Lewis acidic etching under air atmosphere. All in all, combined with Lewis acidic etching, termination exchange reaction in Lewis bases molten salts medium can indeed endow MXenes with unconventional terminations. Therefore, much work should be needed to explore the influence of new terminations (S, Se, Te, N, NH, etc.) on the properties of various MXenes, such as electronic, optical and mechanical, magnetic properties as well as ion migration kinetics, hydrophilicity, chemical and thermal stability, etc.
4. So far, MXenes/metals hybrids prepared by Lewis acidic etching have been successfully applied in LIBs and SCs. In the future, MXenes/metals composites exhibit great research potential in catalysis, metal anodes and other fields. For example, benefiting from the unique layered structure and superior charge/mass transfer kinetics of MXenes as well as nucleation induction effect of some metals (Co, Sn, etc.), MXenes/metals hybrids can be utilized as three-dimensional frameworks to suppress the dendrite growth of metal anodes (Li, Na, K, Zn, etc.). Furthermore, the obtained MXenes/metals hybrids can be transformed to MXenes/metal compounds composites via one-step sulfurization, phosphorization, selenization or tellurization treatment. Currently, most of the research was concentrated in MXenes/metal sulfides hybrids, such as N-MX-CoS_2_ and Ti_3_C_2_T_*x*_/FeS_2_. In the future, more studies should be performed to prepare MXenes/metal phosphides, MXenes/metal selenides or MXenes/metal tellurides hybrids via Lewis acidic etching route, which can be rationally and effectively applied in LIBs, SIBs, potassium-ion batteries and so on.
5. Up to now, most of MXenes prepared by Lewis acidic etching are still at multi-layered state (Table 2). The absence of hydrophilic terminations such as –OH makes it difficult for the obtained multi-layered MXenes to be dispersed in water and then intercalated by large organic molecules, thus reducing the delamination efficiency and exhibiting a low yield of few-layered and single-layered nanosheets. Although some attempts have been made, such as improving the hydrophilicity of MXenes by KOH treatment, or intercalating MXenes by *n*-BuLi in organic solvent NMF, there is still no safe, green, efficient and easily scalable method to delaminate the Lewis acidic molten salts-etched multi-layered MXenes. In the future, great efforts should be made to explore other appropriate delamination methods, such as high-energy ball milling and electrochemical delamination.

In conclusion, although some progress has been made in the development of Lewis acidic etching, it is still in the early stage. In order to solve the above-mentioned challenges, much more work should still be done in the near future. It can be anticipated that Lewis acidic etching will experience a rapid development period, which can attract more and more researchers in MXene community to widely utilize this emerging preparation strategy.
